# Synthesis and characterization of novel combretastatin analogues of 1,1-diaryl vinyl sulfones, with antiproliferative potential via in-silico and in-vitro studies

**DOI:** 10.1038/s41598-022-05958-6

**Published:** 2022-02-03

**Authors:** Godshelp O. Egharevba, Ahmed Kamal, Omotayo O. Dosumu, Sunitha Routhu, Olatomide A. Fadare, Stephen O. Oguntoye, Stanislaus N. Njinga, Abimbola P. Oluyori

**Affiliations:** 1 Industrial Chemistry Programme, Department of Physical Sciences, College of Pure and Applied Sciences, Landmark University, Omu-Aran, Kwara State Nigeria; 2 Medicinal Chemistry and Pharmacology Division, CSIR-Indian Institute of Chemical Technology, Hyderabad, 500007 India; 3 Landmark University SDG 3 (Good Health and Well-being), Omu-Aran, Nigeria; 4 Landmark University SDG 12 (Responsible Consumption and Production), Omu-Aran, Nigeria; 5 Birla Institute of Technology and Science, Pilani, Hyderabad Campus, India; 6 Department of Industrial Chemistry, University of Ilorin, P.M.B. 1515, Ilorin, Nigeria; 7 Department of Chemistry, Obafemi Awolowo University, Ile-Ife, Osun State Nigeria; 8 Department of Chemistry, University of Ilorin, P.M.B. 1515, Ilorin, Nigeria; 9 Department of Pharmaceutical and Medicinal Chemistry, University of Ilorin, P.M.B. 1515, Ilorin, Nigeria

**Keywords:** Drug discovery, Chemistry

## Abstract

Novel 1,1-diaryl vinyl-sulfones analogues of combretastatin CA-4 were synthesized via Suzuki–Miyaura coupling method and screened for in-vitro antiproliferative activity against four human cancer cell lines: MDA-MB 231(breast cancer), HeLa (cervical cancer), A549 (lung cancer), and IMR-32 (neuroblast cancer), along with a normal cell line HEK-293 (human embryonic kidney cell) by employing 3-[4,5-dimethylthiazol-2-yl]-2,5-diphenyltetrazolium bromide (MTT) assay. The compounds synthesised had better cytotoxicity against the A549 and IMR-32 cell lines compared to HeLa and MDA-MB-231 cell lines. The synthesized compounds also showed significant activity on MDA-MB-231 cancer cell line with IC_50_ of 9.85–23.94 µM, and on HeLa cancer cell line with IC_50_ of 8.39–11.70 µM relative to doxorubicin having IC_50_ values 0.89 and 1.68 µM respectively for MDA-MB-231 and HeLa cell lines. All the synthesized compounds were not toxic to the growth of normal cells, HEK-293. They appear to have a higher binding affinity for the target protein, tubulin, PDB ID = 5LYJ (beta chain), relative to the reference compounds, CA4 (− 7.1 kcal/mol) and doxorubicin (− 7.2 kcal/mol) except for 4E, 4M, 4N and 4O. The high binding affinity for beta-tubulin did not translate into enhanced cytotoxicity but the compounds (4G, 4I, 4J, 4M, 4N, and 4R, all having halogen substituents) that have a higher cell permeability (as predicted in-silico) demonstrated an optimum cytotoxicity against the tested cell lines in an almost uniform manner for all tested cell lines. The in-silico study provided insight into the role that cell permeability plays in enhancing the cytotoxicity of this class of compounds and as potential antiproliferative agents.

## Introduction

**Figure 1:**
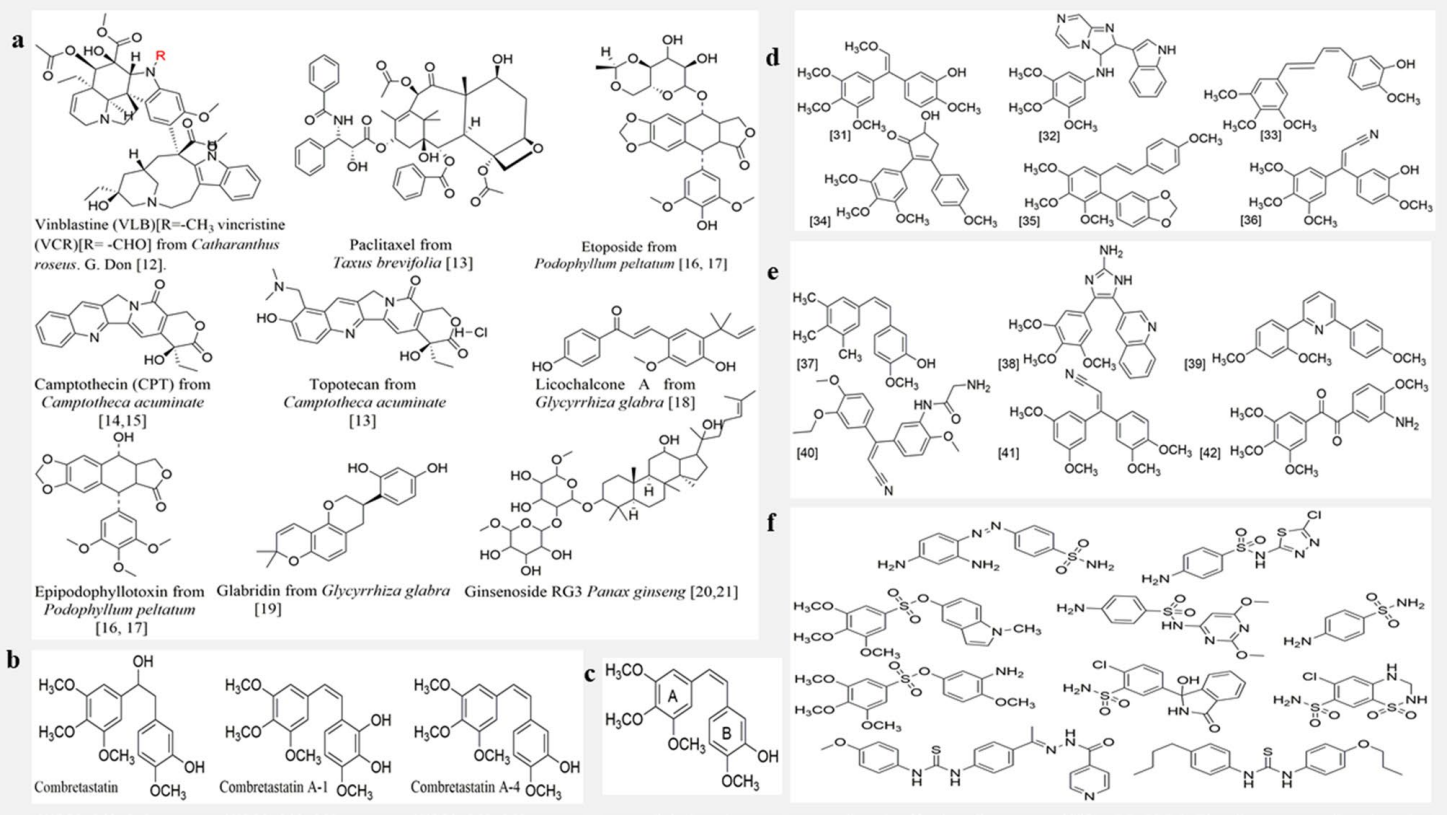
(**a**) Some anticancer therapeutic molecules of plant origin. (**b**) Structure of combretastatin, and combretastatin A-1 and A-4. (**c**) SAR of combretastatin analogues. (**d**) Variation of CA4 with respect to the Alkene group. (**e**) Variation of CA4 with respect to the Aryl group. (**f**) Some sulfonyl medicinal derivatives^30,31^.

Cancer has become a major health concern globally. One-fourth mortality rate in America is linked to cancer^1^. Its occurrence annually and globally is in millions, especially in developed countries, resulting to well over 5 million mortality rates annually^2^. This has necessitated the drive to find a lasting solution from both synthetic compounds^3–5^ and several other therapeutic medicinal agents inspired from plants or aquatic organisms^6–11^ to arrest the situation (Fig. 1a). Combretastatins were isolated, by Pettit et al. from the South African (S.A) *Combretum caffrum* (Eckl. & Zeyh.) Kuntze (Combretaceae) (Fig. 1b, c).

Combretastatin is a naturally occurring well known tubulin polymerization inhibitor. Tubulin polymerizes to form microtubules which furnish eukaryotic cells structure, thus playing a pivotal role in cancer development which therefore makes it an attractive target for the development of anticancer agents. A vast variety of drugs are able to bind to tubulin and modify its assembly properties, e.g. colchicine, vincristine, combretastatin, etc. The interference with microtubule dynamics can have the effect of stopping a cell-cycle and eventual apoptosis. Thus, a tubulin polymerization inhibitor is a potential anticancer agent^12–20^. Combretastatin A-4 (CA-4), one of the best known and most potent combretastatin, has been reported as a promising cytotoxic agent, which strongly prevents tubulin polymerization by binding to the colchicine site. Combretastatin CA-4, a simple stilbene [*cis* 1 (3,4,5-trimethoxyphenyl)-2-(30-hydroxyl-40-methoxy phenyl)-ethane] has been observed to vie for binding sites on tubulin in competition with colchicines^21,22^. CA-4 has therefore been considered as an appealing lead molecule for the improvement of antitumor drugs^23^. A number of CA-4 analogues such as CA-4P, AVE8062 (ombrabulin), ABT-751 (E7010), EPC2407 (Crolibulin), MPC-6827, OXi4503, T138067, BNC-105P etc. are in different phases of clinical trials^1,11,24^.

Several studies have revealed the structure activity relationship (SAR) of combretastatins^25,26^. Intrinsically, these isolates are stilbene like, having biphenyl rings separated via a C=C bond. Ring-A possess 3-OCH3 substituents at the 3,4,5-positions while in ring B, 1-OH group is at the C3 position and 1-OH group at the C4 position (Fig. 1c). To enhance the cytotoxicity of these isolates, a di-aromatic ring separated through a C=C bond along with a 3-OCH_3_ in one of the rings is essential (Fig. 1c)^27–29^.

Very many interesting analogues of CA-4 have been designed. In some analogues, the variation was carried out on the alkene functional group alone (Fig. 1d), while on other analogues, a complete transformation of the aryl ring is observed (Fig. 1e).

Worthy of note is that, in virtually every case, 3,4,5-trimethoxy aromatic ring (ring A), was kept constant, since it is considered very necessary for cytotoxicity of the compound as seen in Fig. 1a, b.

In this study, steps have been taken to replace the ethylene bridge in CA-4 with a sulfonyl (SO_2_R) group at the 2-carbon (C2) position, owing to its great medicinal properties. Sulphur based functional moieties (thioethers, sulfones, sulphonamides, penicillins) have been found in several natural products to have potent pharmacological activities (Fig. 1f). Many strategies have been used to develop several synthetic analogues of combretastatin A-4^32–37^, and in this study a new class of CA-4, 1,1-diaryl vinyl sulfones, have been designed, docked with beta-tubulin (colchicine site) to investigate its likely interaction as well as pharmacokinetic property predictions. The compounds were synthesized and tested in-vitro against four human cancer cell lines: MDA-MB 231(breast cancer), HeLa (cervical cancer), A549 (lung cancer), and IMR-32 (neuroblast cancer), along with a normal cell line HEK-293 (human embryonic kidney cell) by employing 3-[4,5-dimethylthiazol-2-yl]-2,5-diphenyltetrazolium bromide (MTT) assay to access their cytotoxicity and antiproliferative properties, and expressed as IC_50_ (µM) values.

## Results and discussion

### Chemistry

**Figure 2:**
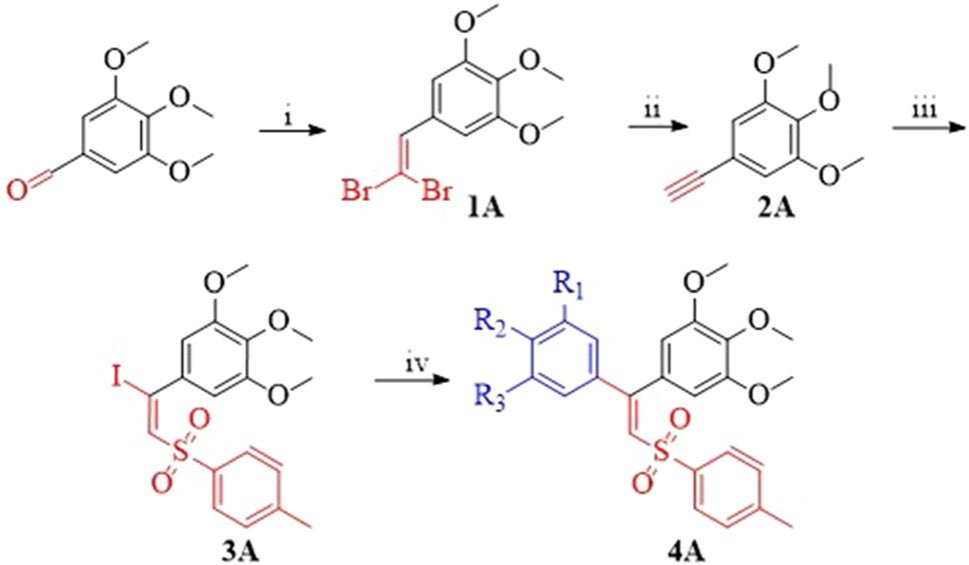
Complete Scheme of Reaction. (**i**) CBr_4_; PPh_3_, DCM, 0 °C, 2 h; (**ii**) 4.0 eqiv. DBU, CH_3_CN, r.t. 16 h; (**iii**) PhI(OAc)_2_, pTolSO_2_Na, KI, CH_3_CN, r.t. 1 h; (**iv**) Arylboronic acids, K_3_PO_4_, Pd(OAc)_2_, Cat., THF, r.t. 3 h.

The desired targeted analogues (**4a–v**) were synthesized from the readily available starting material of 3,4,5-trimethoxy benzaldehyde, prepared by Corey Fuchs olefination in the presence of carbon tetrabromide and triphenylphosphine in dichloromethane to afford the 5-(2,2-dibromovinyl)-1,2,3-trimethoxybenzene (**1A**) which in turn reacts with 1,8-diazabicyclo[5.4.0]undec-7-ene (DBU) in acetonitrie to give 5-ethynyl-1,2,3-trimethoxybenzene (**2A**). Further treatment of (**2A**) with diacetoiodobenzene (DIB), sodium-*p*-toluenesulfinate and potassium iodide in acetonitrile yields ((E)-5-(1-iodo-2-tosylvinyl)-1,2,3-trimethoxybenzene) (**3A**). ((E)-5-(1-iodo-2-tosylvinyl)-1,2,3-trimethoxybenzene) (**3A**) undergoes Suzuki Miyaura coupling with different substituted arylboronic acids in tetrahydrofuran (THF), in the sight of a base, potassium phosphate with palladium acetate as catalyst to give different combretastatin sulfonyl compounds (**4a–v**), as represented in Fig. 2.

### Synthesised compounds

#### Synthesis of 5-(2,2-dibromovinyl)-1,2,3-trimethoxybenzene [1A]

Light brown liquid, 3.786 g, 80% yield, mp: 51–53 °C; ^1^H NMR (400 MHz, CDCl_3_) δ 6.69 (1H, s), 6.55 (2H, s), 3.82 (6H, s), 3.78 (3H, s); ^13^CNMR (100 MHz, CDCl_3_) 153.45, 139.75, 136.80, 125.30, 108.70, 88.00, 60.91, 56.15.

#### Synthesis of 5-ethynyl-1,2,3-trimethoxybenzene [2A]

Yellow oil, 2.560 g 92% yield; mp: 68–70 °C; ^1^H NMR (400 MHz, CDCl_3_) δ 6.90 (2H, s), 3.82 (6H, s), 3.77 (3H, s), 2.83 (1H, s); ^13^CNMR (100 MHz, CDCl_3_) 152.88, 139.75, 116.30, 111.66, 84.00, 60.91, 56.15, 40.05.

#### Synthesis of (E)-5-(1-iodo-2-tosylvinyl)-1,2,3-trimethoxybenzene[3A]

Brown solid, 240 mg, 92% yield; mp: 169–171 °C; ^1^H NMR (500 MHz, CDCl_3_) δ 7.44 (2H, d, J = 8.4), 7.39 (1H, s), 7.17 (2H, d, J = 8.1), 6.38 (2H, s), 3.87 (3H, s), 3.78 (6H, s), 2.38 (3H, s); ^13^CNMR (100 MHz, CDCl_3_) 152.36, 144.40, 142.08, 139.07, 137.27, 134.48, 129.41, 128.01, 113.76, 105.37, 61.01, 56.09, 21.59; MS (ESI): *m/z* 475 [M + 1]^+^; HRMS (ESI): calcd for C_18_H_20_IO_5_S [M + 1]^+^ 475.00706, found 475.00934. IR (KBr) νmax: 3039, 2928, 2835, 1582, 1270, 1130, 994, 802 cm^−1^.

### Synthesised sulfonyl analogues of CA-4 (4A)

#### (Z)-1,2,3-trimethoxy-5-(1-phenyl-2-tosylvinyl)benzene (4a)

White solid; 52 mg, 85% yield; mp: 139–141 °C; ^1^H NMR (500 MHz, CDCl_3_) δ 7.44 (2H d, J = 8.192), 7.39 (1H, d, J = 7.091), 7.35 (2H, t, J = 7.703), 7.28 (1H, s), 7.13 (2H d, J = 8.069), 7.05 (1H s), 6.23 (1H,s), 3.90 (3H,s), 3.71 (6H,s), 2.36 (3H,s); ^13^CNMR (100 MHz,CDCl_3_) 154.56, 152.61, 143.53, 138.64, 138.43, 130.58, 130.46, 129.67, 129.05, 128.65, 128.21, 127.85, 107.36, 60.96, 55.99, 21.48. MS (ESI): *m/z* 425 [M + 1]^+^ ; HRMS (ESI) calcd for C_24_H_25_O_5_S [M + 1]^+^ 425.14172, found 425.14311; IR (KBr) νmax: 3035, 2934, 2837, 1583, 1309, 1140, 1083,760 cm^−1^.

(Session 1.0 *Synthesised Sulfonyl Analogues of CA-4* (4b–v). provides detailed report and characterization of compounds 4c–v, pls see the entire list as Supplementary documents Session 1.0).

A close look at the percentage yield of the synthesized compounds 4A–V, reveals interesting information. Compounds possessing electron donating groups gave a higher percentage yield while those possessing electron withdrawing groups had a lower percentage yield. This might be as a result of their respective electronic induction, which affect their yield.

### Cytotoxicity

**Figure 3:**
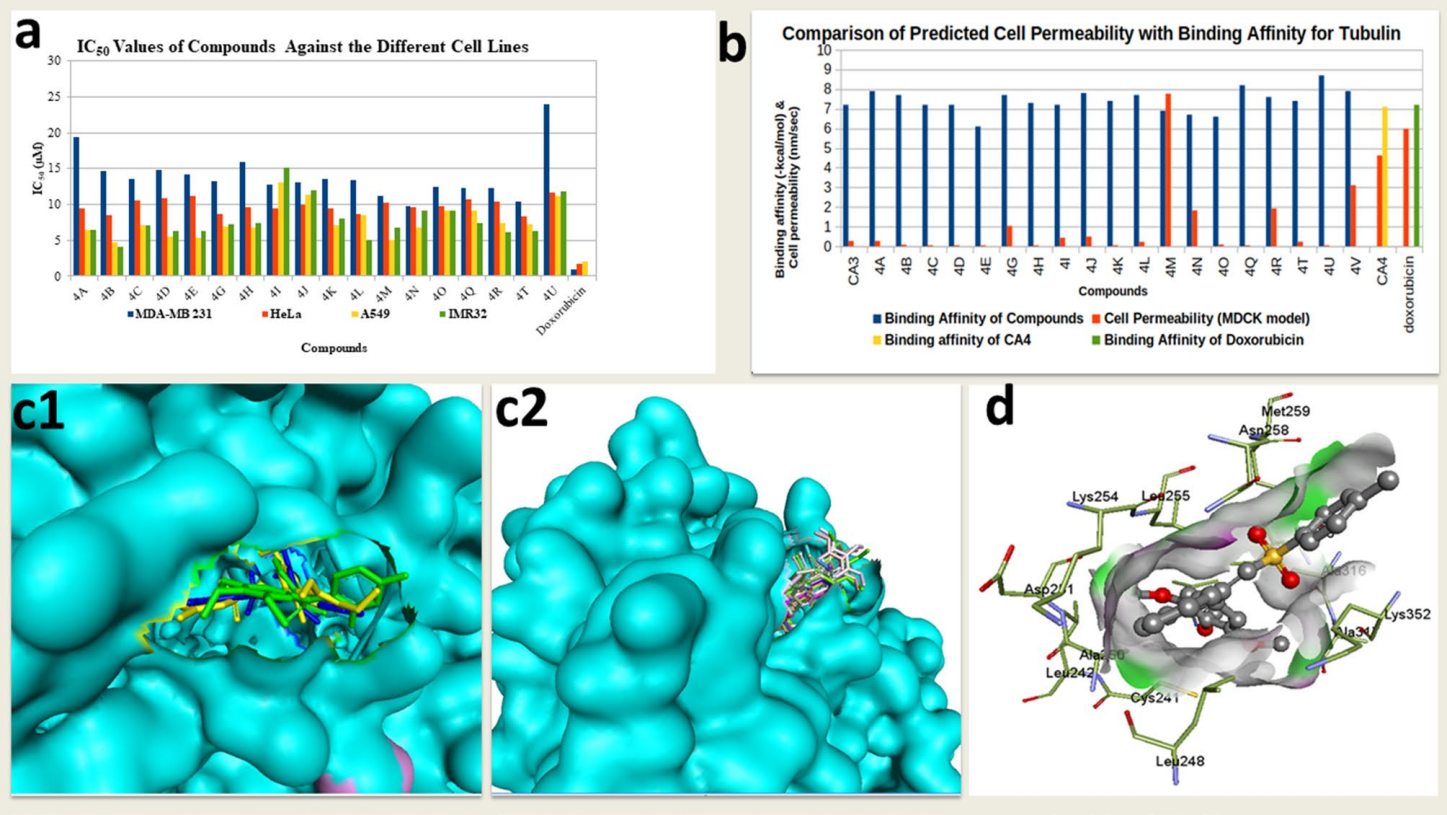
(**a**) Chart comparing the IC_50_ values of the compounds for different cell lines. (**b**) Chart comparing the predicted cell permeability with binding affinity for beta tubulin. (**c1**, **c2**) Surface rendition of the beta tubulin showing the colchicine site as a deep cavity showing two groups of compounds (A, I and K) and their preferred poses. The compounds in “c1” are positioned deeper in the cavity while those in “c2” (B, C, D, G, H, J, L, M, N, O, Q, R, T, U) are more pheripheral. (**d**) Compound 4I pose showing residues within 4 Å and the two polar contacts.

The in-vitro cytotoxicity assay reveals that all the synthesized compounds showed good to moderate antiproliferative activity against A549 and IMR-32 cancer cell lines (with IC_50_ values in the range of 4.10–15.10 µM) relative to the reference compound doxorubicin having IC_50_ values 0.23 and 2.06 µM respectively for IMR32 and A549 cell lines (Table 1; Fig. 3a). Essentially, the compounds appear to have better cytotoxicity against the A549 and IMR-32 cell lines compared to HeLa and MDA-MB-231 cell lines. The synthesized compounds also showed significant activity on MDA-MB-231 cancer cell line with IC_50_ values in the range of 9.85–23.94 µM, and on HeLa cancer cell line with IC_50_ values in the range of 8.39–11.70 µM relative to doxorubicin having IC_50_ values 0.89 and 1.68 µM respectively for MDA-MB-231 and HeLa cell lines (Table 1; Fig. 3a). Furthermore, all the synthesized compounds were evaluated for their cytotoxicity towards normal cell line, HEK-293. The results revealed that all the compounds were not toxic to the growth of normal cells, HEK-293.

**Table 1:** Antiproliferative activity (in-vitro) of the synthesised CA4 analogues against some cancer cell lines.

Compound	Structure					IC_50_ values against selected human cancer cell lines (µM)				
	R_1_	R_2_	R_3_	R_4_	R_5_	^a^MDA-MB 231	^b^HeLa	^c^A549	^d^IMR32	^e^HEK-293
**4A**	H	H	H	H	H	19.40 ± 1.72	9.40 ± 0.72	6.39 ± 0.68	6.40 ± 0.82	NA
**4B**	H	H	OCH_3_	H	H	14.59 ± 0.85	8.59 ± 0.58	4.68 ± 0.46	4.10 ± 0.96	NA
**4C**	H	H	OCH_3_	H	OCH_3_	13.56 ± 1.56	10.56 ± 0.56	7.04 ± 0.08	7.04 ± 0.83	NA
**4D**	H	OCH_3_	OCH_3_	H	H	14.87 ± 1.54	10.87 ± 0.54	5.52 ± 0.06	6.25 ± 0.03	NA
**4E**	H	OCH_3_	H	OCH_3_	H	14.12 ± 1.51	11.12 ± 0.51	5.32 ± 0.92	6.23 ± 0.09	NA
**4G**	H	H	F	H	H	13.25 ± 1.49	8.73 ± 0.49	6.92 ± 0.65	7.29 ± 0.46	NA
**4H**	H	H	CF_3_	H	H	15.92 ± 1.44	9.62 ± 0.44	6.84 ± 0.70	7.48 ± 0.13	NA
**4I**	F	H	F	H	H	12.72 ± 0.40	9.46 ± 0.40	13.08 ± 0.86	15.10 ± 0.16	NA
**4J**	H	H	F	F	H	13.14 ± 0.92	9.98 ± 0.42	11.30 ± 0.83	12.03 ± 0.02	NA
**4K**	F	H	H	OC_2_H_5_	H	13.56 ± 0.89	9.50 ± 0.49	7.02 ± 0.84	8.06 ± 0.09	NA
**4L**	H	H	F	Cl	H	13.38 ± 1.49	8.74 ± 0.49	8.54 ± 0.85	5.12 ± 0.36	NA
**4M**	H	H	Cl	H	H	11.14 ± 1.50	10.0.20 ± 1.80	5.12 ± 0.08	6.79 ± 0.44	NA
**4N**	H	Cl	Cl	H	H	9.85 ± 0.72	9.64 ± 0.96	6.79 ± 0.19	9.14 ± 0.84	NA
**4O**	H	H	CN	H	H	12.38 ± 1.67	9.83 ± 0.76	9.14 ± 0.91	9.08 ± 0.07	NA
**4Q**	H	H	H	CF_3_	F	12.25 ± 1.61	10.69 ± 0.74	9.08 ± 0.04	7.46 ± 0.61	NA
**4R**	H	CH_3_	H	H	F	12.22 ± 0.54	10.46 ± 1.03	7.46 ± 0.07	6.14 ± 0.03	NA
**4T**	H	H	CH_3_	F	H	10.44 ± 1.55	8.39 ± 0.99	7.22 ± 0.81	6.29 ± 0.07	NA
**4U**	H	H	C_6_H_5_	H	H	23.94 ± 1.56	11.70 ± 0.81	11.14 ± 0.78	11.77 ± 0.13	NA
Doxorubicin						0.89 ± 0.02	1.68 ± 0.24	2.06 ± 0.09	0.23 ± 0.76	NT

^a^MDA-MB 231(Breast cancer), ^b^HeLa (Cervical cancer), ^c^A549 (Lung cancer), ^d^IMR-32 (neuroblast), ^e^HEK-293 (Human embryonic kidney Normal cell line), NA (Not active), NT (Not tested). Data are the means of three experiments and are reported as mean ± standard error of the mean (SEM).

### In-silico Study

The experimental observations in the in-vitro cell line assays are in good agreement with the predictions from the in-silico study. The synthesized compounds appear to have a higher binding affinity for the target protein (tubulin–beta chain) relative to the reference compounds, CA4 (− 7.1 kcal/mol) and doxorubicin (− 7.2 kcal/mol) except for 4E, 4M, 4N and 4O (Table 2). Based on the higher affinity of the compounds for the beta-tubulin in the colchicine site (Fig. 3c(1&2)) preferred by combretastatin, it is expected that the compounds should have a higher inhibition of the test cell-lines relative to the reference compound (doxorubicin). However, it was observed that doxorubicin performed better in multiple folds in the cell line inhibition assays. The low inhibition of the test compounds relative to doxorubicin may be attributed to the low predicted cell permeability of the compounds (Table 2; Fig. 3b). The cell-permeability was predicted using the MDCK model on the preADMET server^38^. Predicted MDCK values less than 25 nm/s implies low permeability, value between 25 and 500 implies medium permeability while values higher than 500 implies high permeability. The predicted values for the compounds are less than 25 nm/s which is an indication that the compounds have low cell permeability. Most of the compounds having very low cell permeability, suggests that the compounds may not be sufficiently bioavailable at the site of action within the cell lines tested to cause a very strong inhibitory effect as observed in the cell line assays. The compounds with the highest binding affinity for beta-tubulin did not turn out to be the best cell line inhibitors, but the compounds 4G, 4I, 4J, 4M, 4N, and 4R all have relatively higher cell permeability (Table 2; Fig. 3b) and they appear to be the ones that perform better in the cell line assays (across board for all cell lines—having close IC_50_ values for the different cell lines tested)^39,40^. There were a few exceptions to the generally observed trend, such as compounds 4A and 4B which did not follow the observed general trend among the test compounds (Table 1; Fig. 3b). These two exceptions are among the compounds with the highest binding affinity for the beta-tubulin, have very low permeability but still have lower IC_50_ values for the A549 and IMR-32 cell lines relative to every other test compound while their IC_50_ values for MDA-MB 231, HeLa are significantly higher than those observed for A549 and IMR-32 cell lines and for many of the other test compounds (Table 1; Fig. 3b).

**Table 2:** Binding affinity from molecular docking and some predicted pharmacokinetic properties.

Compounds	Binding affinity (kcal/mol)	Cell permeability (MDCK model, nm/sec)	Plasma protein binding (PPB, %)	Human intestinal absorption (HIA, %)	Blood brain barrier (BBB, Conc. brain/Conc. blood)
**4A**	7.9	0.269739	86.762393	98.219366	2.09754
**4B**	7.7	0.0814893	95.503876	97.612504	2.82733
**4C**	7.2	0.0521959	94.898247	97.911744	2.46942
**4D**	7.2	0.0498051	92.727884	97.911744	2.45833
**4E**	6.1	0.0505377	92.116972	97.911744	2.51575
**4G**	7.7	1.03643	96.188881	97.496115	3.39249
**4H**	7.3	0.0434217	97.859757	97.521256	1.81215
**4I**	7.2	0.437982	98.969189	97.497328	3.39124
**4J**	7.8	0.492123	95.240245	97.497328	3.45247
**4K**	7.4	0.0546479	95.283704	97.582682	3.37422
**4L**	7.7	0.224686	94.619413	97.667523	3.26791
**4M**	6.9	7.76439	94.241021	97.663701	3.40251
**4N**	6.7	1.82734	92.906163	97.910731	2.77009
**4O**	6.6	0.0902112	97.110100	98.376564	2.14847
**4Q**	8.2	0.0486608	100.000000	97.523453	2.2711
**4R**	7.6	1.92628	96.665363	97.517038	3.16891
**4T**	7.4	0.23337	93.635318	97.517038	3.22547
**4U**	8.7	0.0466432	96.603119	97.922107	1.22368
**4V**	7.9	3.10782	85.486596	98.273346	2.28401
**CA4**	7.1	4.62504	94.650677	95.637206	0.171834
Doxorubicin	7.2	5.98801	45.102225	32.126871	0.0308154

It is however, notable that the only compounds that have significantly high cell permeability (relative to the other test compounds) as predicted, are those with the halogen substituents, 4G (R3 = F), 4M (R3 = Cl), 4N (R2 & R3 = Cl), 4R (R5 = F). It is worthy to note that the halogen groups confer extra lipophilicity which may facilitate cell-permeability. Compound 4T (R4 = F) also has cell-line inhibition profile similar to those of 4M, 4N, 4R and 4G but its predicted cell permeability is quite low compared to the four compounds—it however, has a halogen group like the others that follow the general trend.

The docking poses of the compounds revealed that the compounds occupy the colchicine binding site (Fig. 3c(1&2)), assuming different conformations and eleven out of seventeen docked compounds have polar interactions. The polar interactions were majorly with LYS254 and ASN350. Other residues involved in polar interactions are LYS352, ASN350, VAL315 and ASN349. Compounds 4E, 4H, 4I, 4K, 4N, 4O and 4U have polar interactions with their sulfonyl group oxygen with either sidechain –NH or backbone –CO of protein (Table 3). Only two (4I and 4N) of the compounds identified to have optimum cytotoxicity based on their pharmacokinetic profile, have polar contacts with the target protein (Table 3; Fig. 3d) and since these two are not the most potent in the MTT assay, it is believed that the polar contacts did not play a role in enhancing the cytotoxic property of the compounds.

**Table 3:** Residues within 4 Å and polar interactions of ligands with target protein.

Compound	Residues within 4 Å	Polar interactions
**4A**	VAL238, CYS241, LEU242, LEU248, ASN259, ALA250, ASP251, LYS254, LEU255, ASN258, MET259, THR314, VAL315, ALA316, ALA317, ILE318, ILE347, ASN349, ASN350, LYS352, THR353, ALA354, ILE378	
**4B**	VAL238, CYS241, GLN247, LEU248, ASN249, ALA250, ASP251, LYS254, LEU255, ASN258, MET259, THR314, VAL315, ALA316, ALA317, ILE347, PRO348, ASN349, ASN350, LYS352, THR353, ALA354, ILE378	LYS254; Sidechain NH3–OCH3 of ligand; 2.3 Å
**4C**	VAL238, CYS241, LEU242, GLN247, LEU248, ALA250, ASP251, LYS254, LEU255, VAL257, ASN258, MET259,, THR314, VAL315, ALA316, ALA317, ILE347, PRO348, ASN349, ASN350, LYS352, THR353, ALA354, ILE378	LYS254; Sidechain NH3–OCH3 of ligand; 2.2 Å LYS352; Sidechain NH3–OCH3 of ligand (second); 2.6 Å
**4D**	VAL238, CYS241, LEU242, LEU248, ASN249, ALA250, ASP251, LYS254, LEU255, ASN258, MET259, THR314, VAL315, ALA316, ALA317, ILE347, PRO348, ASN349, ASN350, LYS352, THR353, ALA354, ILE378	LYS254; Sidechain NH3–OCH3 of ligand; 2.1 Å
**4E**	CYS241, GLN247, LEU248, ASN249, ALA250, ASP251, LYS254, LEU255, VAL257, ASN258, MET259, VAL315, ALA316, ILE347, ASN349, LYS352	ASN350; Backbone CO–SO of Ligand; 3.3 Å
**4G**	VAL238, CYS241, LEU242, GLN247, LEU248, ALA250, ASP251, LYS254, LEU255, VAL257, ASN258, MET259, THR314, VAL315, ALA316, ALA317, ILE347, PRO348, ASN349, ASN350, ILE378	
**4H**	VAL238, CYS241, LEU242, GLN247, LEU248, ALA250, ASP251, LYS254, LEU255, VAL257, ASN258, MET259, THR314, VAL315, ALA316, ILE347, ASN349, ASN350, VAL351, LYS352, ILE378	LYS254; Sidechain NH3–OCH3 of ligand; 2.1 Å; 1.9 Å LYS254; Sidechain NH3–OCH3 of ligand; 2.9 Å ASN258; Sidechain NH–SO of ligand 3.1 Å
**4I**	ASN167, GLU200, TYR202, VAL238, CYS241, LEU242, GLN247, LEU248, ALA250, ASP251, LEU252, LYS254, LEU255, ASN258, MET259, VAL315, ALA316, ALA317, ILE318, ASN349, LYS352, THR353, ALA354, THR376, ILE378	LYS254; Bacbone CO–SO of Ligand 3.3 Å ASN258; Sidechain NH–SO of ligand 2.6 Å
**4J**	VAL238, CYS241, LEU242, LEU248, ASN249, ALA250, ASP251, LYS254, LEU255, ASN258, MET259, THR314, VAL315, ALA316, ALA317, ILE318, ILE347, PRO348, ASN349, ASN350, VAL351, LYS352, THR353, ALA354, ILE378	
**4K**	VAL238, CYS241, GLN247, LEU248, ASN249, ALA250, ASP251, LYS254, LEU255, ASN258, MET259, THR314, VAL315, ALA316, ALA317, ILE318, ILE347, PRO348, ASN349, ASN350, LYS352, THR353, ALA354, ILE378	VAL315; Backbone CO–SO of Ligand, 2.9 Å
**4L**	VAL238, CYS241, LEU242, LEU248, ALA250, ASP251, LYS254, LEU255, ASN258, MET259, THR314, VAL315, ALA316, ALA317, ILE347, PRO348, ASN349, ASN350, LYS352, THR353, ALA354, ILE378	
**4M**	VAL238, CYS241, LEU242, GLN247, LEU248, ALA250, ASP251, LYS254, LEU255, ASN258, MET259, THR314, VAL315, ALA316, ALA317, ILE347, PRO348, ASN349, ASN350, LYS352, THR353, ALA354, ILE378	
**4N**	VAL238, CYS241, LEU242, GLN247, LEU248, ALA250, ASP251, LYS254, LEU255, VAL257, ASN258, MET259, THR314, VAL315, ALA316, ALA317, ILE347, PRO348, ASN349, ASN350, LYS352, THR353, ALA354, ILE378	ASN258; Sidechain CO–SO of Ligand 3.2 Å
**4O**	VAL238, CYS241, LEU242, GLN247, LEU248, ALA250, ASP251, LYS254, LEU255, VAL257, ASN258, MET259, THR314, VAL315, ALA316, ALA317,	ASN258; Sidechain CO–SO of Ligand 3.2 Å LYS254; Sidechain NH3–OCH3 of ligand; 1.9 Å
**4Q**	VAL238, CYS241, LEU242, GLN247, LEU248, ALA250, ASP251, LYS254, LEU255, VAL257, ASN 258, MET259, THR314, VAL315, ALA316, ALA317, ILE347, ASN349, ASN350, VAL351, LYS352, THR353, ALA354, ILE378	
**4R**	VAL238, CYS241, LEU242, LEU248, ALA250, ASP251, LYS254, LEU255, ASN258, MET259, THR314, VAL315, ALA316, ILE347, PRO348, ASN349, ASN350, LYS352, THR353, ALA354, ILE378, ILE347, ASN349, ASN350, LYS352, ALA354, ILE378	
**4T**	VAL238, CYS241, LEU242, GLN247, LEU248, ASN249, ALA250, ASP251, LYS254, LEU255, VAL257, ASN258, MET259, THR314, VAL315, ALA316, ALA317, ILE347, PRO348, ASN349, ASN350, LYS352, THR353, ALA354, ILE378	
**4U**	VAL238, CYS241, GLN247, LEU248, ASN249, ALA250, LYS254, LEU255, VAL257, ASN258, MET259, THR314, VAL315, ALA316, MET325, ASN349, ASN350, LYS352	LYS352 Sidechain NH3–SO of Ligand; 2.6 Å ASN258 Sidechain NH2–SO of Ligand; 2.7 Å
Doxorubicin	VAL238, THR239, CYS241, LEU242, GLN247, LEU248, ASN249, ALA250, ASP251, LEU252, LYS254, LEU255, VAL257, ASN258, MET259, THR314, VAL315, ALA316, ALA317, ILE318, PRO348, ASN349, ASN350, VAL351, LYS352, ALA354, ILE378,	ASN349; Backbone CO–H_2_N-pyrimidine moiety of Doxorubicin LYS254; Sidechain NH3–OH-on acetyl moiety of Doxorubicin

Comparing the sulfonyl bridge analogues prepared in this study with other analogues prepared by other groups of researchers, such as the cyclopropyl amide analogues by Huan et al.^41^, pyridine-bridge analogues by Zheng et al.^42,43^ and biaryl aryl stilbenes by Kumar et al.^44^, it was observed that the pyridine-bridge analogues, appear to perform better than the sulfonyl bridge analogues in this study, based on a comparison of IC_50_ values on a relative scale—the pyridine-bridge analogues are active at a generally lower threshold of IC_50_ values compared to the compounds being reported here. However, the sulfonyl bridge analogues studied in this work appear to have better cytotoxicity compared to the cyclopropyl amide analogues and biaryl aryl stilbenes against the tested cell lines (HeLa and A549) comparing the reported IC_50_ values for the compounds synthesized and tested by Huan et al.^41^, and Kumar et al.^44^, with those reported for the compounds in this study—the cyclopropyl amides and biaryl aryl stilbenes appear to be active generally at a higher threshold of IC_50_ values relative to the compounds being reported here which means that the compounds being reported here are more active than the cyclopropyl amides and biaryl aryl stilbenes reported by Huan et al.^41^ and Zheng et al.^42^ respectively. It therefore, implies that the introduction of the sulphonyl group as a bridge between the stilbeneoid aromatic rings may moderately improve the antiproliferative activity of the combretastatin analogues relative to many other synthetic analogues.

In addition, it was envisioned that variation of the substituents on ring-**B** provided remarkable SAR information about these compound CA-4 congeners. The introduction of substituents on ring B were exemplified by preparing analogues possessing electron donating groups (4B–4E**)**, electron withdrawing groups (4G–4J and 4I–4Q) as well as both electron donating and withdrawing groups (4K, 4R, and 4K). However, compound possessing biphenyl group (4U) on ring **B** shows poor cytotoxicity compared to the simple phenyl group (4A) thereby increase in the number of phenyl moieties, activity was declined. Compound 4B displayed prominent potent antiproliferative activity against lung cancer (A549), human neuroblastoma (IMR32), and cervical cancer (HeLa). So, the presence of 4-methoxy phenyl on ring B is essential for cytotoxicity. In contrast, order of activity of electron donating substituents on ring B was 4-methoxy (4B) > 3,5-dimethoxy (4E) > 3,4-dimethoxy (4D) > 4,6-dimethoxy (4C). It was observed that compounds with electron withdrawing substituents on the B-ring such as halogens, like fluoro, difluoro, chloro, dichloro, and trifluoro methyl on ring B which also enhance the lipophilicity of the compounds must have improved the cell permeability making the compounds more bioavailable^39,40^ within the cell to carry out its predicted biological function of inhibiting tubulin polymerization and this includes the compound with the nitrile group, noting that it has also been established that the nitrile group is sufficiently lipophilic^38^ when it replaces an halogen attached to an aromatic ring and can enhance cell permeability as postulated in this study.

## Conclusion

A series of 20 compounds of the analogues of CA-4 using Suzuki Miyaura coupling method were successfully synthesized and fully characterized using different spectroscopic techniques. The compounds possessing electron donating substituents gave higher percentage yields than those possessing electron withdrawing substituent. This is due to their positive electronic induction effect. All the synthesized compounds evaluated for their anticancer activity against four cancer cell lines**,** MDA-MB 231 (breast cancer), HeLa (cervical cancer), A549 (lung cancer), and IMR-32 (neuroblast cancer) showed moderate to good activity and non were cytotoxic towards normal healthy cell lines (HEK 293) except compound 4V which showed some level of cytotoxicity. The observed results from both in-silico and in-vitro study shows that the compounds are a source of potential lead compounds that can be optimized further especially by improving their cell permeability. Based on the observation of improved cell-lines inhibition profile of test compounds by halogen substitution, the compounds may be optimized further by incorporating more halogen substituents at different positions (and perhaps on the other aromatic ring) and investigate if there would be an improvement in the anticancer profile of the optimized compounds.

## Methodology

### Experimental section

#### General information^3–5^

Melting points were obtained in open capillary tubes with a 1101D Mel-Temp^®^ Digital Melting Point Apparatus from Cole-Palmer Limited and are unuttered. The infrared spectra were documented on Thermo Nicolet Nexus 670 Spectrometer with 4 cm^−1^ resolution using KBr beam splitter and wave numbers of maximal absorption peaks were presented n cm^−1^. NMR spectra were recorded on Bruker 300, 400, 500 and 600 MHz NMR Spectrometers, ^13^C-NMR spectra were documented at 75, 100, 125, 150 MHz. The proton resonances are annotated as chemical shifts δ parts per million (ppm) relative to tetramethylsilane (δ 0.0) using the residual solvent signal as an internal standard or tetramethylsilane itself: chloroform-d (δ 7.26, singlet), multiplicity (s, singlet; d, doublet; t, triplet; q, quartet; m, multiplet; br, broad), coupling constant J, in hertz (Hz), and the number of protons for a given resonance indicated by nH. The chemical shifts of ^13^C NMR are reported in ppm, relative to the central line of the triplet at ẟ 77.0 ppm for CDCl_3_. ESI spectra were recorded on Micro mass Quattro LC using ESI+ software with a capillary voltage of 3.98 kV and an ESI mode positive ion trap detector. High-resolution mass spectra (HRMS) were recorded on a QSTAR XL hybrid MS–MS mass spectrometer.

All reactions were carried out in flame-dried round-bottom flasks, fitted with rubber septa under nitrogen gas. Removal of solvent was done using a Heidolph Hei-VAP Value Digital G3: 560-01302-00 rotary evaporator attached to a vacuum pump (∼ 3 mmHg). Analytical thin-layer chromatography (TLC) was performed using aluminum foil UVF_254_ precoated silica gel flexible plates. Column chromatography was conducted using silica gel (60–120 mesh and 100–200 mesh) packed in glass columns. Technical grade solvents which include: n-hexane, methanol, ethyl acetate, acetone, chloroform, dichloromethane, and ethanol were distilled before use. All chemicals used were supplied by Sigma-Aldrich Company, USA. These include: Phenylacetylene, Iodosobenzene diacetate, p-Toluenesulfinic acid sodium salt, Boronic Acids, 1,2,3-trimethoxy benzaldehyde, Sodium thiosulphate, Potassium iodide, etc.

A panel of four cancer cell lines used for testing the in-vitro cytotoxicity include: MDA-MB-231 derived from human breast adenocarcinoma cells (ATCC No HTB-26), HeLa derived from human cervical cancer cells (ATCC No. CCL-2), A549 derived from human alveolar adenocarcinoma epithelial cells (ATCC No. CCL-185) and IMR-32 derived from human neuroblastoma cell line (ATCC No CCL-127), with a normal cell line HEK-293 (human embryonic kidney cell). These cancer cell lines were obtained from the American Type Culture Collection (ATCC), Manassas, VA, USA, and stored before use.

### Synthesis of 5-(2,2-dibromovinyl)-1,2,3-trimethoxybenzene^45^

**Figure 4:**
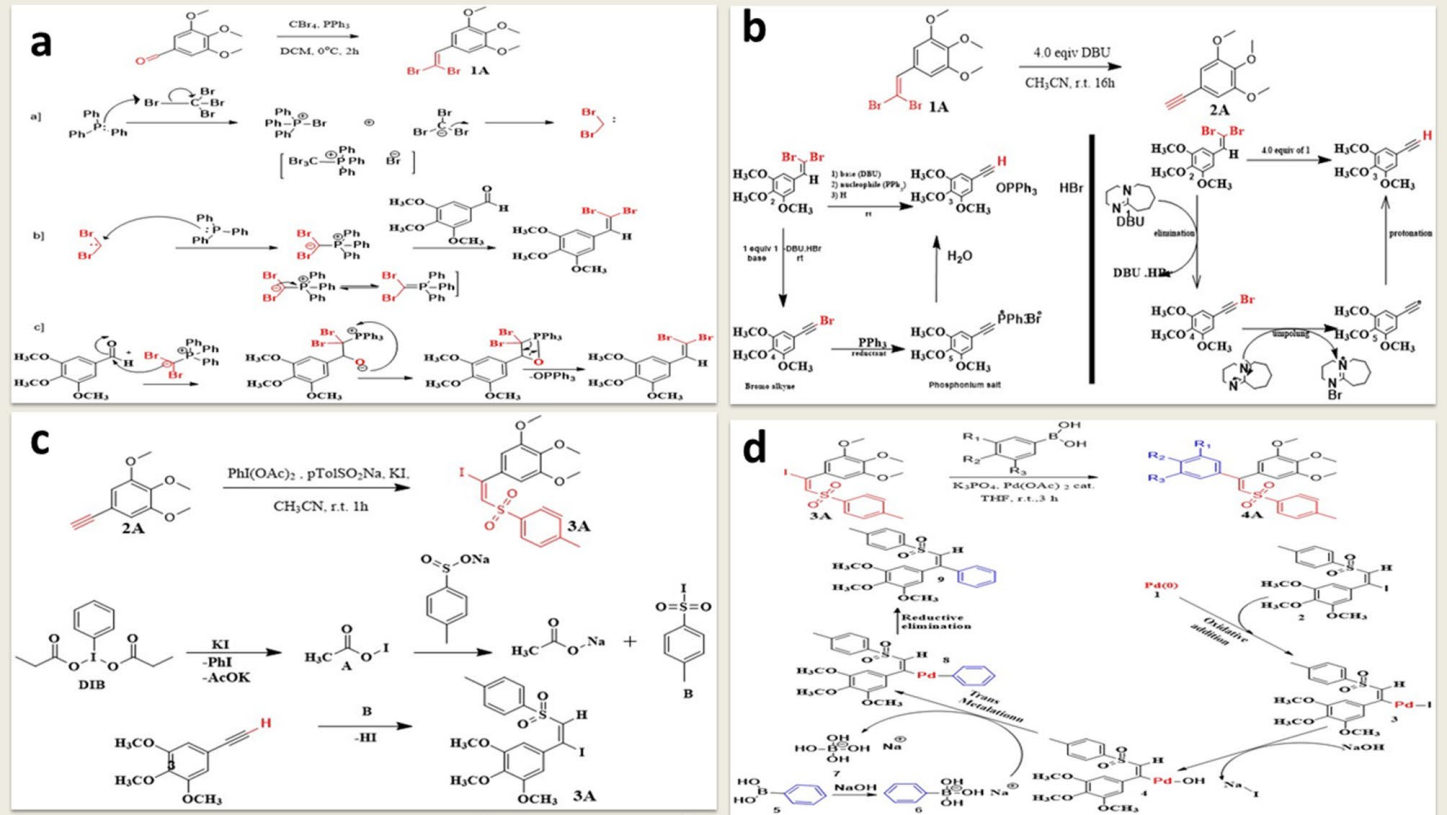
(**a**) Proposed mechanistic route for the synthesis of 5-(2,2-dibromovinyl)-1,2,3-trimethoxybenzene^45^. (**b**) Proposed mechanistic route for the synthesis of 5-ethynyl-1,2,3-trimethoxybenzene^46^. (**c**) Plausible mechanistic rationalization for the synthesis of (e)-5-(1-iodo-2-tosylvinyl)-1,2,3-trimethoxybenzene^47^. (**d**) plausible mechanistic rationalization to the synthesis of 1,1-diarylvinyl sulfones ca4 analogues (4a–v)^48,49^.

To a stirred solution of 3,4,5-trimethoxybenzaldehyde (2 g, 10.2 mmol) and carbon tetrabromide (6.766 g, 20.4 mmol) in anhydrous dichloromethane was added triphenylphosphine (10.702 g, 40.8 mmol) at 0 °C under inert atmosphere. After completion of reaction (monitored by TLC), reaction mixture was quenched with water and extracted with dichloromethane. The organic layer was dried over anhydrous Na_2_SO_4_ and evaporated to dryness. The crude was purified on SiO_2_ (60–120 mesh) column using Hexanes/EtOAc (95:5) as eluents to afford the analytically pure **1A** (Fig. 4a)**.**

### Synthesis of 5-ethynyl-1,2,3-trimethoxybenzene^46^

To a stirred solution of dibromoalkene 2A (3.786 g, 10.76 mmol) in anhydrous CH_3_CN was added DBU (6.552 g, 43.10 mmol) drop wise over a period of 10 min at ambient temperature (25–30 °C). The reaction mixture was allowed to stir at ambient temperature for 16 h. After completion of reaction (monitored by TLC), reaction mixture was cooled at 15 °C and quenched by drop wise addition of 5 N aqueous HCl (10 mL) over a period of 15 min then continued stirring for 5 min. The reaction mixture was extracted with EtOAc/hexane (1:1, 2 × 10 mL); organic layers were washed with water (10 mL). The organic layers were dried over anhydrous Na_2_SO_4_, solvent was evaporated under reduced pressure, and resulting residues were dried in high vacuum to afford the analytically pure 2A (Fig. 4b) as yellow oil (2.560 g, 92% yield).

### Synthesis of (E)-5-(1-iodo-2-tosylvinyl)-1,2,3-trimethoxybenzene^47^

PhI(OAc)_2_ (502.73 mg, 1.56 mmol) was added to a suspension of the alkyne 3A (200 mg, 10.40 mmol), sodium arenesulfinate (741.61 mg, 41.60 mmol), and KI (172.73 mg, 10.40 mmol) in CH_3_CN, and the reaction mixture was vigorously stirred at room temperature for 1 h. Upon completion of the reaction, the reaction mixture was quenched by the addition of a saturated aqueous solution of Na_2_S_2_O_3_ (5 mL) and basified with a saturated aqueous solution of NaHCO_3_ (5 mL). Further stirring was followed by extraction with EtOAc (3 × 15 mL). The combined organic extract was washed with H_2_O (15 mL) and brine (15 mL), dried (Na_2_SO_4_), filtered, and concentrated. The residue was purified by column chromatography to afford 3A (Fig. 4c).

### General method for the synthesis of 1,1-diarylvinyl sulfones CA4 analogues (4a–u)^48–50^

To a stirred mixture of (E)-5-(1-iodo-2-tosylvinyl)-1,2,3-trimethoxybenzene 4A (100 mg, 0.211 mmol, 1 eq), different aryl boronic acids (30.64 mg, 0.253 mmol, 1.2 eq) and Na_2_CO_3_ (44.73 mg, 0.422 mmol, 2 eq) in water and dimethylformalmide (1:2), with palladium acetate (Pd(OAc)_ 2_) catalyst (0.0006 mmol, 0.005 eq) was added and reaction was stirred at room temperature under nitrogen for 3 h. The residue was purified by chromatography (EtOAc/hexanes) to afford the corresponding coupled product 4A-V (Fig. 4d).

### Antiproliferative assay

The cytotoxicity of synthesized compounds 4A–V were determined in triplicates by 3-[4,5-dimethylthiazol-2-yl]-2,5-diphenyltetrazolium bromide (MTT) assay^51^^,^^52^. The pale yellow coloured tetrazolium salt (MTT) reduced to a dark blue water-insoluble formazan by metabolically active cells and the product was measured quantitatively after solubility in DMSO. The absorbance of the soluble formazan is directly proportional to the number of viable cells. A panel of four cancer cell lines were used for testing the in-vitro cytotoxicity. These cell lines were grown in Dulbecco’s modified Eagle’s medium (DMEM) containing non-essential amino acids and 10% FBS. All the cells were maintained under humidified conditions of 5% CO_2_ atmosphere at 37 °C in a CO_2_ incubator (Model Galaxy 170S, Eppendorf, USA). The 96-well micro titre plates were incubated for 24 h prior to addition of the experimental compounds. Cells were treated with vehicle alone (DMSO) or compounds (drugs (1 µg/mL) were dissolved in 100 µL DMSO previously) at different concentrations (0.1, 1, 10 and 25 µM) of test compounds for 48 h. The assay was completed with the addition of MTT (5%, 10µL) and incubated for 60 min at 37 °C. The supernatant was aspirated and plates were air dried and the MTT-formazan crystals were dissolved in 100 µL of DMSO.

The optical density (O.D) was measured at 570 nm using TECAN multimode reader (Infinite^®^ M200Pro, Tecan, Switzerland). The percent cell viability of each treated well of 96 well plate was calculated based on test wells relative to control wells. Doxorubicin was used as positive controls for comparison purpose and 1% DMSO as a vehicle control. The cytotoxicity of test compounds was expressed in terms of IC_50_ value, which is defined as a concentration of compound that produced 50% reduction absorbance relative to control, and the absorbance at 570 nm wavelength was recorded^51^.

### Molecular docking and in-silico pharmacokinetics prediction

The coordinates of the tubulin (protein) were downloaded from the protein databank (PDB code = 5LYJ). The beta tubulin portion (B chain) that consists of the colchicine site (Fig. 3c(1&2)), that is the target for combretastatins, was extracted from the whole protein and stripped of other associated ligands, water of crystallization and ions (Ca^2+^ and Mg^2+^) and saved in a pdb format. This extracted chain B was processed further using the AutoDock program (MGL Tools 1.5.6). Gasteiger charges were added and the search space area was set by centering the grid box at the colchicine site, capturing all the residues within 4 Å of the native combretastatin within the protein. The grid box parameters (x, y, z) in Angstrom units are (22, 20, 24) for size and (16.061, 66.352, 38.609) for centre and the processed protein chain was saved as a pdbqt file. The native doxorubicin ligand was redocked with the protein and the rmsd of the native ligand and redocked native ligand was estimated (0.7029 Å) in order to validate the docking protocol. The 3D structures of the synthesized compounds were prepared using chem3D and their energies were minimized with MM2 Force Field of Chem3D application interface and the ligands were saved as pdb files. The command line version of the *obabel* program, installed on Ubuntu LTS operating system, was used to convert the pdb files into pdbqt files. The pdbqt files for the protein and prepared ligands were used for the docking calculation with AutoDock Vina. The resulting binding poses and interactions were visualized and processed using the Pymol program. The binding energy of the compounds as well as interacting residues are presented in Table 2. The compounds were submitted to the online server, preADMET, for in-silico pharmacokinetic property prediction to obtain estimates for cell permeability, plasma protein binding, blood brain barrier penetration and Intestinal absorption.

## Supplementary Information

Supplementary Information.
