# Supplementary material for: Synthesis and characterization of novel combretastatin analogues of 1,1-diaryl vinyl sulfones, with antiproliferative potential via in-silico and in-vitro studies
Source: Sci Rep. 2022 Feb 3;12:1901. doi: 10.1038/s41598-022-05958-6 (PMC8814031; doi:10.1038/s41598-022-05958-6)
Supplement: Supplementary file 1 — Supplementary Information. [file 41598_2022_5958_MOESM1_ESM.pdf]

SUPPLIMENTARY INFORMATION

SYNTHESIS AND CHARACTERIZATION OF NOVEL COMBRETASTATIN ANALOGUES OF 1, 1-DIARYL VINYL SULFONES, WITH ANTIPROLIFERATIVE POTENTIAL VIA *IN-SILICO* AND *IN-VITRO* STUDIES

Godshelp O. Egharevba[1-4*], Ahmed Kamal[2,5], Omotayo O. Dosumu[6], Sunitha Routhu[2], Olatomide A. Fadare[7], Stephene O. Oguntoye[8], Stanislaus N. Nginga[9], Abimbola P. Oluyori[1-4]

[1]Industrial Chemistry Programme, Department of Physical Sciences, College of Pure and Applied Sciences, Landmark University, Omu-Aran, Kwara State, Nigeria.
[2]Medicinal Chemistry and Pharmacology Division, CSIR-Indian Institute of Chemical Technology, Hyderabad 500 007, India.
[3]Landmark University SDG 3 (Good Health and Well-being)
[4]Landmark University SDG 12 (Responsible Consumption and Production)
[5]Jamia Hamdard, New Delhi 110062, India.
[6]Department of Industrial Chemistry, University of Ilorin, P.M.B. 1515, Ilorin, Nigeria.
[7]Department of Chemistry, Obafemi Awolowo University, Ile-Ife, Osun State, Nigeria.
[8]Department of Chemistry, University of Ilorin, P.M.B. 1515, Ilorin, Nigeria.
[9]Department of Pharmaceutical and Medicinal Chemistry, University of Ilorin, P.M.B. 1515, Ilorin-Nigeria.

*Corresponding author:
Email: godhelpeghas@gmail.com; egharevba.godshelp@lmu.edu.ng
Tel: +2348067448464

Session 1.0. ***Synthesised Sulfonyl Analogues of 4* (4b-v).** provides detailed report and characterization of compounds 4c to v, as indicated in the body of the manuscript from *2.2.2 – 2.2.20.*

2.2.2 (Z)-1,2,3-trimethoxy-5-(1-(4-methoxyphenyl)-2-tosylvinyl)benzene (4b)

White solid; 87 mg, 80% yield; mp: 124-126 °C; ^{1}H NMR (500 MHz, $CDCl_3$) δ 7.42 (2H d, 7.947), 7.22 (2H d, J=8.861), 7.12 (2H d, J=7.947), 6.98 (1H s), 6.85 (2H d, J=8.681), 6.21 (2H, s), 3.90 (3H s), 3.81 (3H s), 3.70 (6H s), 2.35 (3H s); ^{13}CNMR (100 MHz,$CDCl_3$) 161.59, 154.21, 152.56,143.33, 138.72, 138.22, 130.83, 129.78, 128.99, 127.79, 127.39, 114.03, 107.33, 60.96, 55.95, 55.45, 21.46; MS (ESI): m/z 455 $[M+1]^+$; HRMS (ESI) calcd for $C_{25}H_{27}O_6S$ $[M+1]^+$ 455.15229, found 455.15419; IR (KBr) νmax: 3056, 2939, 2839, 1583, 1305, 1246, 1127, 838 cm^{-1}

2.2.3 (E)-5-(1-(2,4-dimethoxyphenyl)-2-tosylvinyl)-1,2,3-trimethoxybenzene (4c)

Yellow solid; 53 mg, 75% yield; mp: 107-109 °C; ^{1}H NMR (400 MHz, $CDCl_3$) δ 7.44 (2H d, J=8.069), 7.29 (1H s), 7.12 (2H d,J=7.947), 6.83 (1H d, J=8.681), 6.47 (1H s), 6.37-6.35 (1H dd, J=8.681), 6.22 (1H s), 3.88 (3H s), 3.80 (6H s), 3.70 (6H s), 2.35 (3H s); ^{13}CNMR (100 MHz,$CDCl_3$) 162.14, 159.26, 152.29, 151.10, 143.01, 139.16, 137.91, 133.23, 131.22, 130.90, 129.70, 128.89, 128.62, 127.68, 126.46, 120.27, 107.33, 104.51, 99.02, 60.92, 55.93, 55.69, 55.46, 30.95, 21.45; MS (ESI): m/z 485 $[M+1]^+$; HRMS (ESI): calcd for $C_{26}H_{29}O_7S$ $[M+1]^+$ 485.16285, found 485.16490; IR (KBr) νmax: 3261, 2933, 2842, 1584, 1414, 1297, 1130, 839 cm^{-1}

2.2.4 ***(Z)-5-(1-(3,4-dimethoxyphenyl)-2-tosylvinyl)-1,2,3-trimethoxybenzene (4d)***
Yellow solid; 70 mg, 92% yield; mp: 173-175 °C; ^{1}H NMR (400 MHz, $CDCl_3$) δ 7.42 (2H d, J=8.314), 7.12 (2H d, J=8.069), 7.01 (1H s), 6.84 (1H s), 6.77 (2H s), 6.20 (2H s), 3.91 (3H s), 3.88 (3H s), 3.64 (3H s), 3.70 (6H s), 2,36 (3H s); ^{13}CNMR (100 MHz,$CDCl_3$) 154.35, 152.52, 151.31, 148.97, 143.36, 138.64, 138.24, 131.01, 128.99, 127.81, 127.67, 122.34, 110.72, 110.37, 107.38, 60.97, 56.13, 55.96, 21.46 ; MS (ESI): m/z 485$[M+1]^+$; HRMS (ESI) calcd for $C_{26}H_{29}O_7S$ $[M+1]^+$ 485.16285, found 485.16490; IR (KBr) νmax: 3026, 2938, 2844, 1583, 1278, 1134, 1079, 839 cm^{-1}

2.2.5 ***(Z)-5-(1-(3,5-dimethoxyphenyl)-2-tosylvinyl)-1,2,3-trimethoxybenzene (4e)***
White solid; 93 mg, 77% yield; mp: 163-165 °C; ^{1}H NMR (500 MHz, $CDCl_3$) δ 7.42 (2H d, J=8.240), 7.12 (2H d, J=7.934), 7.03 (1H s), 6.48 (1H t, J=2.289), 6.38 (2H d, J= 2.289), 6,21 (2H s), 3.89 (3H s), 3.75 (6H s), 3.71 (6H s), 2.36 (3H s) ^{13}CNMR (100 MHz,$CDCl_3$) 160.77, 154.44, 152.54, 143.55, 140.72, 138.31, 130.37, 130.09, 129.04, 127.84, 107.29, 106.66, 101.98, 60.95, 55.97, 55.55, 21.48; MS (ESI): m/z 485 $[M+1]^+$; HRMS (ESI) calcd for $C_{26}H_{29}O_7S$ $[M+1]^+$ 485.16285, found 485.16490; IR (KBr) νmax: 3028, 2940, 2838, 1588, 1284, 1080, 841, 571 cm^{-1}

2.2.6 ***4-methylphenyl (3,4,5-trimethoxyphenyl)ethynyl sulfone (4f)***
Yellow solid; 60 mg, 37% yield; mp: 121-123 °C; ^{1}H NMR (400 MHz, $CDCl_3$) δ 7.97 (2H d, J=8.3), 7.41 (2H d, J=8.2), 6.75 (2H s), 3.87-3.83 (9H s), 2.47(3H s); ^{13}CNMR (100 MHz,$CDCl_3$) 153.22, 145.39, 141.58, 138.96, 130.03, 127.52, 112.49, 110.07, 93.43, 84.78, 61.07, 56.33, 21.78; MS (ESI): m/z 347 $[M+1]^+$; HRMS (ESI) calcd for $C_{18}H_{19}O_5S$ $[M+1]^+$ 347.09477, found 347.09634; IR (KBr) νmax: 3090, 2937, 2841, 1378, 1127, 828, 757 cm^{-1}

2.2.7 ***(Z)-5-(1-(4-fluorophenyl)-2-tosylvinyl)-1,2,3-trimethoxybenzene (4g)***
White solid; 43mg, 63% yield; mp: 141-143 °C; ^{1}H NMR (500 MHz, $CDCl_3$) δ 7.44 (2H s, J=8.1), 7.27-7.24 (2H m), 7.13 (2H d, J=8.1), 7.04 - 6.99 (3H m), 6.22 (2H s), 3.90 (3H s), 3.71 (6H s), 2.36 (3H s); ^{13}CNMR (100 MHz,$CDCl_3$) 164.10 (d, J=252.49 Hz)153.39, 152.68, 143.61, 138.39 (d, J=19.98 Hz), 134.80, 132.46, 130.90, 130.39, 130.22 (d, J=9.08 Hz), 129.09, 127.83, 115.76 (d, J= 21.80 Hz), 107.29, 60.95, 55.99, 21.47; MS (ESI): m/z 443$[M+1]^+$; HRMmS (ESI) calcd for $C_{24}H_{24}FO_5S$ $[M+1]^+$ 443.13230, found 443.13405; IR (KBr) νmax: 3007, 2925, 2853, 1582, 1298, 1126, 875 cm^{-1}

2.2.8 ***(Z)-1,2,3-trimethoxy-5-(2-tosyl-1-(4-(trifluoromethyl)phenyl)vinyl)benzene (4h)***
White solid; 82 mg, 66% yield; mp: 125-127 °C; ^{1}H NMR (500 MHz, $CDCl_3$) δ7.60 (2H d, J=8.2), 7.46 (2H d, J= 8.1), 7.39 (2H d, J= 8.1), 7.15 (2H d, J= 7.9), 7.07 (1H s), 6.23 (2H s), 3.91 (3H s), 3.72 (3H s), 2.37 (3H s); ^{13}CNMR (100 MHz,$CDCl_3$) 152.90, 152.81, 143.91, 138.60, 137.94, 131.65, 128.89 (q), 125.65, 107.21, 60.99, 56.03, 21.52; MS (ESI): m/z 493$[M+1]^+$; HRMS (ESI) calcd for $C_{25}H_{24}F_3O_5S$ $[M+1]^+$ 493.12911, found 493.13163; IR (KBr) νmax: 3052, 2927, 2849, 1581, 1313, 1124,1067, 834 cm^{-1}

2.2.9 ***(E)-5-(1-(2,4-difluorophenyl)-2-tosylvinyl)-1,2,3-trimethoxybenzene (4i)***
White solid; 88 mg, 60% yield; mp: 113-115 °C; ^{1}H NMR (500 MHz, $CDCl_3$) δ7.48 (2H d, J= 8.2), 7.15(2H d, J=7.9), 7.03-6.97 (2H m), 6.88 -6.80 (2H m) , 6.27 (2H s), 3.89 (3H s), 3.73(6H s), 2.37 (3H s); ^{13}CNMR (100 MHz,$CDCl_3$) 152.66, 147.74, 143.74, 138.61, 138.16, 133.72, 133.66, 130.57, 129.16, 127.81, 123.23, 111.85, 111.68, 107.17, 105.10, 104.89, 104.68, 60.96, 56.04, 21.51; MS (ESI): m/z 461$[M+1]^+$; HRMS (ESI) calcd for $C_{24}H_{23}F_2O_5S$ $[M+1]^+$ 461.12288, found 461.12483; IR (KBr) νmax: 3049, 2993, 2934, 1594, 1128, 845 cm^{-1}

2.2.10 (E)-5-(1-(3,4-difluorophenyl)-2-tosylvinyl)-1,2,3-trimethoxybenzene (4j)
White solid; 76 mg, 63% yield; mp: 140-142 °C; ^{1}H NMR (500 MHz, $CDCl_3$) δ7.44 (2H d, J=8.1), 7.15 (3H d, J=8.1), 7.10 (2H m), 6.98 (1H s), 6.21 (2H s), 3.91 (3H s), 3.72 (6H s), 2.37 (3H s); ^{13}CNMR (100 MHz,$CDCl_3$) 152.80, 152.73, 152.22, 143.84, 138.62, 138.01, 135.76, 130.56, 129.76, 129.17, 127.89,124.56, 117.66, 117.52, 117.45, 117.31, 107.19, 60.99, 56.01, 21.51; MS (ESI): m/z 461[M+1]$^+$; HRMS (ESI) calcd for $C_{24}H_{23}F_2O_5S$ [M+1]$^+$ 461.12288, found 461.12513; IR (KBr) νmax: 3031, 2924, 2847, 1516, 1288, 1128, 836 cm^{-1}

2.2.11 (E)-5-(1-(5-ethoxy-2-fluorophenyl)-2-tosylvinyl)-1,2,3-trimethoxybenzene (4k)
Yellow solid; 108 mg, 70% yield; mp: 125-127 °C; ^{1}H NMR (500 MHz, $CDCl_3$) δ7.47 (2H d, J=8.2), 7.14 (2H d, J=8.1), 7.04-6.98 (2H m), 6.84 - 6.81 (1H dt, J=8.96), 6.50 (1H dd, J=6.2), 6.29 (2H s), 3.88 (5H m), 3.73(6H s), 2.36 (3H s), 1.34 (3H t, J=6.9); ^{13}CNMR (100 MHz,$CDCl_3$) 155.58, 154.79, 152.56, 148.70, 143.63, 138.54, 138.25, 133.91, 133.84, 130.66, 129.10, 127.80, 117.24, 116.53, 107.20, 64.18, 60.93, 56.04, 21.50, 14.70; MS (ESI): m/z 487 [M+1]$^+$; HRMS (ESI) calcd for $C_{26}H_{28}FO_6S$ [M+1]$^+$ 487.15851, found 487.16028; IR (KBr) νmax: 3074, 2939, 2826, 1584, 1255, 1126, 831 cm^{-1}

2.2.12 (E)-5-(1-(3-chloro-4-fluorophenyl)-2-tosylvinyl)-1,2,3-trimethoxybenzene (4l)
Yellow solid; 66 mg, 44% yield; mp: 123-125 °C; ^{1}H NMR (400 MHz, $CDCl_3$) δ7.44 (2H d, J= 8.3). 7.30 (1H dd, J= 6.9), 7.15 - 7.12 (4H m), 6.97 (1H s), 6.21 (2H s), 3.91 (3H s), 3.72 (6H s), 2.37 (3H s); ^{13}CNMR (100 MHz,$CDCl_3$) 152.81, 152.14, 143.84, 138.02, 130.61, 130.41, 129.77, 129.17, 128.21, 127.88, 116.77, 107.25, 60.97, 56.03, 21.50; MS (ESI): m/z 477 [M+1]$^+$; HRMS (ESI) calcd for $C_{24}H_{23}ClFO_5S$ [M+1]$^+$ 477.09333, found 477.09542; IR (KBr) νmax: 3038, 2958, 2837, 1583, 1284, 1123, 815 cm^{-1}

2.2.13 (Z)-5-(1-(4-chlorophenyl)-2-tosylvinyl)-1,2,3-trimethoxybenzene (4m)
White solid; 61 mg, 42% yield; mp: 96-98 °C; ^{1}H NMR (500 MHz, $CDCl_3$) δ7.44 (2H d, J= 7.93), 7.31 (2H d, J=8.24), 7.20 (2H d, J= 8.4), 7.14 (2H d, J= 7.78), 7.01 (1H s), 6.21 (2H s), 3.90 (3H s), 3.72 (6H s), 2.36 (3H s); ^{13}CNMR (100 MHz,$CDCl_3$) 153.25, 152.72, 143.70, 138.52, 138.22, 137.16, 130.15, 129.99, 129. 48, 129.12, 128.91, 127.86, 107.29, 60.97, 56.01,21.49; MS (ESI): m/z 459 [M+1]$^+$; HRMS (ESI) calcd for $C_{24}H_{24}ClO_5S$ [M+1]$^+$ 459.10275, found 459.10496; IR (KBr) νmax: 3035, 2924, 2849, 1584, 1287, 1141, 1127, 852 cm^{-1}

2.2.14 (E)-5-(1-(3,4-dichlorophenyl)-2-tosylvinyl)-1,2,3-trimethoxybenzene (4n)
Yellow solid; 65 mg, 42% yield; mp: 151-153 °C; ^{1}H NMR (400MHz, $CDCl_3$) δ7.44 (2H m), 7.41 (1H s), 7.34 (1H d, J=2.08), 7.15 (2H d, J= 8.07), 7.00 (1H dd, J=2.08), 6.21 (2H s), 3.91 (3H s), 3.73 (6H s), 2.37 (3H s); ^{13}CNMR (126 MHz,$CDCl_3$) 152.83, 152.03, 143.89, 138.76, 137.94, 134.75,133.13, 131.04, 130.62, 129.64, 129.19, 127.89, 127.37, 107.23, 60.97, 56.04, 21.51; MS (ESI): m/z 493[M+1]$^+$; HRMS (ESI) calcd for $C_{24}H_{23}Cl_2O_5S$ [M+1]$^+$ 493.06378, found 493.06600; IR (KBr) νmax: 3064, 2940, 2837, 2360, 1579, 1127, 1028, 829 cm^{-1}

2.2.15 (Z)-4-(2-tosyl-1-(3,4,5-trimethoxyphenyl)vinyl)benzonitrile (4o)
Yellow solid; 72 mg, 51% yield; mp: 165-167 °C; ^{1}H NMR (400MHz, $CDCl_3$) δ7.63 (2H d, J =8.31), 7.46 (2H d, J= 8.07), 7.38(2H d, J= 8.31), 7.16 (2H d, J= 7.95), 7.06 (1H s), 6.22 (2H s), 3.91 (3H s), 3.72 (6H s), 2.38 (3H s); ^{13}CNMR (126 MHz,$CDCl_3$) 152.90, 152.26, 144.06, 143.08, 138.77, 137.79, 132.38, 129.43, 129.24, 128.77, 127.93, 118.09, 113.83, 107.22, 60.97, 56.04, 21.51; MS (ESI): m/z 450 [M+1]$^+$; HRMS (ESI) calcd for $C_{25}H_{24}NO_5S$ [M+1]$^+$ 450.13697, found 450.13898; IR (KBr) νmax: 3050, 2942, 2839, 2227, 1580, 1240, 842 cm^{-1}

2.2.16 ***(E)-5-(1-(2-fluoro-3-(trifluoromethyl)phenyl)-2-tosylvinyl)-1,2,3-trimethoxybenzene (4q)***

White solid; 71 mg, 44% yield; mp: 128-130 °C; ^{1}H NMR (500MHz, $CDCl_3$) δ7.63 (1H t, J= 6.41), 7.61 (2H d, J= 8.24), 7.24- 7.16 (4H m), 7.04 (1H s), 6.31 (2H s), 3.89(3H s), 3.74 (6H s), 2.38 (3H s); ^{13}CNMR (100 MHz,$CDCl_3$) 152.79, 146.77, 143.99, 143.99, 137.91, 135.33, 130.16, 129.26, 127.86, 124.15, 107.17, 60.95, 56.11, 21.53; MS (ESI): m/z 511[M+1]$^+$; HRMS (ESI) calcd for $C_{25}H_{23}F_4O_5S$ [M+1]$^+$ 511.11968, found 511.12183; IR (KBr) νmax: 3042, 2950, 2837, 1584, 1332, 1133, 851 cm^{-1}

2.2.17 ***(E)-5-(1-(2-fluoro-5-methylphenyl)-2-tosylvinyl)-1,2,3-trimethoxybenzene (4r)***

White solid; 55mg, 38% yield; mp: 118-120 °C; ^{1}H NMR (400MHz, $CDCl_3$) δ7.48 (2H d, J= 7.95), 7.14 (3H d, J= 7.82), 7.01-6.94 (2H m), 6.79 (1H d), 6.29 (2H s), 3.89 (3H s), 3.73(6H s), 2.36 (3H s), 2.24 (3H s) ; ^{13}CNMR (100 MHz,$CDCl_3$) 152.54, 149.03,143.57, 138.34, 133.81, 133.61, 132.03,131.63, 129.09, 127.80, 116.21,115.98, 107.20, 60.94, 56.05, 21.50, 20.60; MS (ESI): m/z 457 [M+1]$^+$; HRMS (ESI) calcd for $C_{25}H_{26}FO_5S$ [M+1]$^+$ 457.14795, found 457.14966; IR (KBr) νmax: 3025, 2940,1583, 1503, 1415, 1216, 1128, 822 cm^{-1}

2.2.18 ***(E)-5-(1-(3-fluoro-4-methylphenyl)-2-tosylvinyl)-1,2,3-trimethoxybenzene (4t)***

Light yellow solid;76 mg, 53% yield; mp: 124-126 °C; ^{1}H NMR (400MHz, $CDCl_3$) δ7.43 (2H d, J= 8.31), 7.13 (3H m), 7.01 (1H s), 6.98 (1H dd, J= 1.71), 6.89 (1H dd, J= 1.71), 6.21 (2H s), 3.90 (3H s), 3.71 (6H s), 2.36 (3H s), 2.28 (3H s); ^{13}CNMR (100 MHz,$CDCl_3$) 153.28, 152.67, 143.62, 138.45, 138,29, 131.57, 130.15, 129.74, 129.09, 127.86, 123.51, 114.92, 114.68, 107.27, 60.97, 55.99, 21.48, 14.54; MS (ESI): m/z 457 [M+1]$^+$; HRMS (ESI) calcd for $C_{25}H_{26}FO_5S$ [M+1]$^+$ 457.14795, found 457.15009; IR (KBr) νmax: 3030, 2926, 2360, 1579, 1287, 1123, 814 cm^{-1}

2.2.19 ***(Z)-4-(2-tosyl-1-(3,4,5-trimethoxyphenyl)vinyl)-1,1'-biphenyl (4u)***

White solid; 94 mg, 59% yield; mp: 132-134 °C; ^{1}H NMR (400MHz, $CDCl_3$) δ7.57 (4H s), 7.44 (4H s), 7.35(3H s), 7.13 (3H m), 6.26 (2H s), 3.92 (3H s), 3.73 (6H s), 2.37 (3H s); ^{13}CNMR (100 MHz, $CDCl_3$) ; MS (ESI): m/z 501 [M+1]$^+$; HRMS (ESI) calcd for $C_{30}H_{29}O_5S$ [M+1]$^+$ 501.17302, found 501.17500; IR (KBr) νmax: 3058, 2925, 1580, 1305, 1130, 823 cm^{-1}

2.2.20 ***4-methylphenyl (3,4,5-trimethoxyphenyl)ethynyl sulfone (4v)***

Yellow solid; 84 mg, 67% yield; mp: 119-121 °C; ^{1}H NMR (400 MHz, $CDCl_3$) δ 7.97 (2H d, J=8.3), 7.41 (2H d, J=8.2), 6.75 (2H s), 3.87-3.83 (9H s), 2.47(3H s); ^{13}CNMR (100 MHz,$CDCl_3$) 153.22, 145.38, 141.59, 138.97, 130.02, 127.53, 112.50, 110.07, 93.43, 84.78, 61.08, 56.33, 21.79; MS (ESI): m/z 347 [M+1]$^+$; HRMS (ESI) calcd for $C_{18}H_{19}O_5S$ [M+1]$^+$ 347.09477, found 347.09608; IR (KBr) νmax: 3090, 2937, 2841, 1573, 1298, 803 cm^{-1}

APPENDICES I: ^{1}H NMR SPECTRA OF SYNTHESIZED COMPOUNDS

1. $C_{11}H_{12}Br_2O_3$; 5-(2,2-dibromovinyl)-1,2,3-trimethoxybenzene

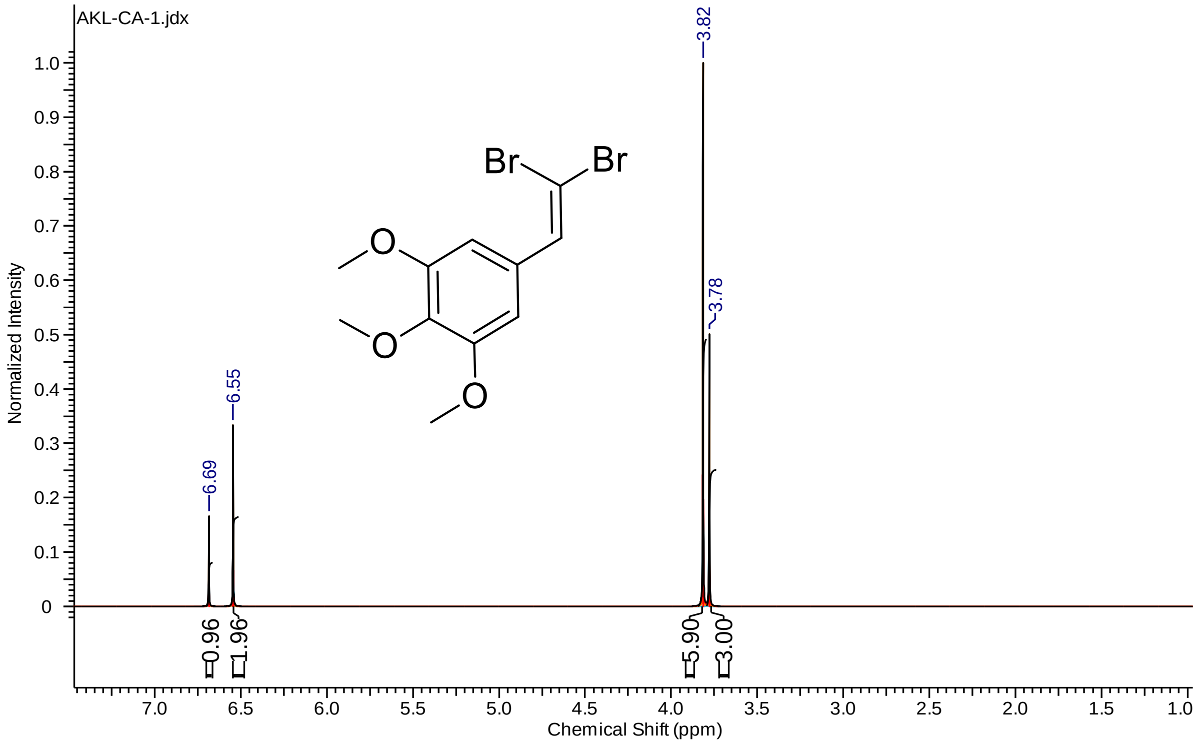

2. $C_{11}H_{12}O_3$, 5-ethynyl-1,2,3-trimethoxybenzene

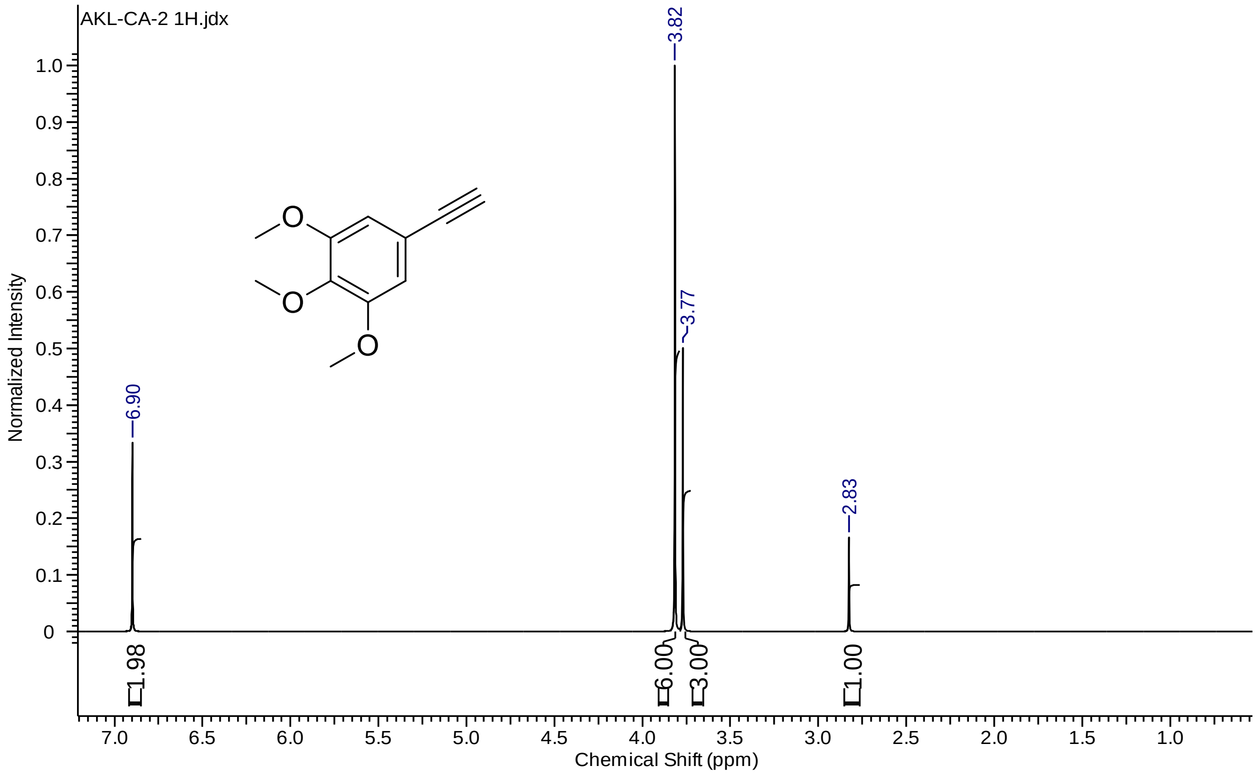

3. $C_{18}H_{19}IO_5S$, (E)-5-(1-iodo-2-tosylvinyl)-1,2,3-trimethoxybenzene

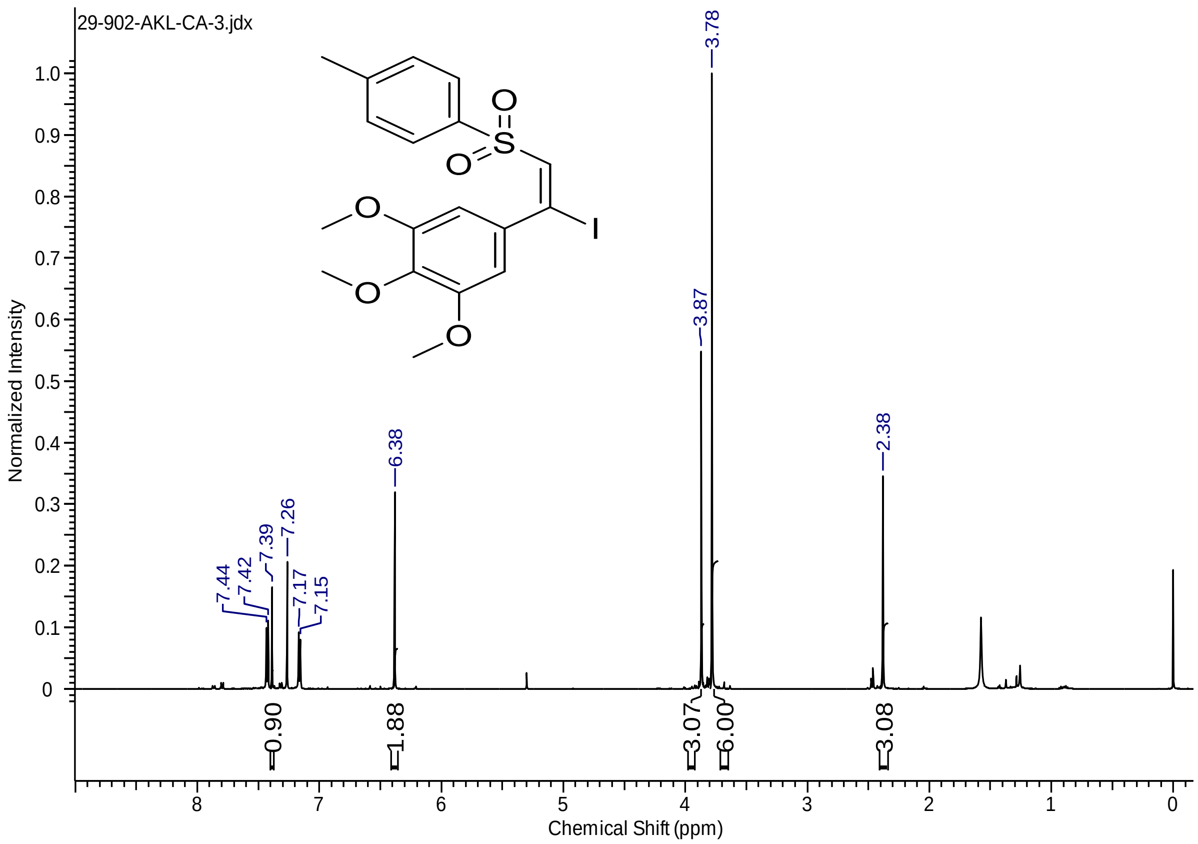

4. 4A, $C_{24}H_{24}O_5S$, (Z)-1,2,3-trimethoxy-5-(1-phenyl-2-tosylvinyl)benzene

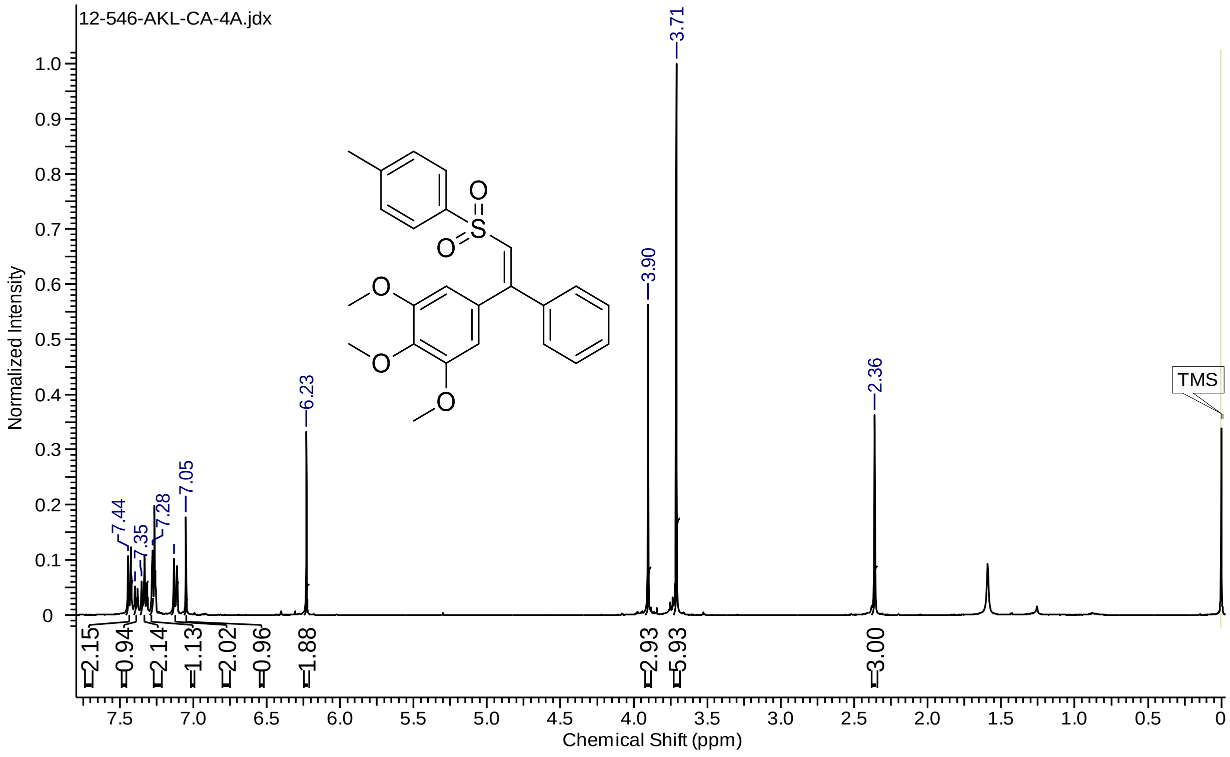

5. 4B, (Z)-1,2,3-trimethoxy-5-(1-(4-methoxyphenyl)-2-tosylvinyl)benzene

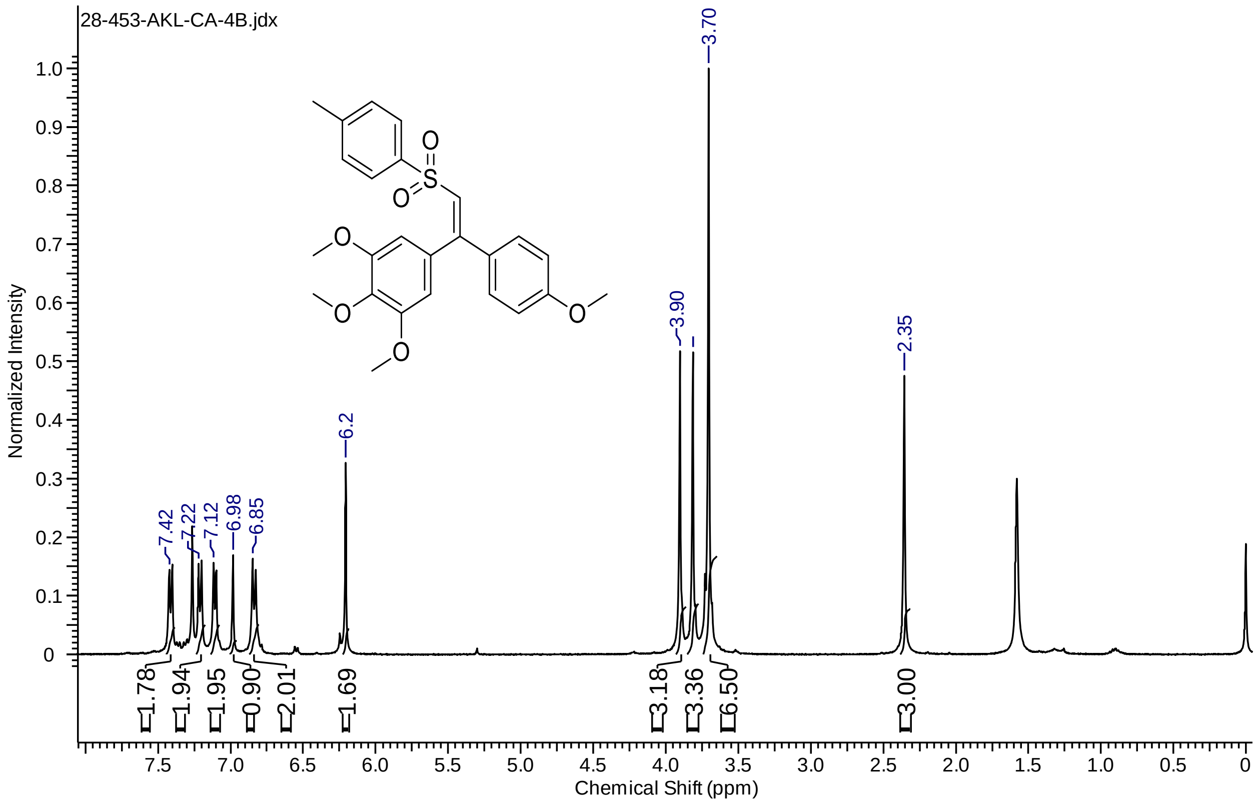

6. 4C, $C_{26}H_{28}O_7S$, (E)-5-(1-(2,4-dimethoxyphenyl)-2-tosylvinyl)-1,2,3-trimethoxybenzene

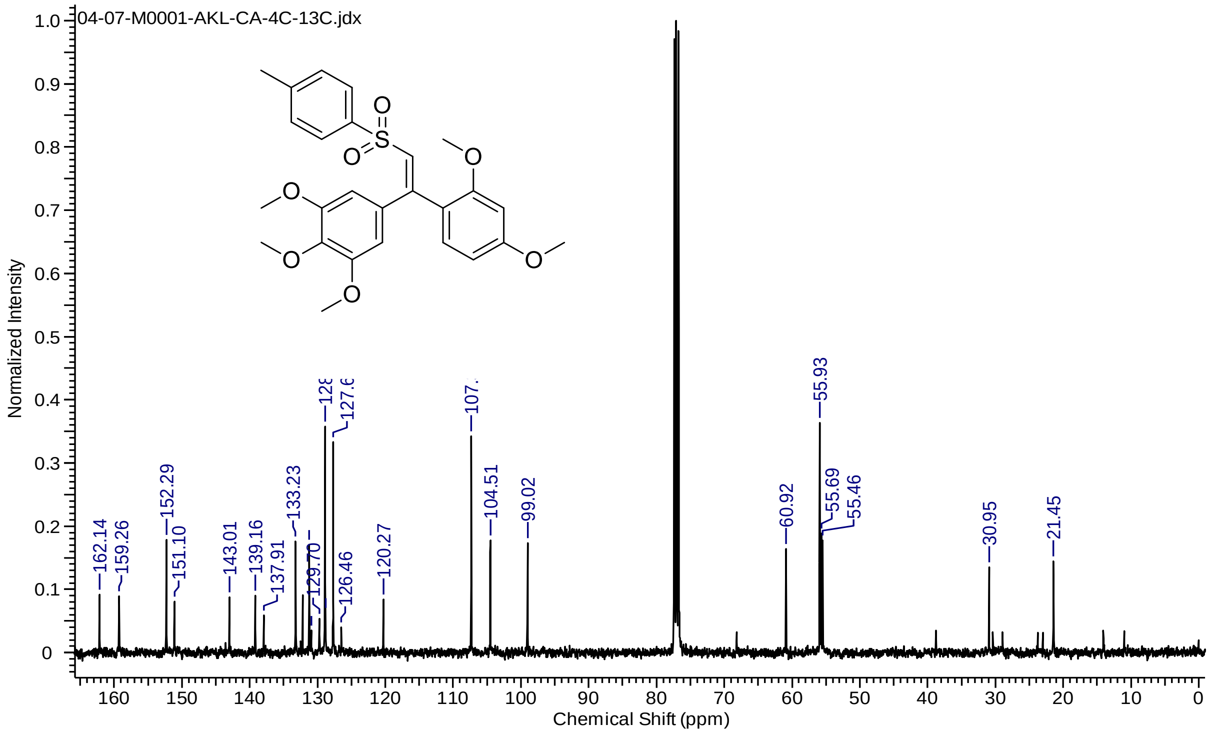

7. 4D, $C_{26}H_{28}O_7S$, (Z)-5-(1-(3,4-dimethoxyphenyl)-2-tosylvinyl)-1,2,3-trimethoxybenzene

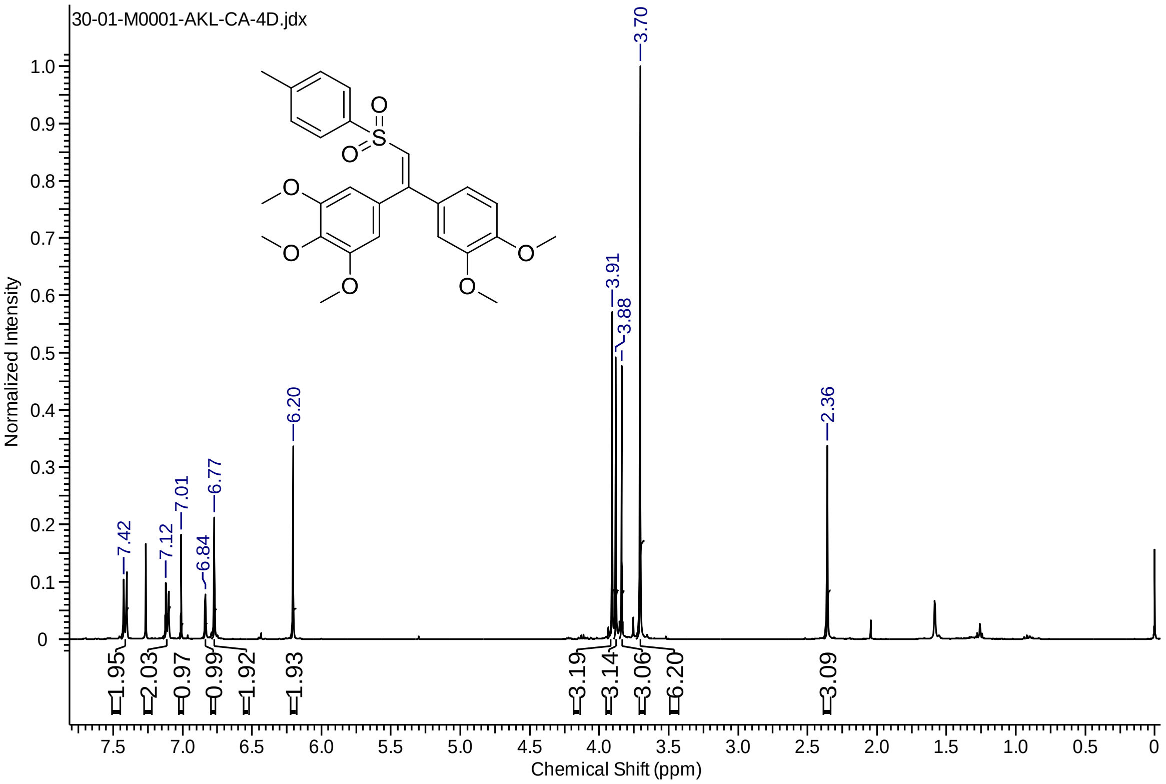

8. 4E, $C_{26}H_{28}O_7S$, (Z)-5-(1-(3,5-dimethoxyphenyl)-2-tosylvinyl)-1,2,3-trimethoxybenzene

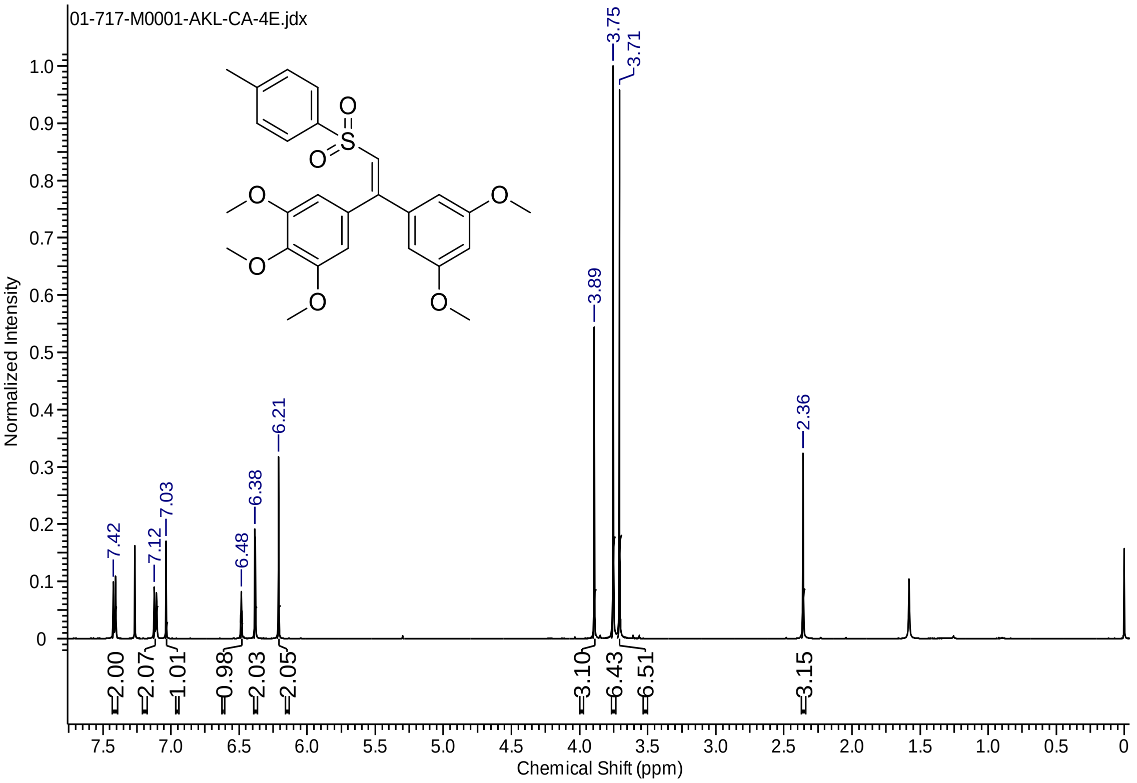

9. 4F, $C_{18}H_{18}O_5S$, 4-methylphenyl (3,4,5-trimethoxyphenyl)ethynyl sulfone

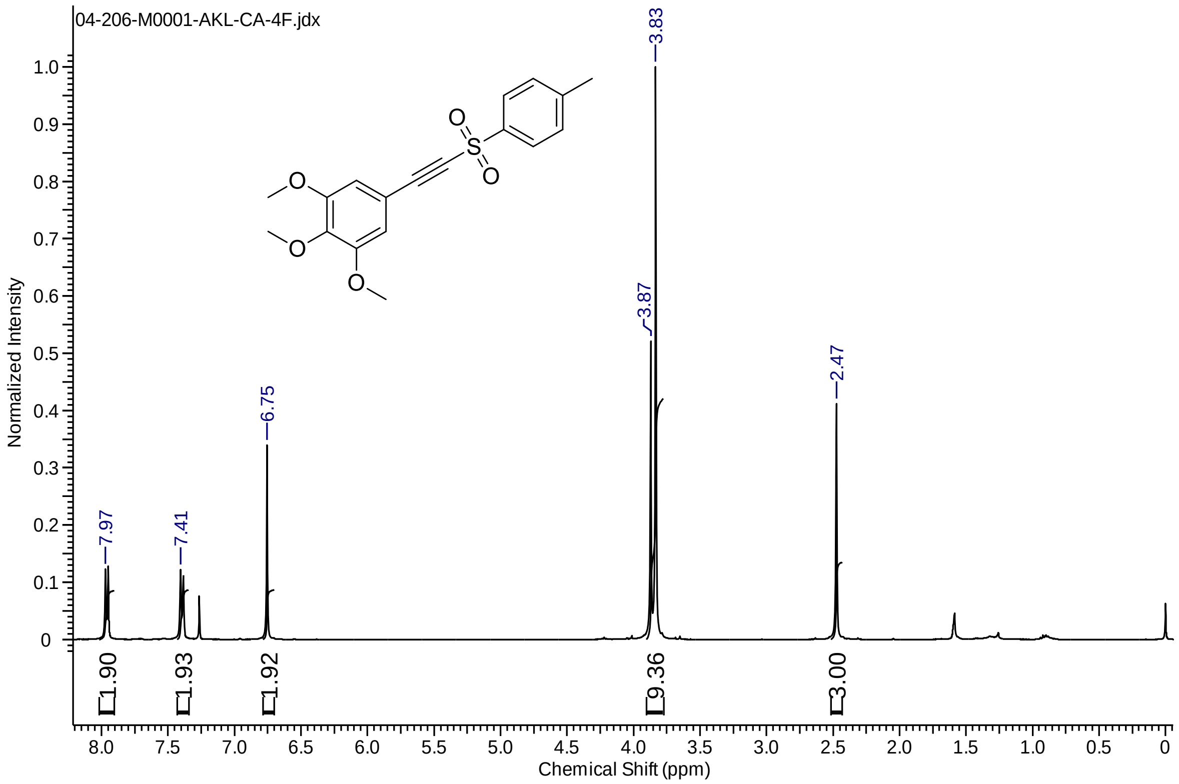

10. 4G, $C_{24}H_{23}FO_5S$, (Z)-5-(1-(4-fluorophenyl)-2-tosylvinyl)-1,2,3-trimethoxybenzene

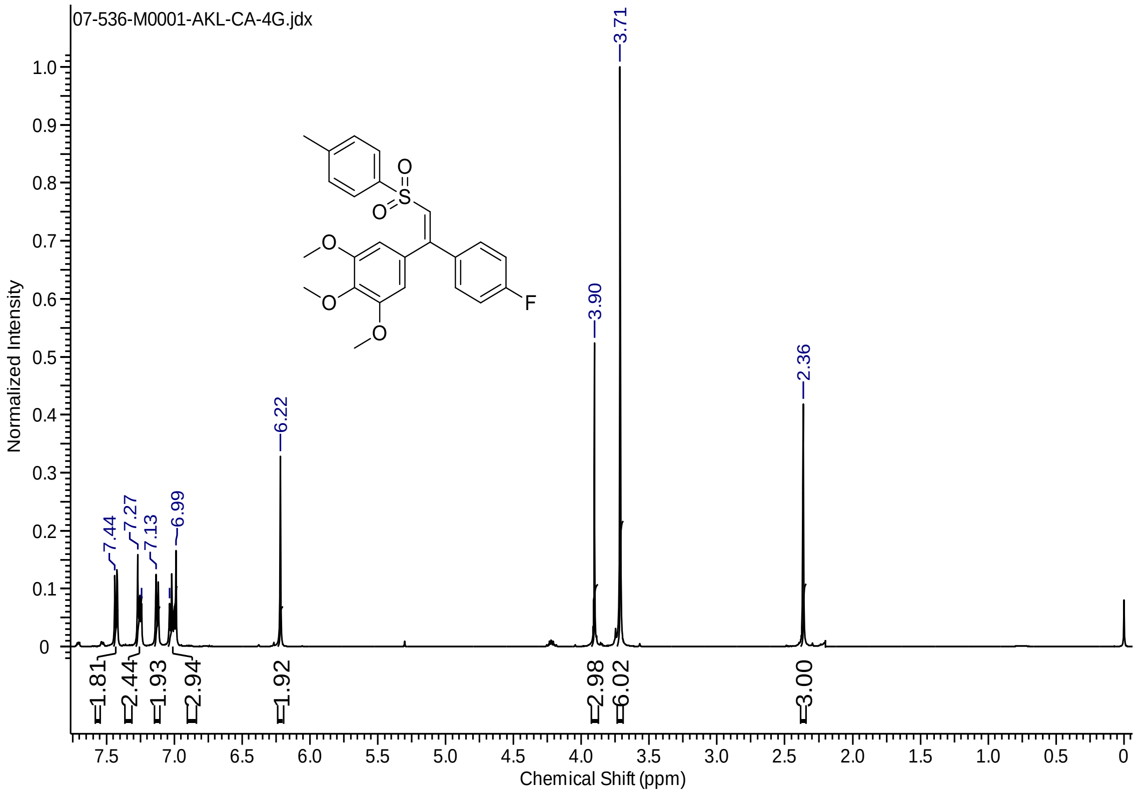

11. 4H, $C_{25}H_{23}F_3O_5S$, (Z)-1,2,3-trimethoxy-5-(2-tosyl-1-(4-(trifluoromethyl)phenyl)vinyl)benzene

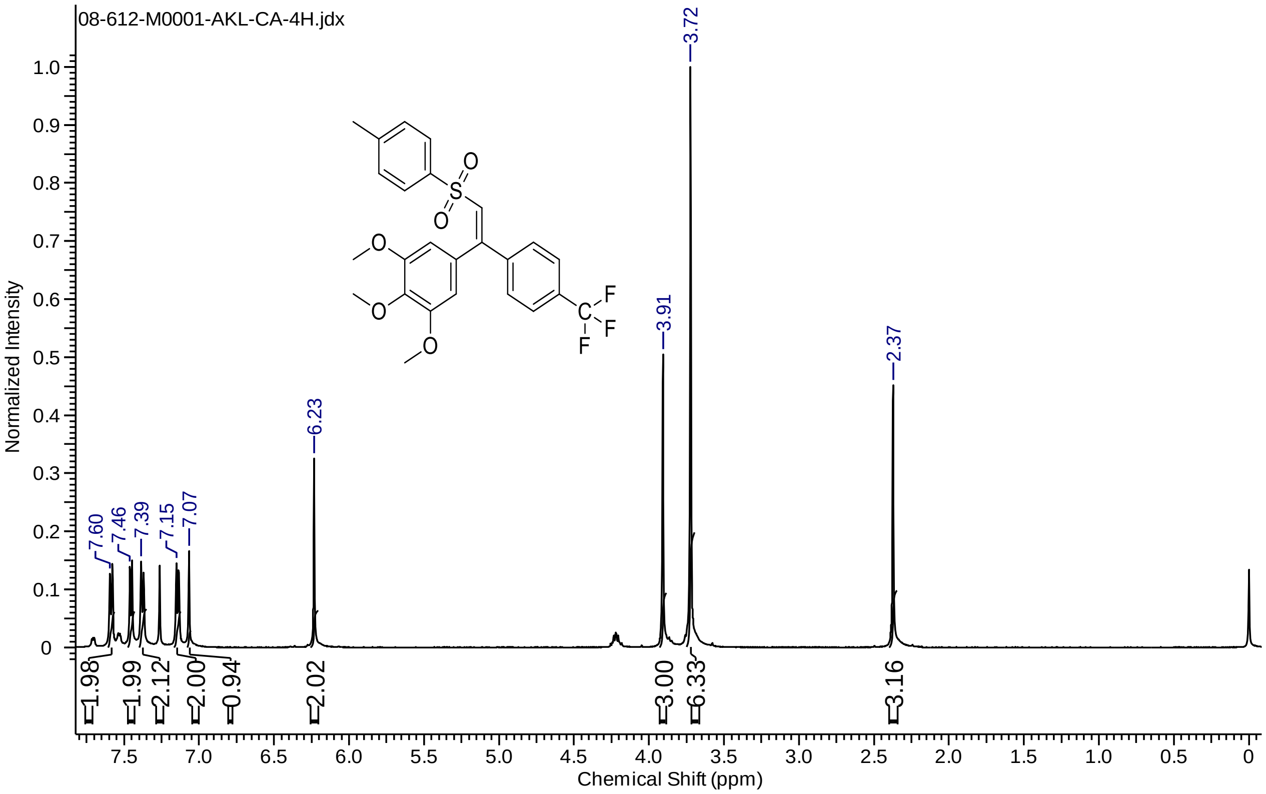

12. 4I, $C_{24}H_{22}F_2O_5S$, (E)-5-(1-(2,4-difluorophenyl)-2-tosylvinyl)-1,2,3-trimethoxybenzene

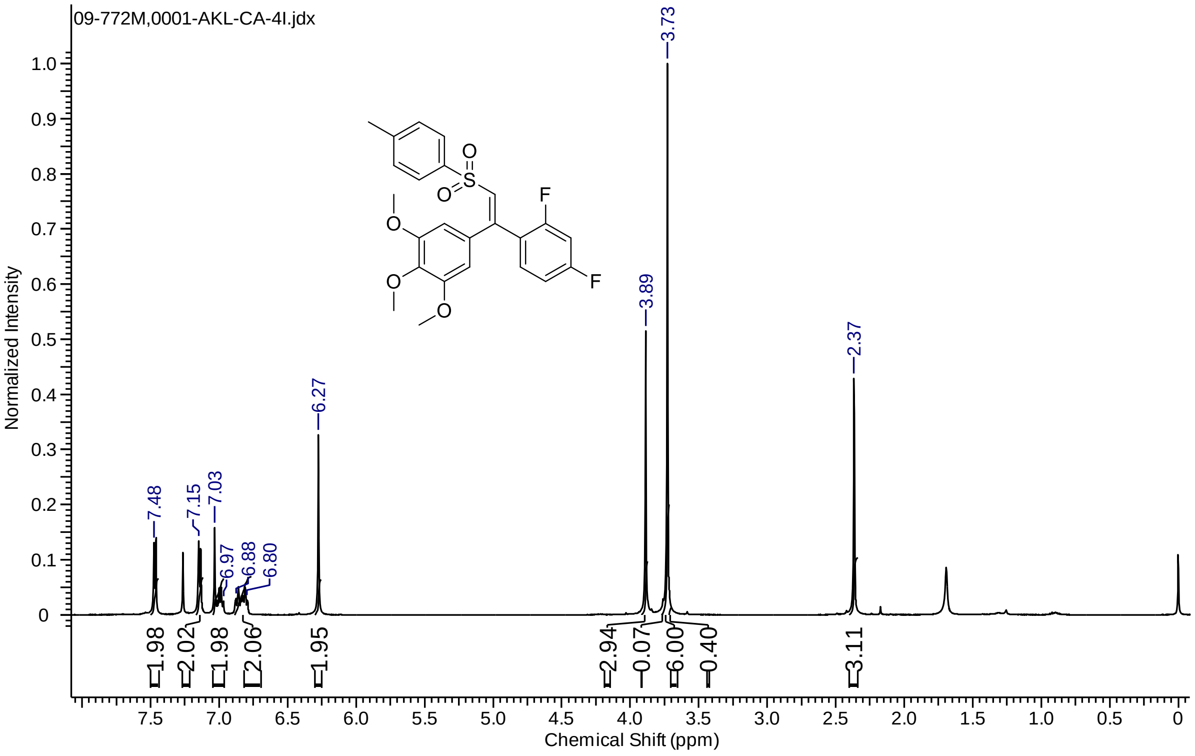

13. 4J,$C_{24}H_{22}F_2O_5S$, (E)-5-(1-(3,4-difluorophenyl)-2-tosylvinyl)-1,2,3-trimethoxybenzene

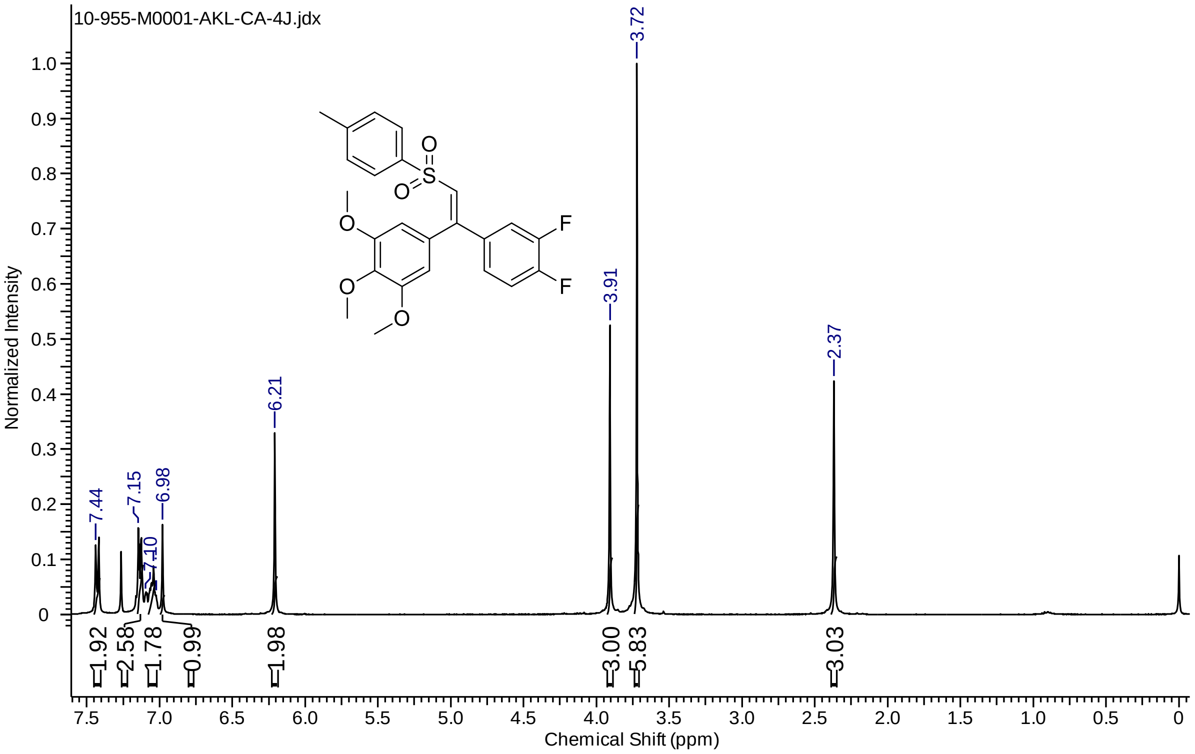

14. 4K, $C_{26}H_{27}FO_6S$, (E)-5-(1-(5-ethoxy-2-fluorophenyl)-2-tosylvinyl)-1,2,3-trimethoxybenzene

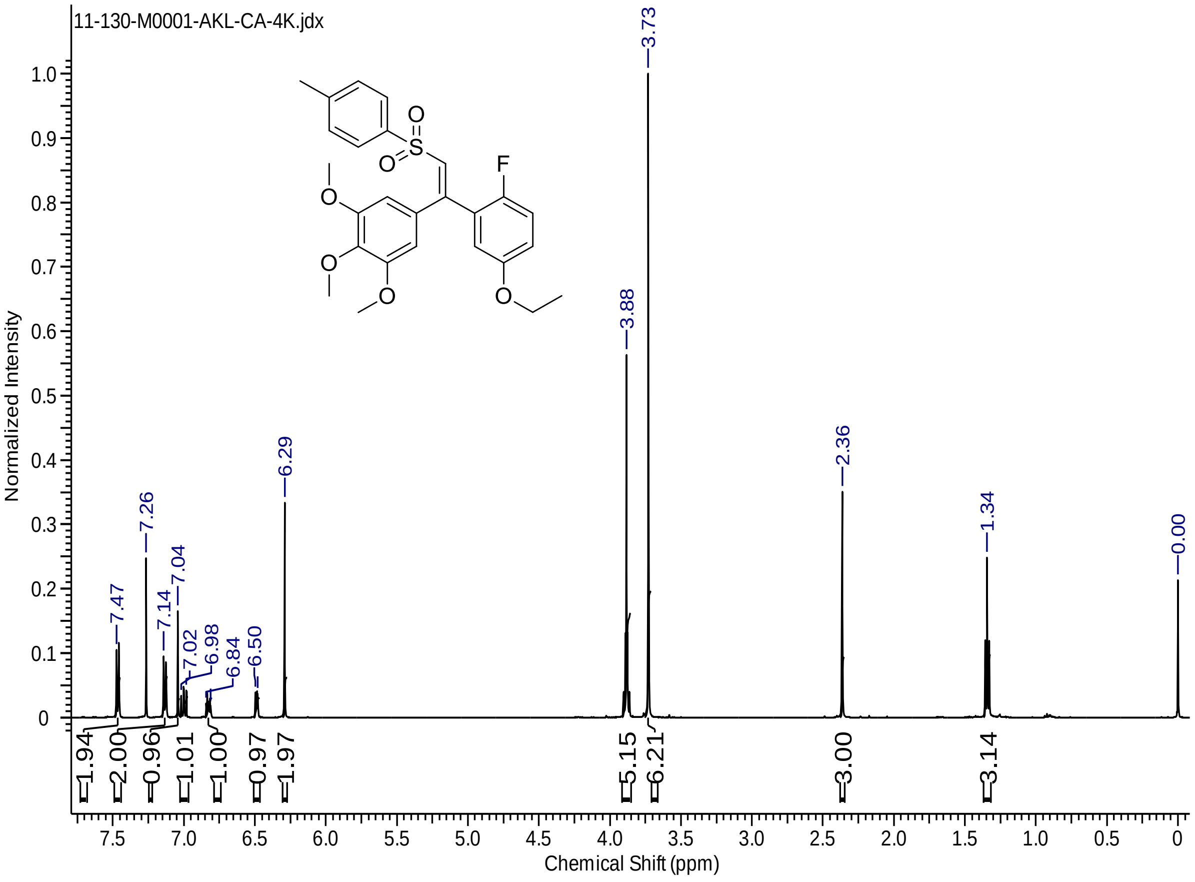

15. 4L, $C_{24}H_{22}ClFO_5S$, (E)-5-(1-(3-chloro-4-fluorophenyl)-2-tosylvinyl)-1,2,3-trimethoxybenzene

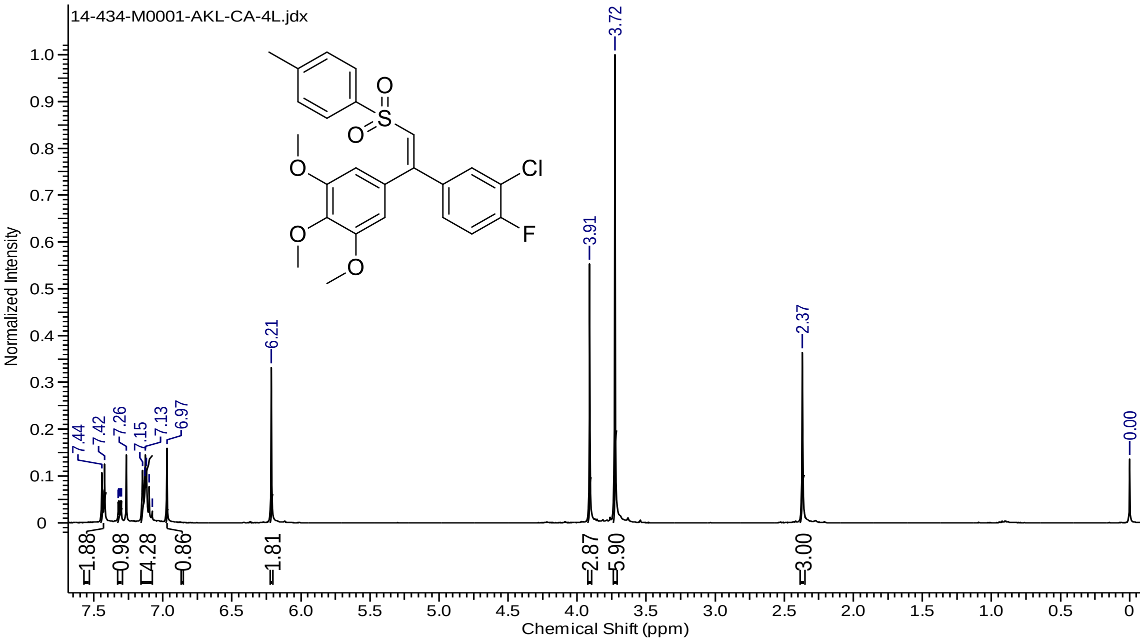

16. 4M, $C_{24}H_{23}ClO_5S$, (Z)-5-(1-(4-chlorophenyl)-2-tosylvinyl)-1,2,3-trimethoxybenzene

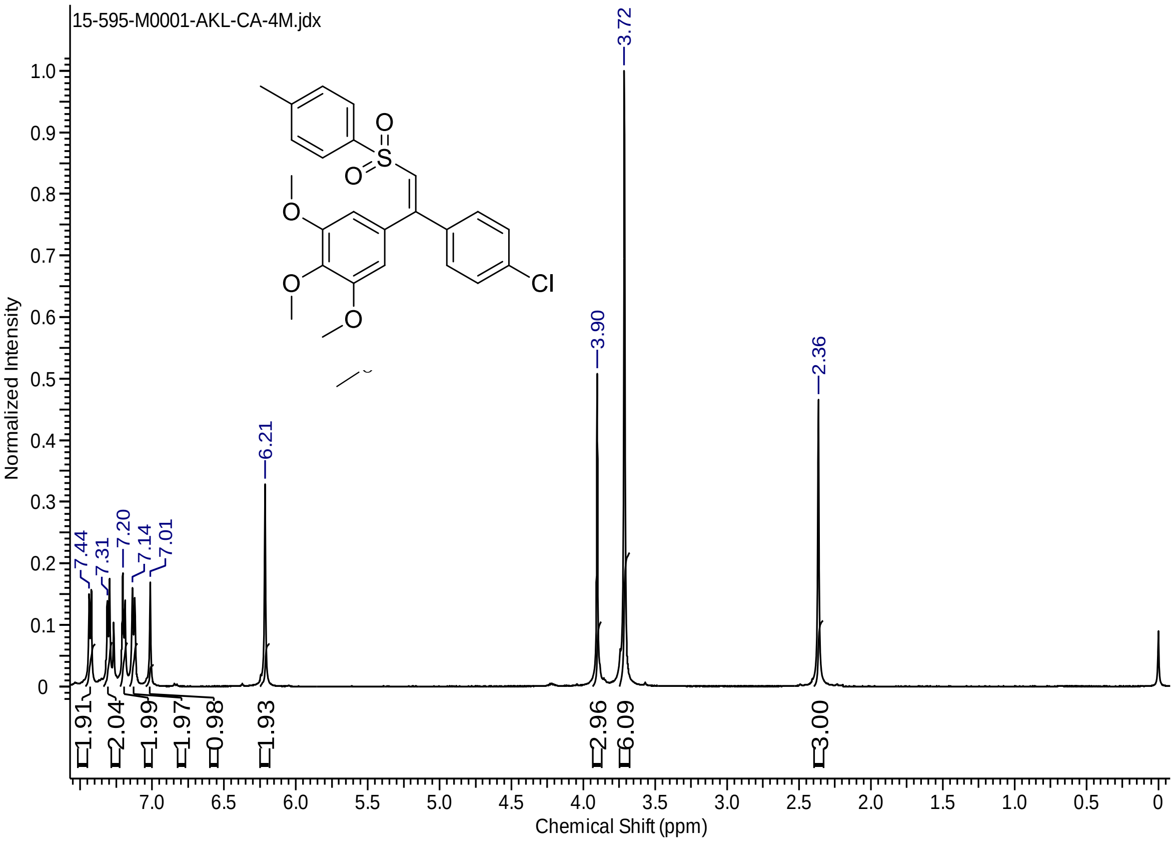

17. 4N, $C_{24}H_{22}Cl_2O_5S$, (E)-5-(1-(3,4-dichlorophenyl)-2-tosylvinyl)-1,2,3-trimethoxybenzene

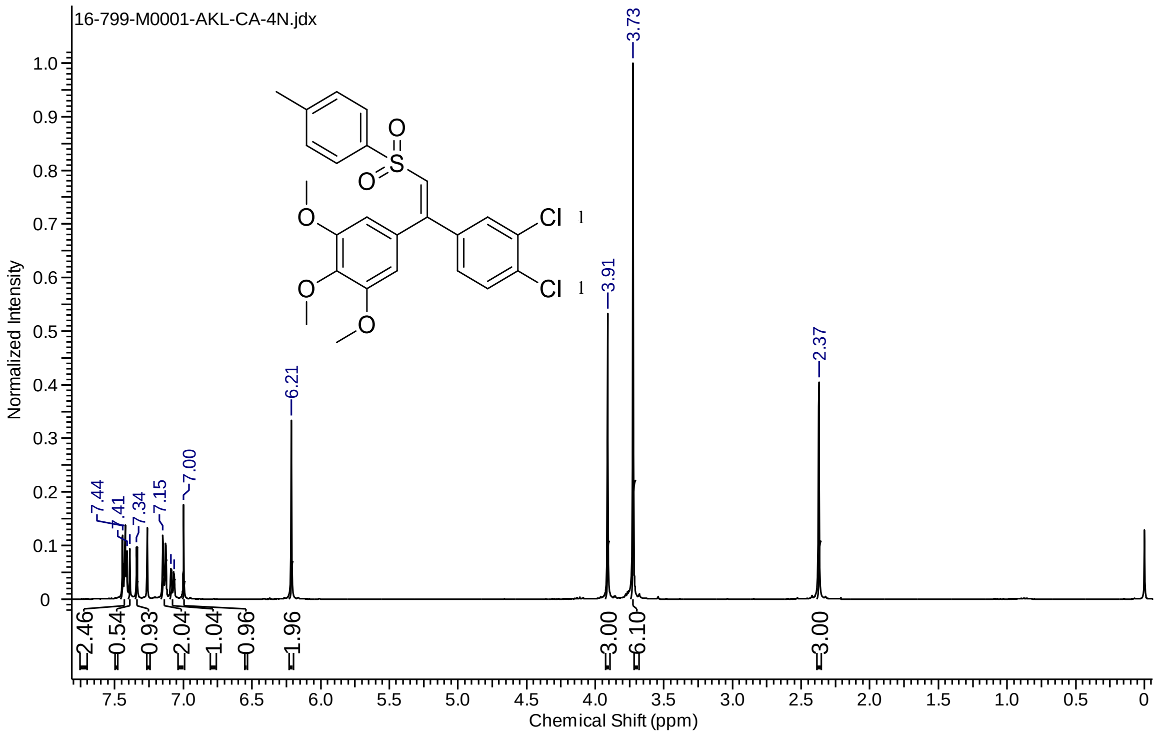

18. 4O, $C_{25}H_{23}NO_5S$, (Z)-4-(2-tosyl-1-(3,4,5-trimethoxyphenyl)vinyl)benzonitrile

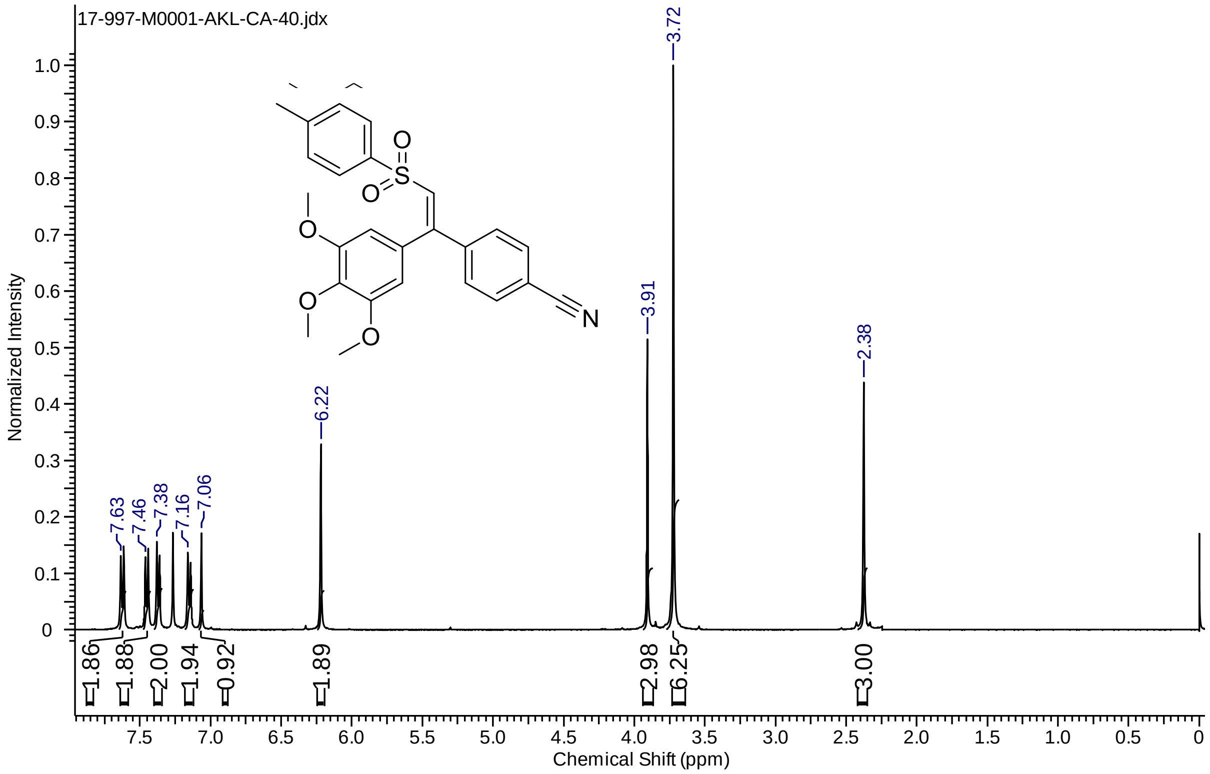

19. 4Q, $C_{25}H_{22}F_4O_5S$, (E)-5-(1-(2-fluoro-3-(trifluoromethyl)phenyl)-2-tosylvinyl)-1,2,3-trimethoxybenzene

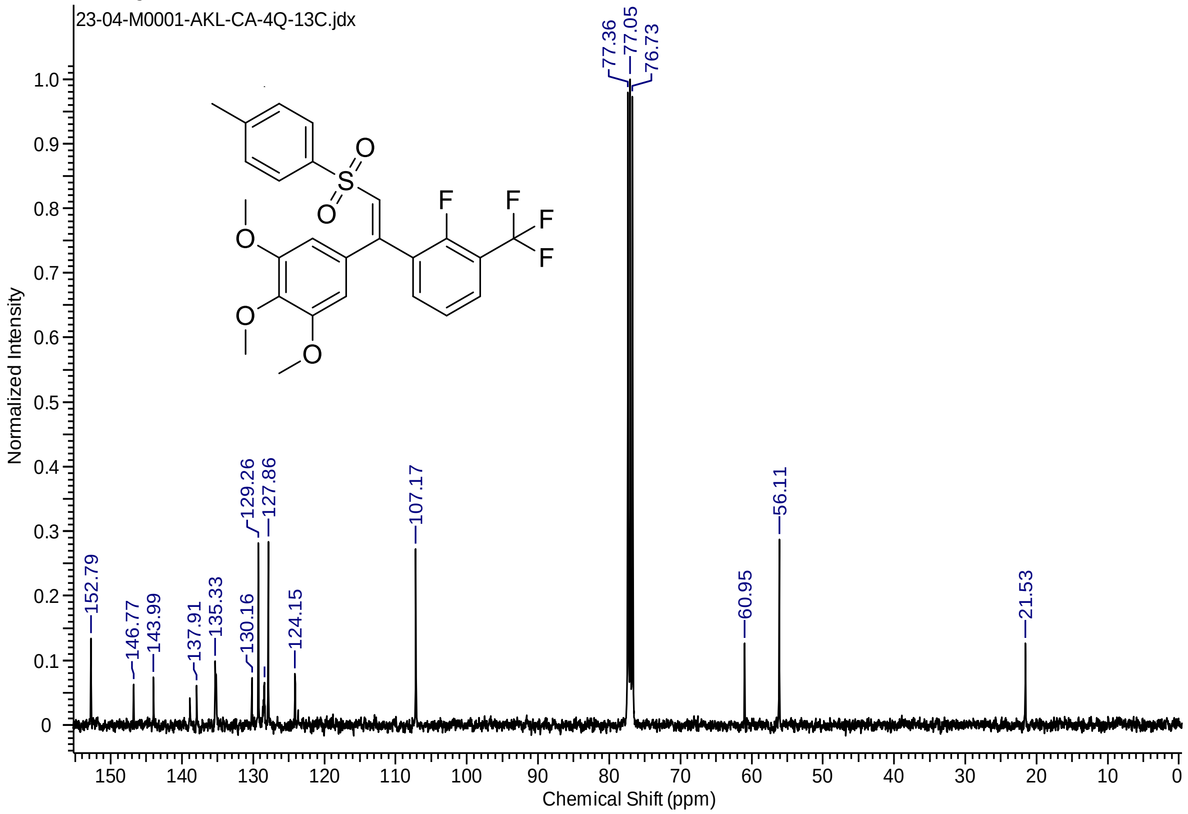

20. 4R, $C_{25}H_{25}FO_5S$, (E)-5-(1-(2-fluoro-5-methylphenyl)-2-tosylvinyl)-1,2,3-trimethoxybenzene

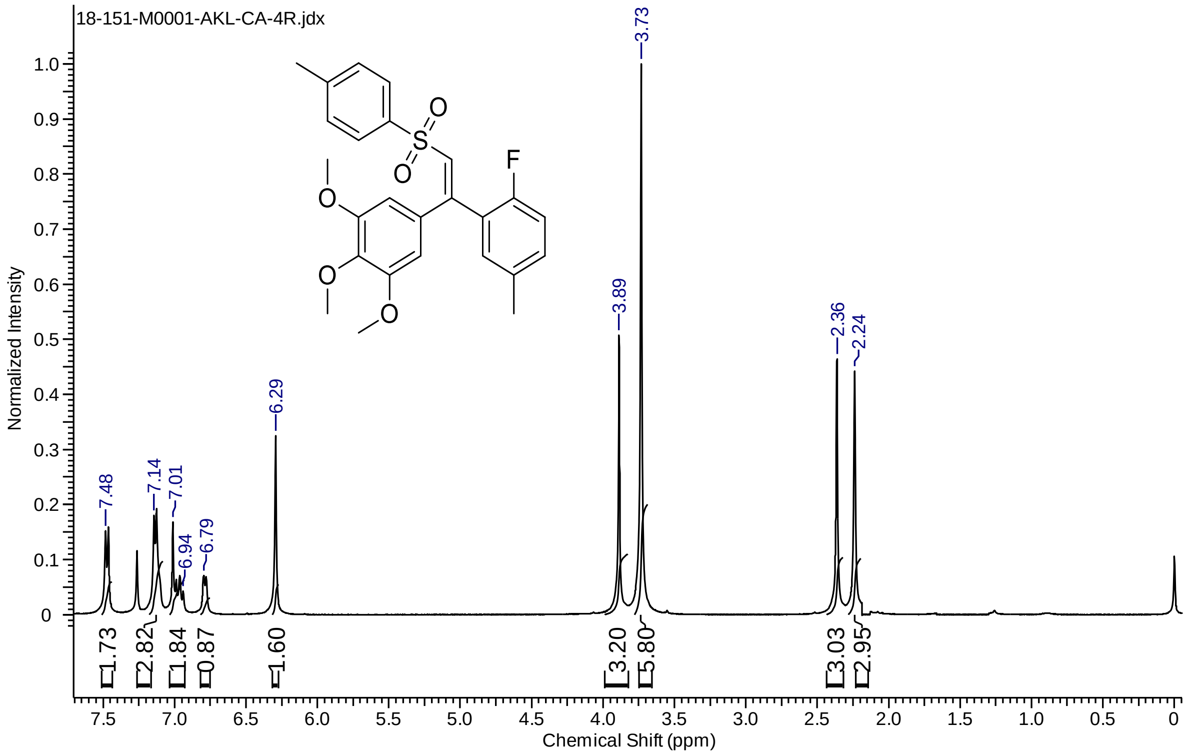

21. 4T, $C_{25}H_{25}FO_5S$, (E)-5-(1-(3-fluoro-4-methylphenyl)-2-tosylvinyl)-1,2,3-trimethoxybenzene

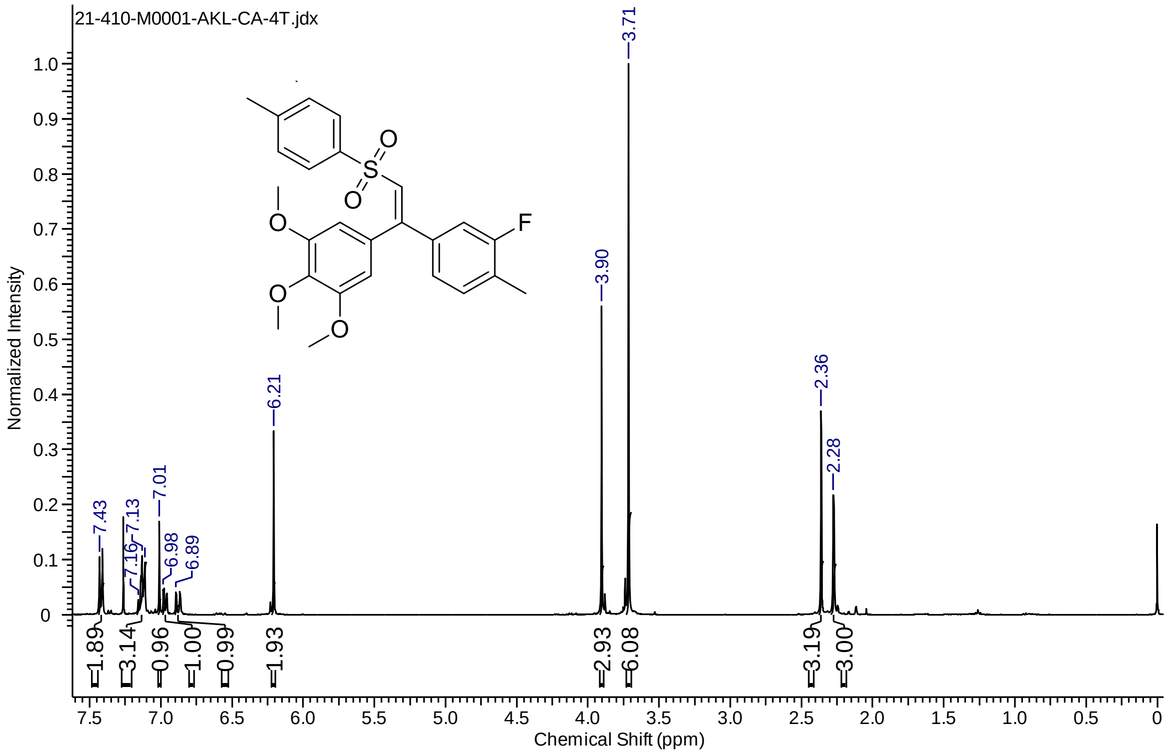

22. 4U, $C_{30}H_{28}O_5S$, (Z)-4-(2-tosyl-1-(3,4,5-trimethoxyphenyl)vinyl)-1,1'-biphenyl

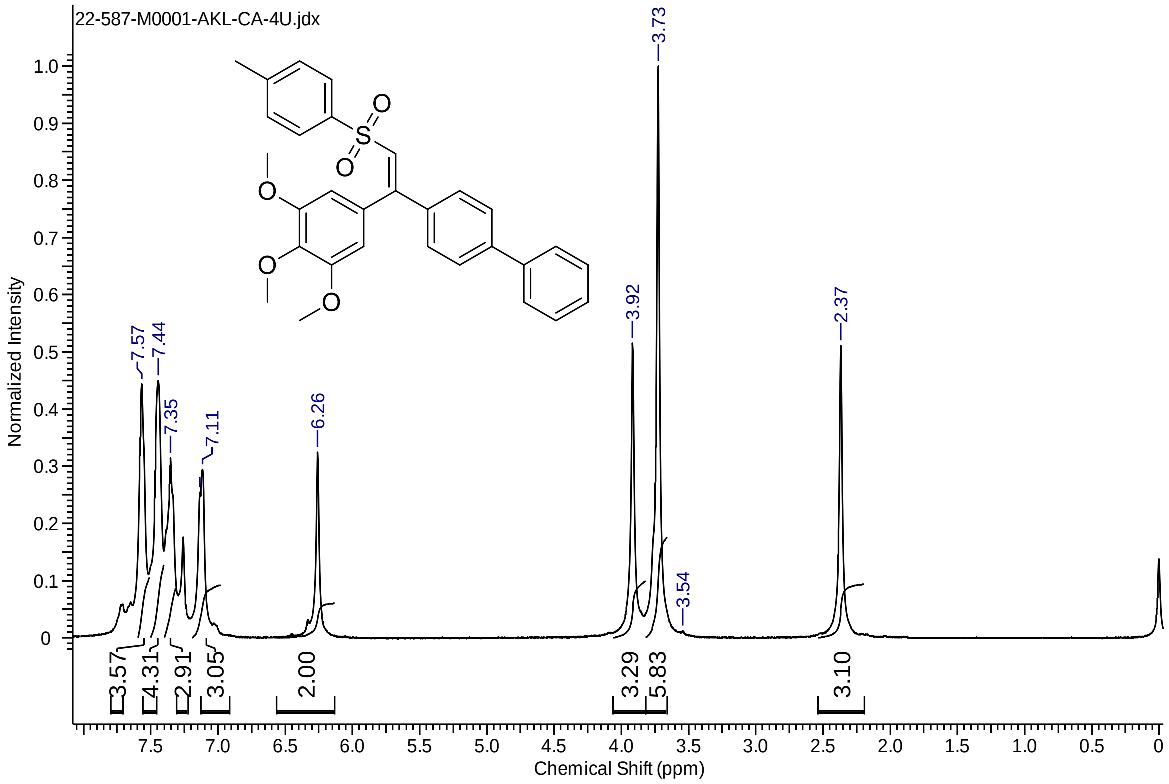

23. 4V, $C_{18}H_{18}O_5S$, 4-methylphenyl (3,4,5-trimethoxyphenyl)ethynyl sulfone

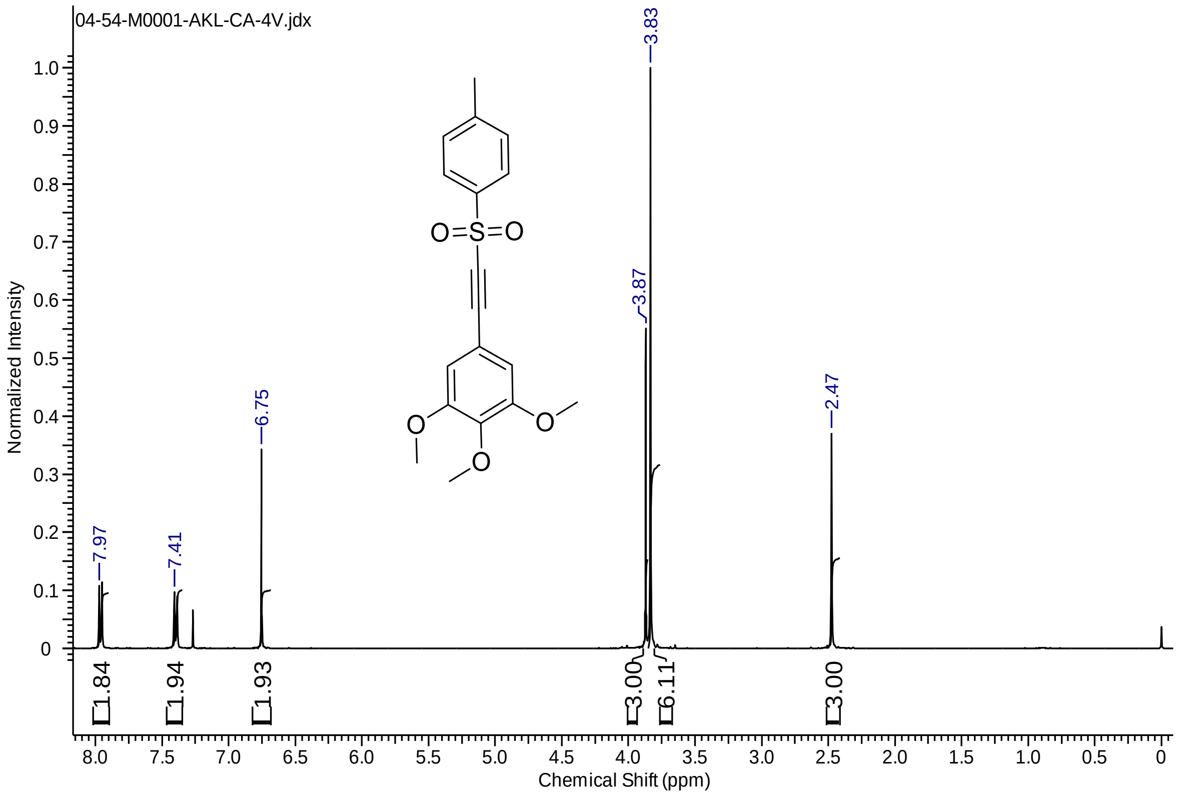

13C NMR SPECTRA OF SYNTHESIZED COMPOUNDS

1. $C_{11}H_{12}Br_2O_3$; 5-(2,2-dibromovinyl)-1,2,3-trimethoxybenzene

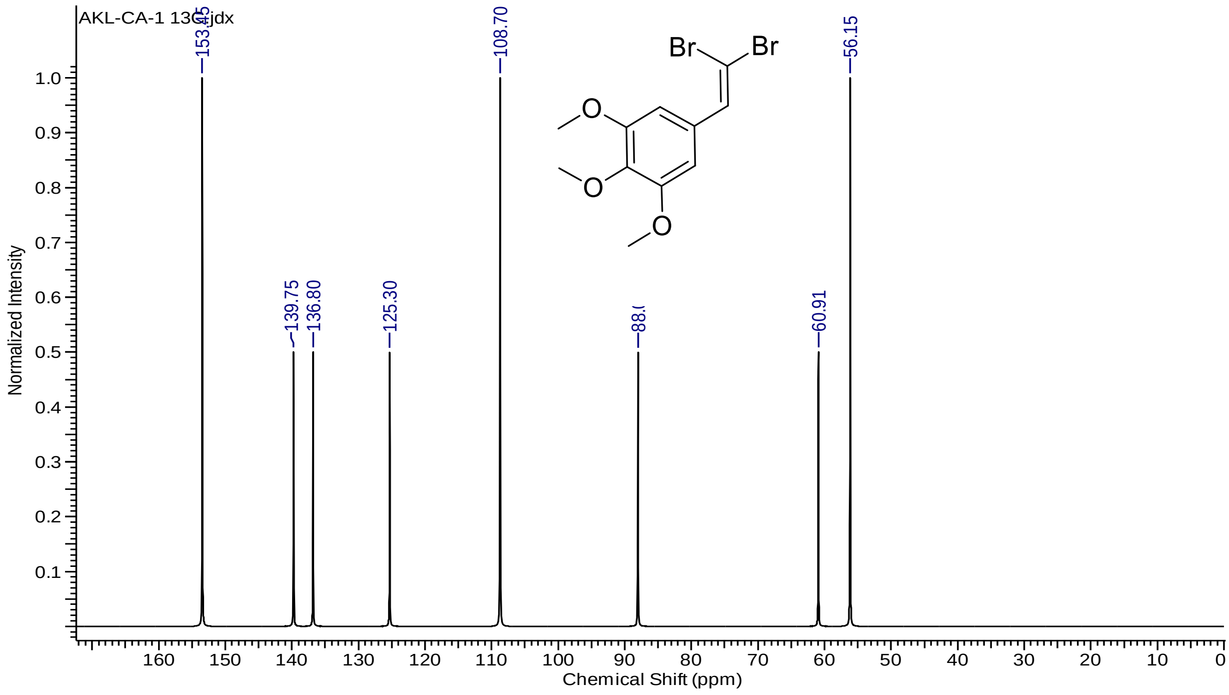

2. $C_{11}H_{12}O_3$, 5-ethynyl-1,2,3-trimethoxybenzene

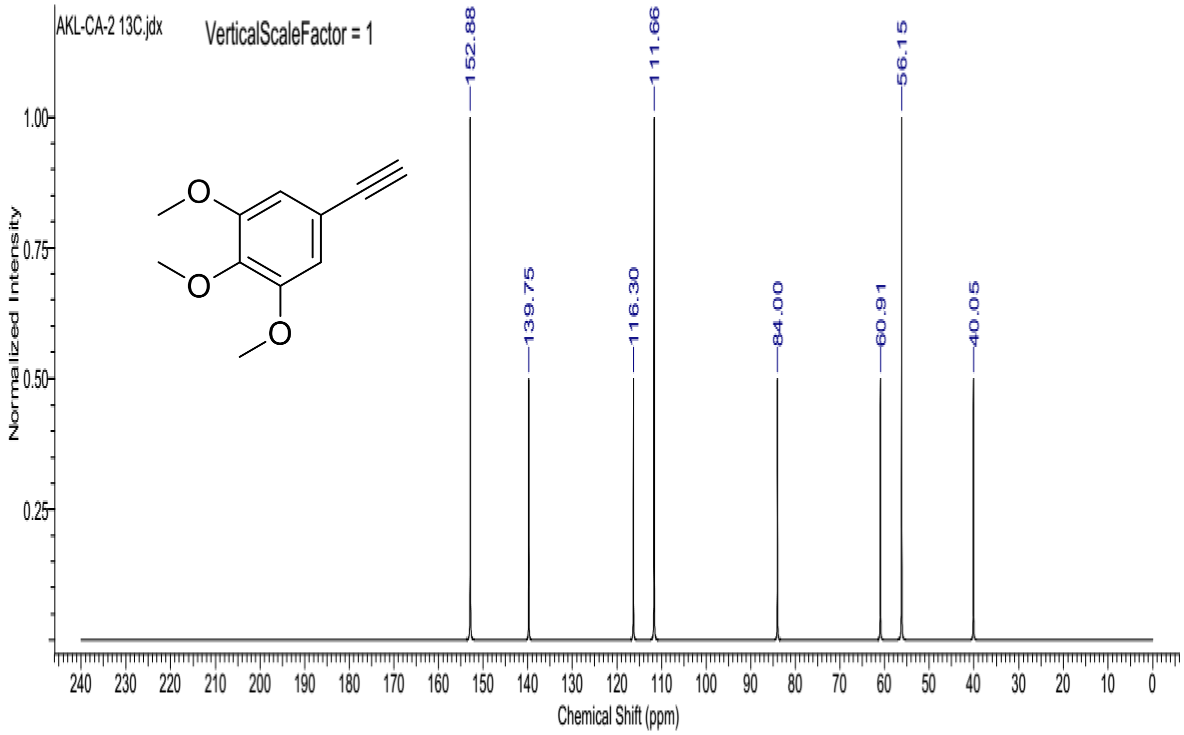

3. $C_{18}H_{19}IO_5S$, (E)-5-(1-iodo-2-tosylvinyl)-1,2,3-trimethoxybenzene

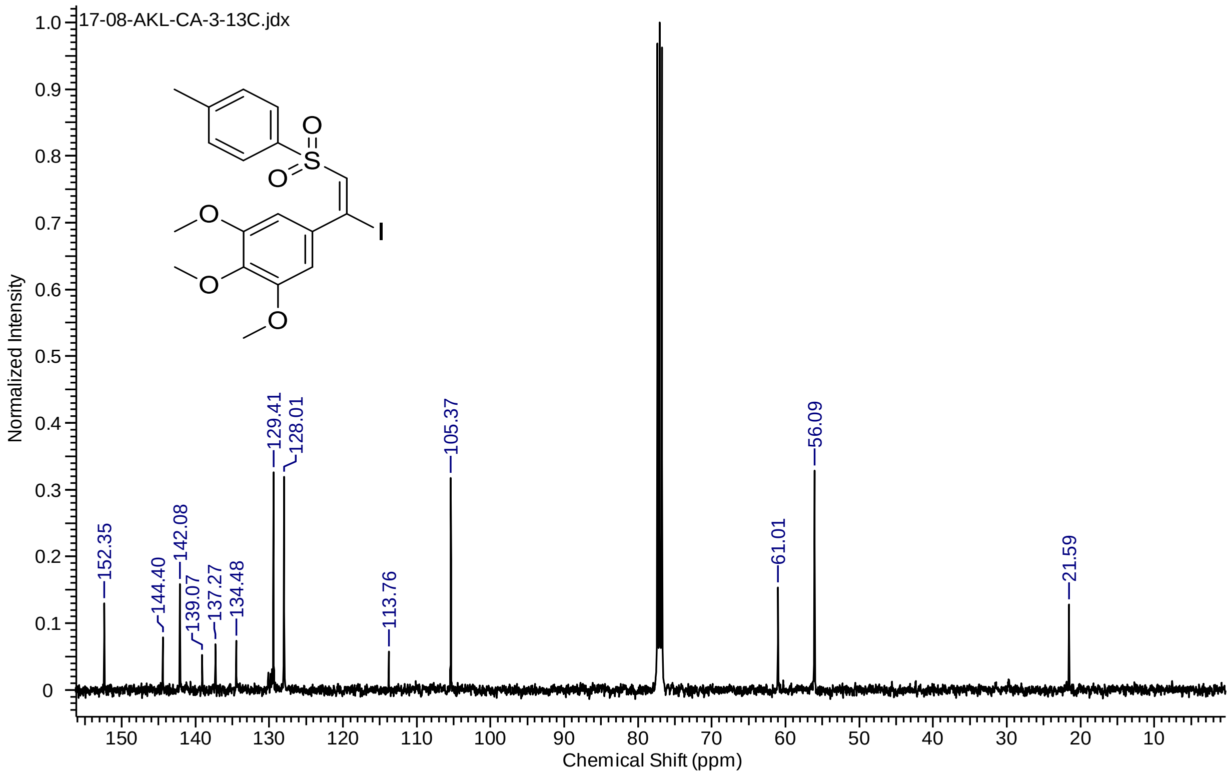

4. 4A, $C_{24}H_{24}O_5S$, (Z)-1,2,3-trimethoxy-5-(1-phenyl-2-tosylvinyl)benzene

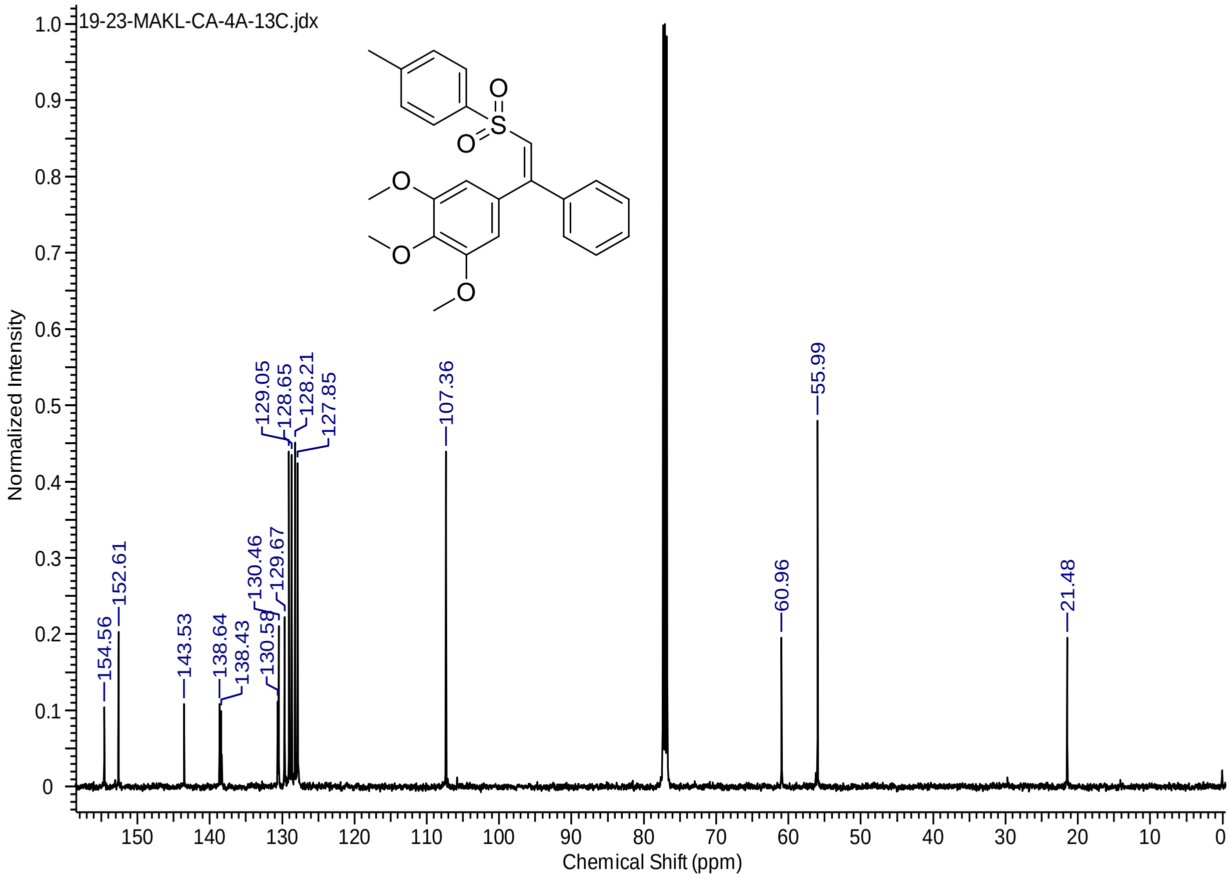

5. 4B, (Z)-1,2,3-trimethoxy-5-(1-(4-methoxyphenyl)-2-tosylvinyl)benzene

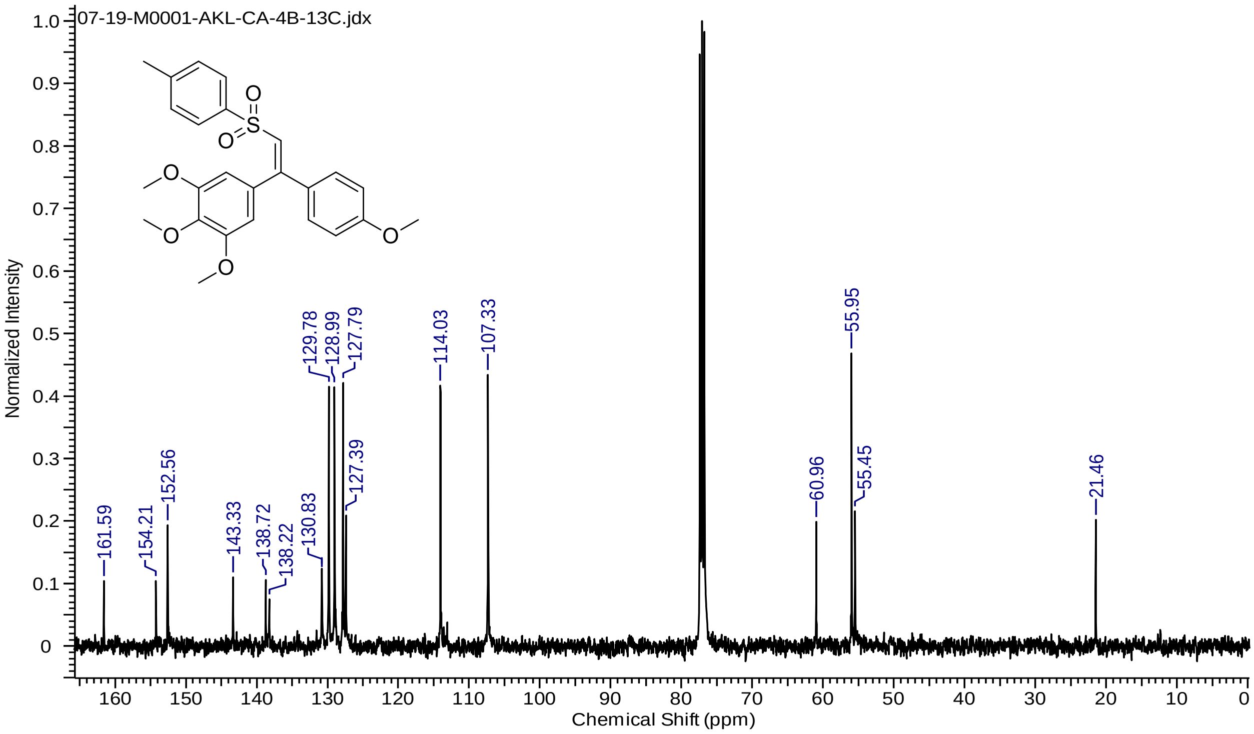

6. 4C, $C_{26}H_{28}O_7S$, (E)-5-(1-(2,4-dimethoxyphenyl)-2-tosylvinyl)-1,2,3-trimethoxybenzene

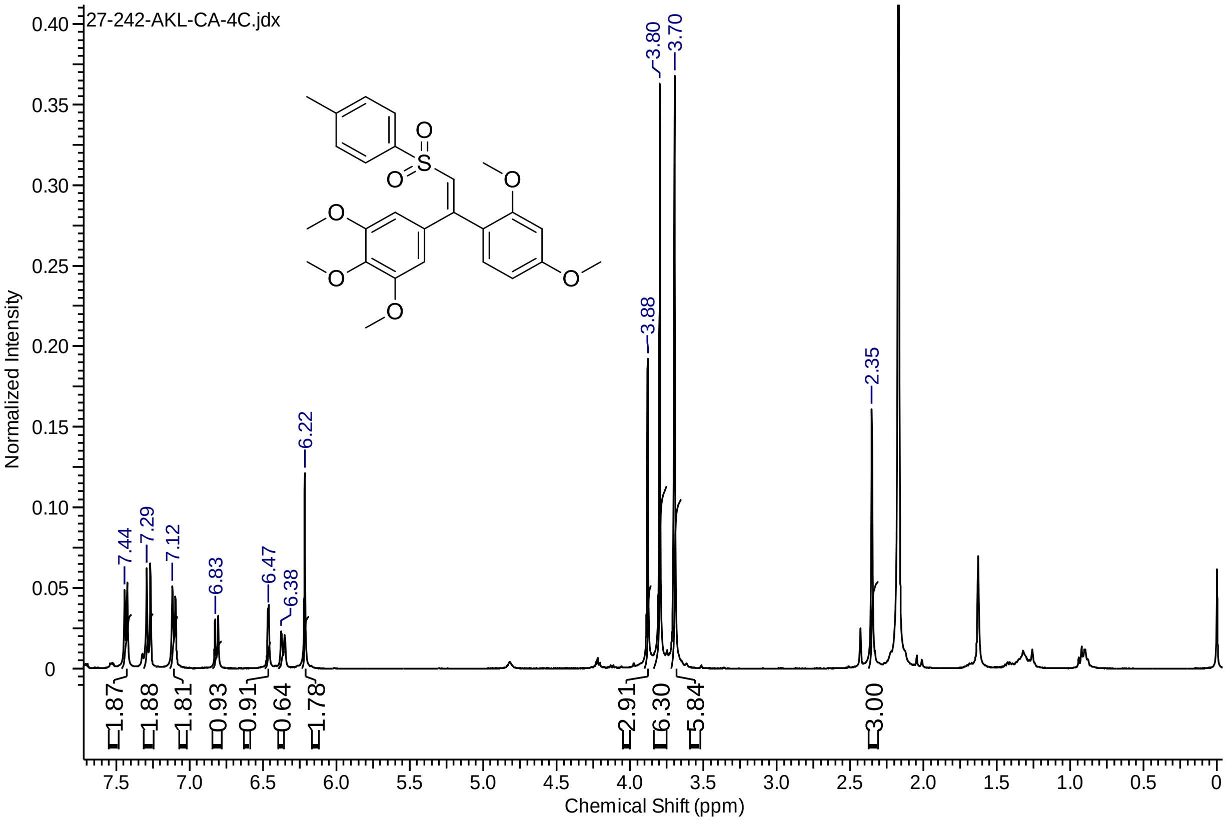

7. 4D, $C_{26}H_{28}O_7S$, (Z)-5-(1-(3,4-dimethoxyphenyl)-2-tosylvinyl)-1,2,3-trimethoxybenzene

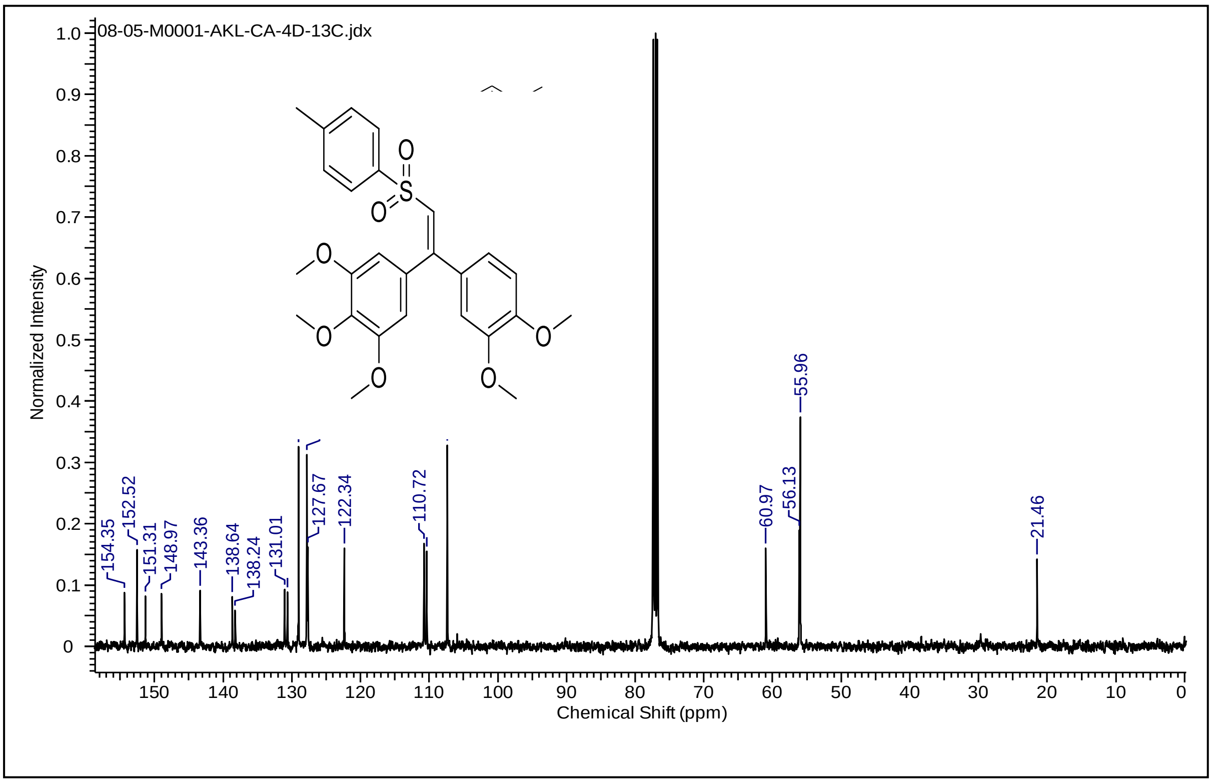

8. 4E, $C_{26}H_{28}O_7S$, (Z)-5-(1-(3,5-dimethoxyphenyl)-2-tosylvinyl)-1,2,3-trimethoxybenzene

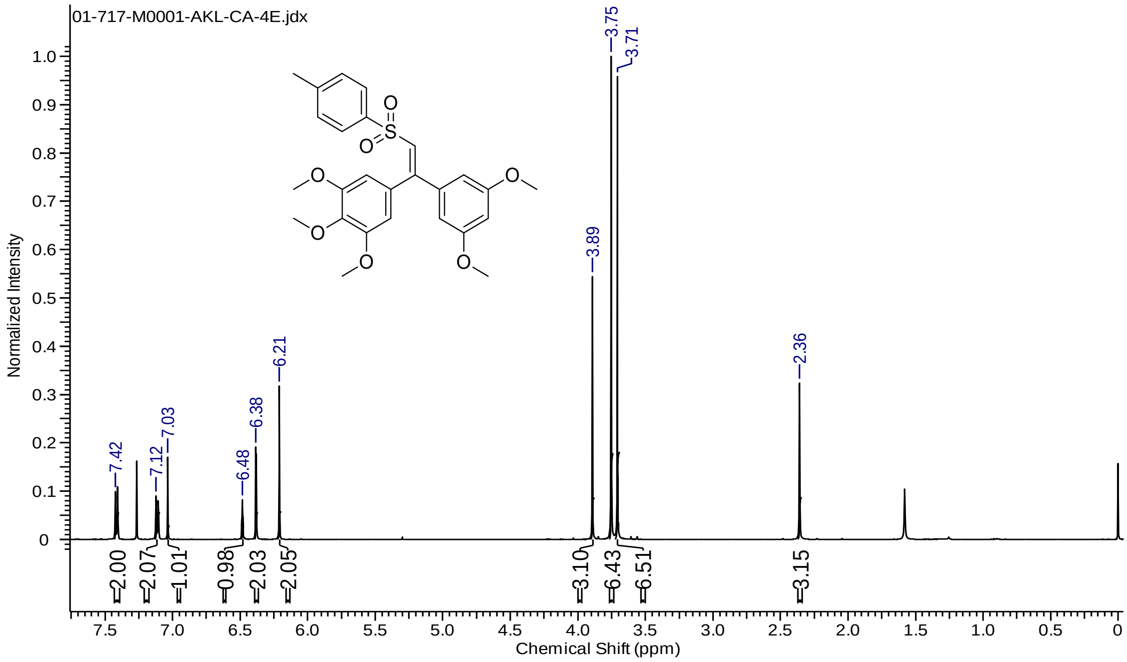

9. 4F, $C_{18}H_{18}O_5S$, 4-methylphenyl (3,4,5-trimethoxyphenyl)ethynyl sulfone

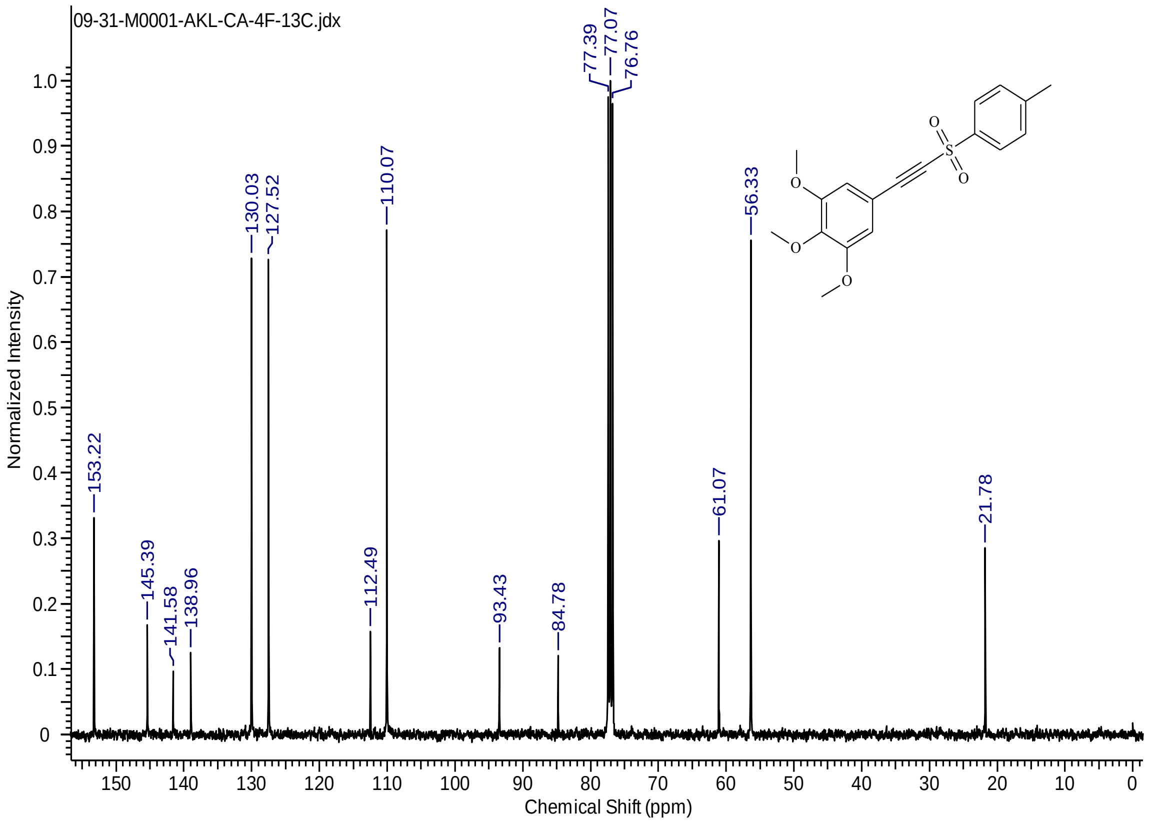

10. 4G, $C_{24}H_{23}FO_5S$, (Z)-5-(1-(4-fluorophenyl)-2-tosylvinyl)-1,2,3-trimethoxybenzene

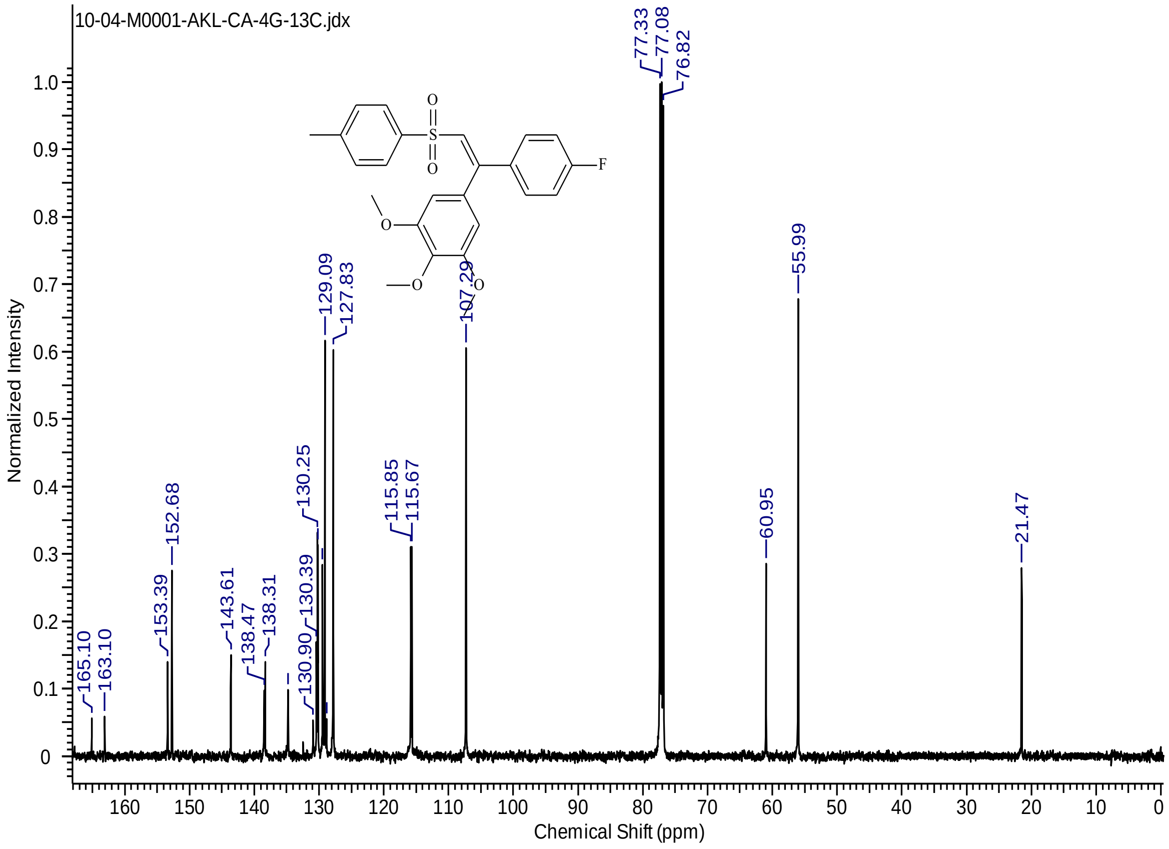

11. 4H, $C_{25}H_{23}F_3O_5S$, (Z)-1,2,3-trimethoxy-5-(2-tosyl-1-(4-(trifluoromethyl)phenyl)vinyl)benzene

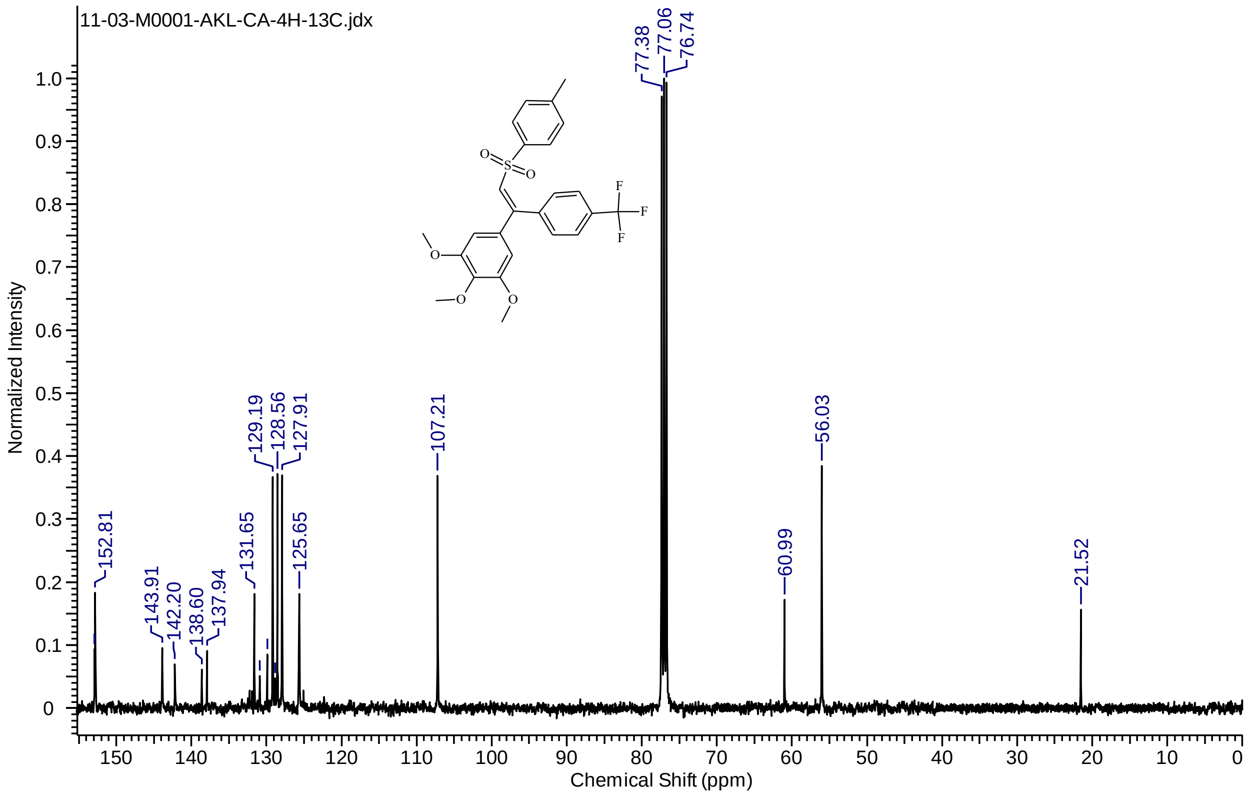

12. 4I, $C_{24}H_{22}F_2O_5S$, (E)-5-(1-(2,4-difluorophenyl)-2-tosylvinyl)-1,2,3-trimethoxybenzene

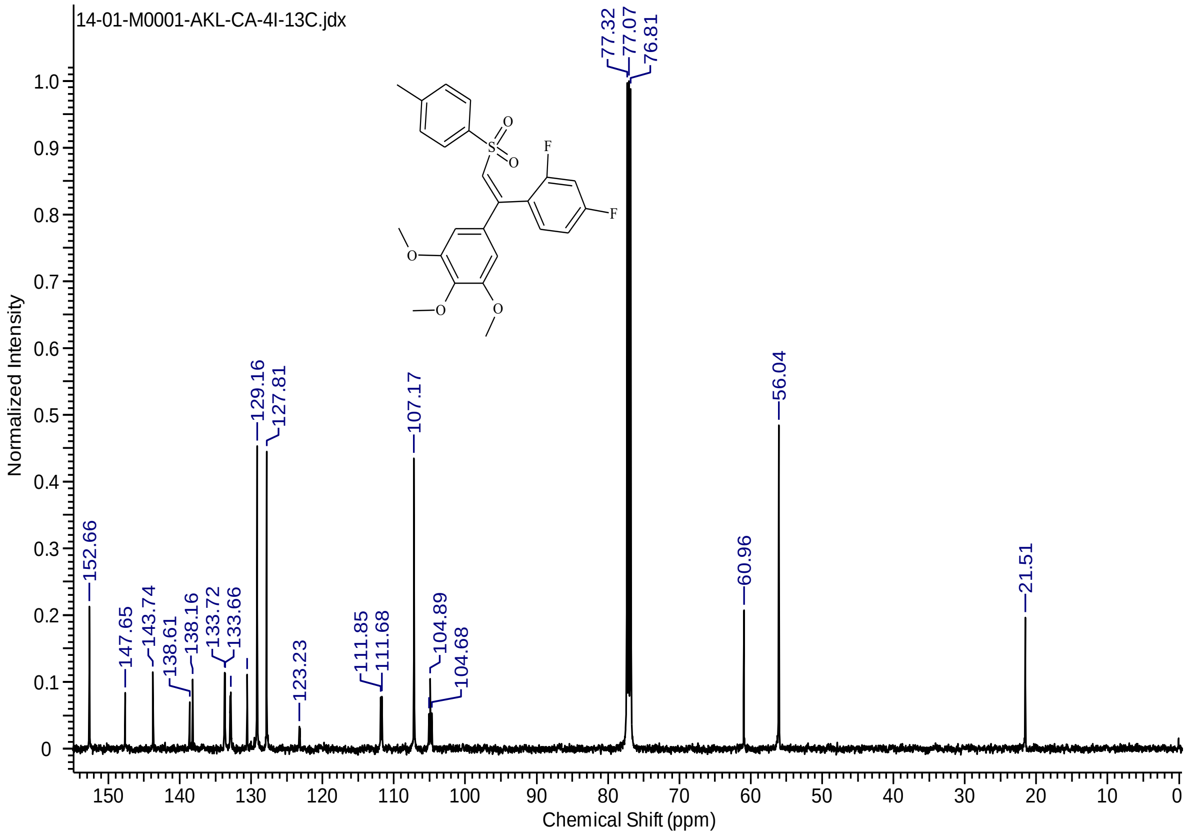

13. 4J, $C_{24}H_{22}F_2O_5S$, (E)-5-(1-(3,4-difluorophenyl)-2-tosylvinyl)-1,2,3-trimethoxybenzene

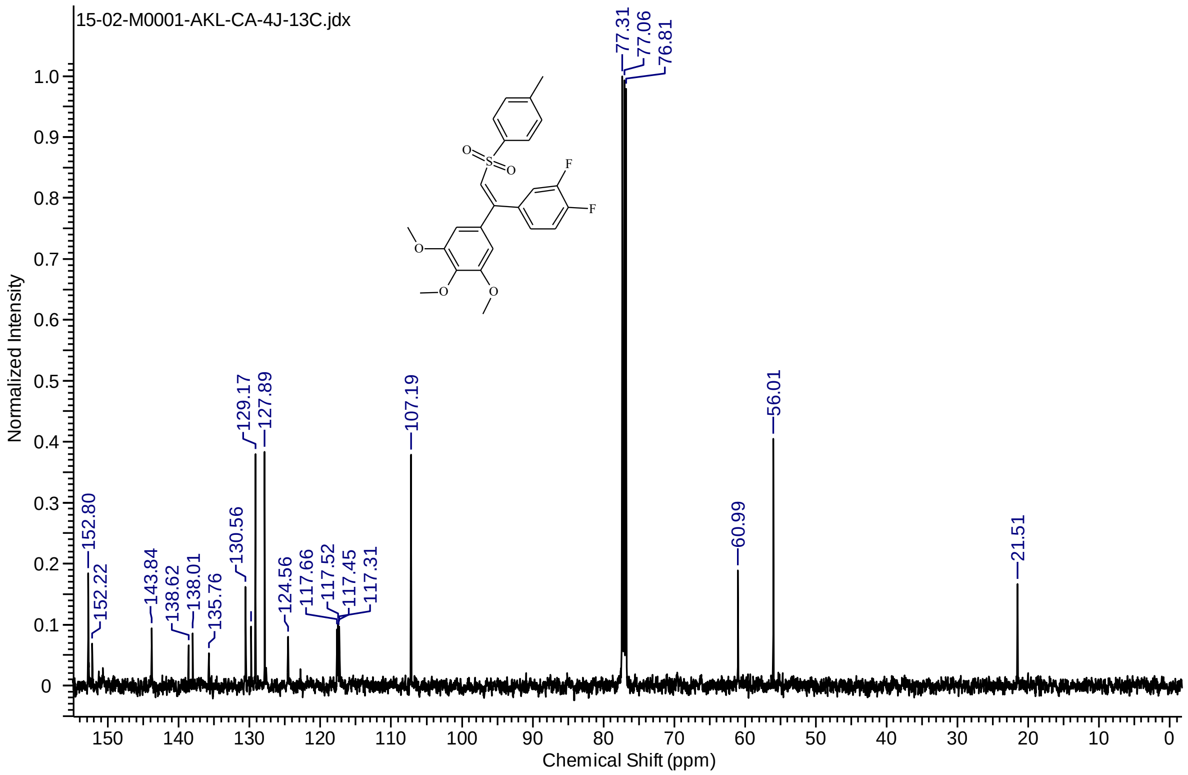

14. 4K, $C_{26}H_{27}FO_6S$, (E)-5-(1-(5-ethoxy-2-fluorophenyl)-2-tosylvinyl)-1,2,3-trimethoxybenzene

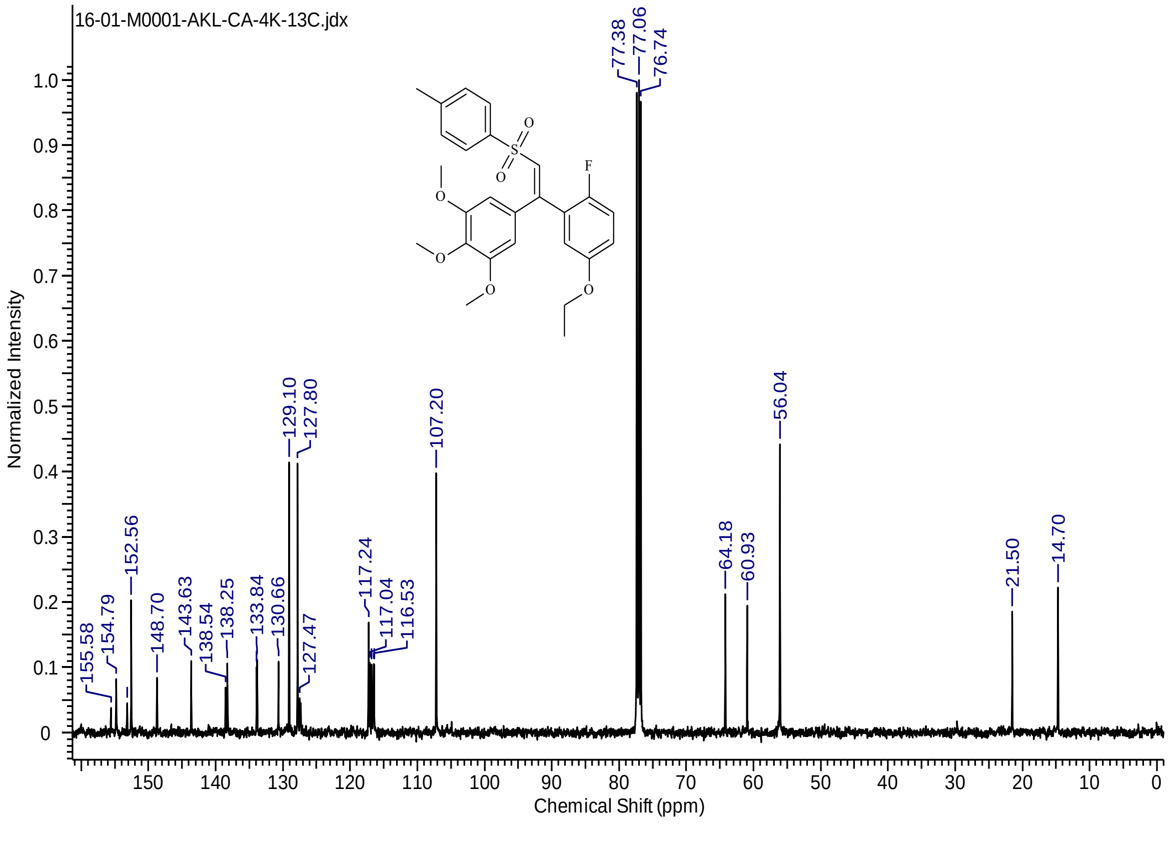

15. 4L, $C_{24}H_{22}ClFO_5S$, (E)-5-(1-(3-chloro-4-fluorophenyl)-2-tosylvinyl)-1,2,3-trimethoxybenzene

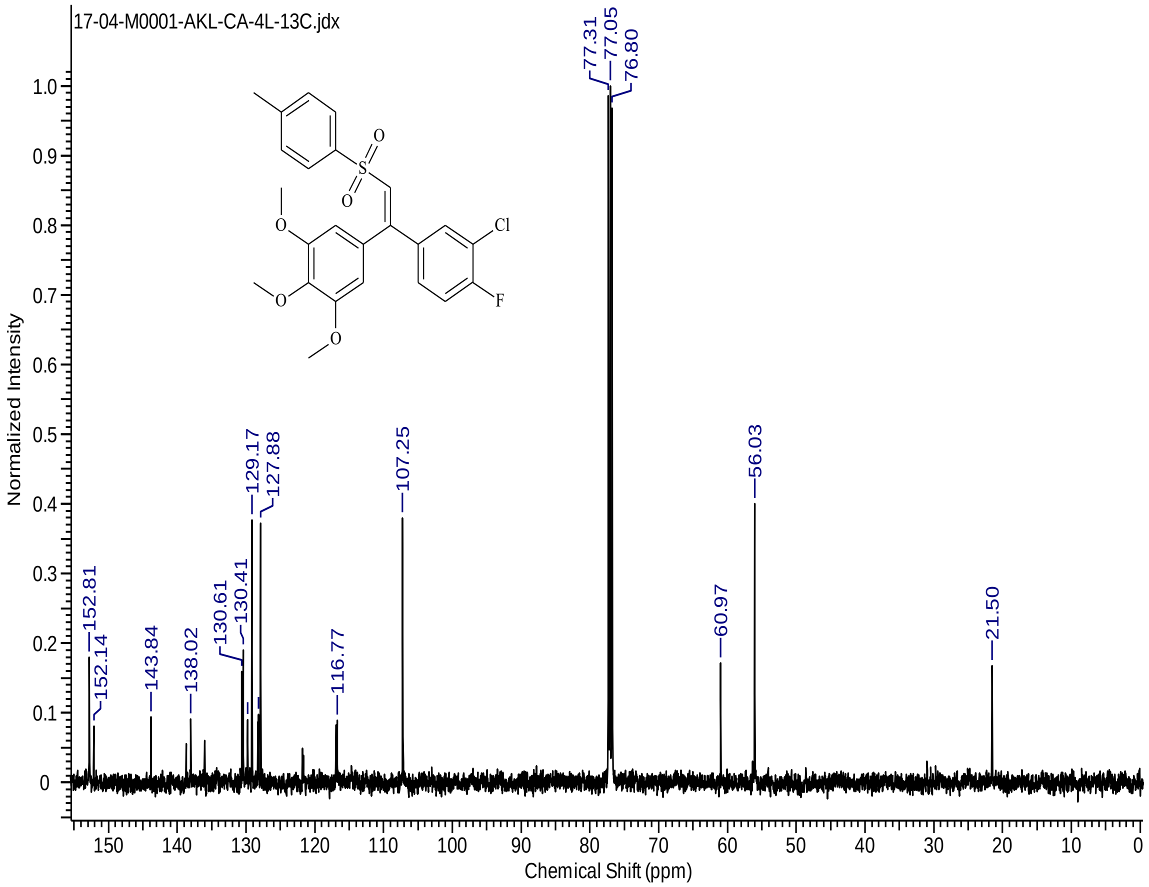

16. 4M, $C_{24}H_{23}ClO_5S$, (Z)-5-(1-(4-chlorophenyl)-2-tosylvinyl)-1,2,3-trimethoxybenzene

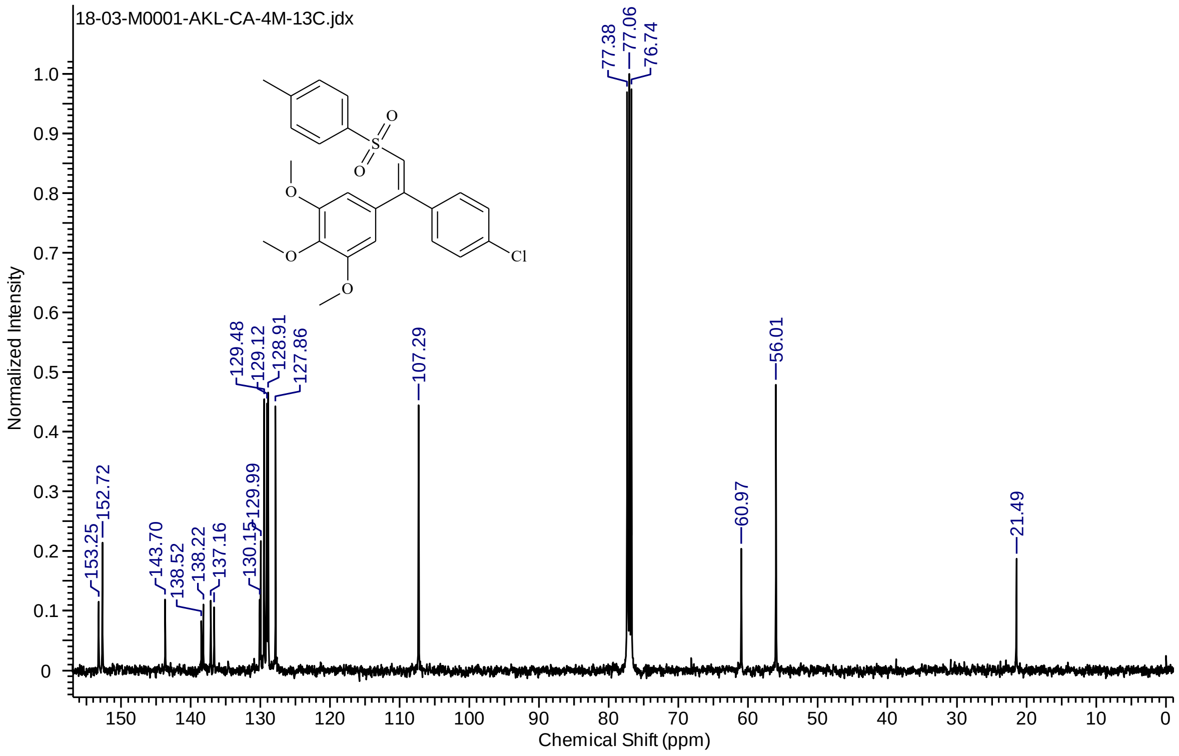

17. 4N, $C_{24}H_{22}Cl_2O_5S$, (E)-5-(1-(3,4-dichlorophenyl)-2-tosylvinyl)-1,2,3-trimethoxybenzene

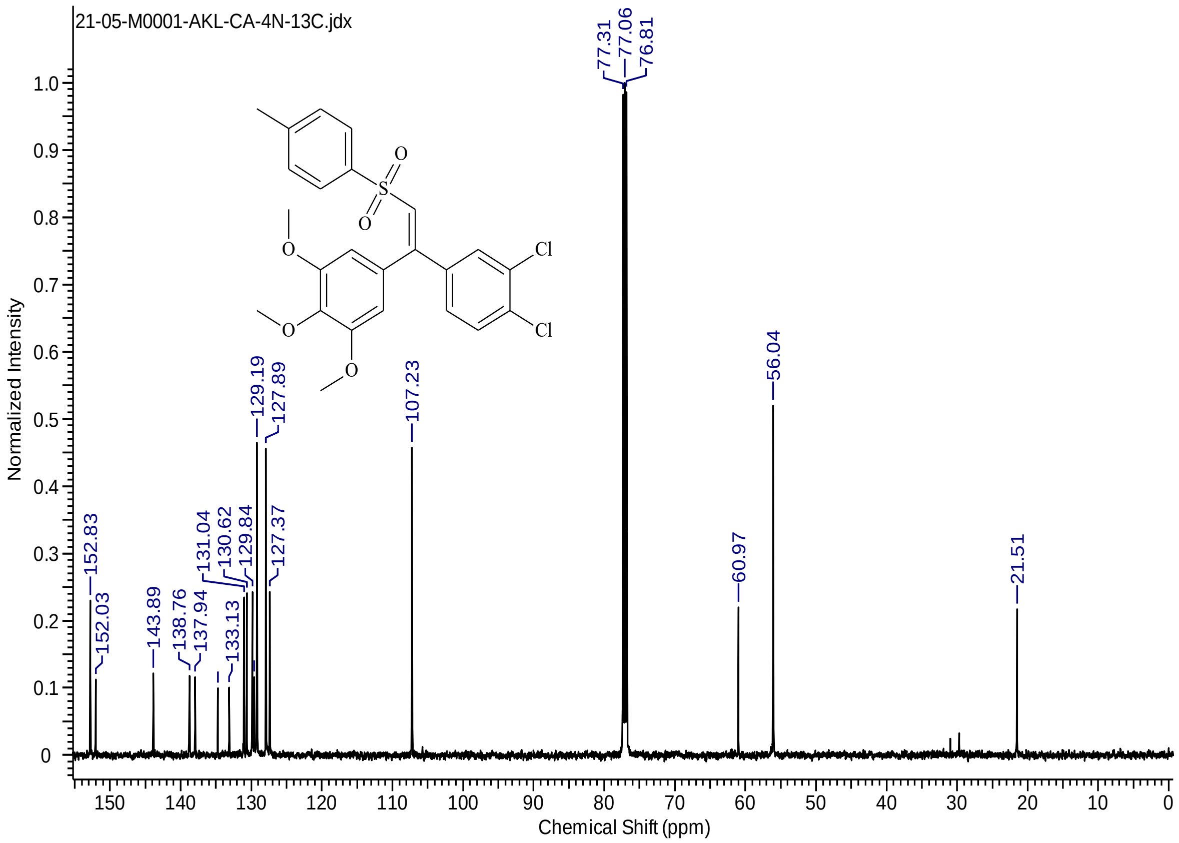

18.4O,$C_{25}H_{23}NO_5S$,(Z)-4-(2-tosyl-1-(3,4,5-trimethoxyphenyl)vinyl)benzonitrile

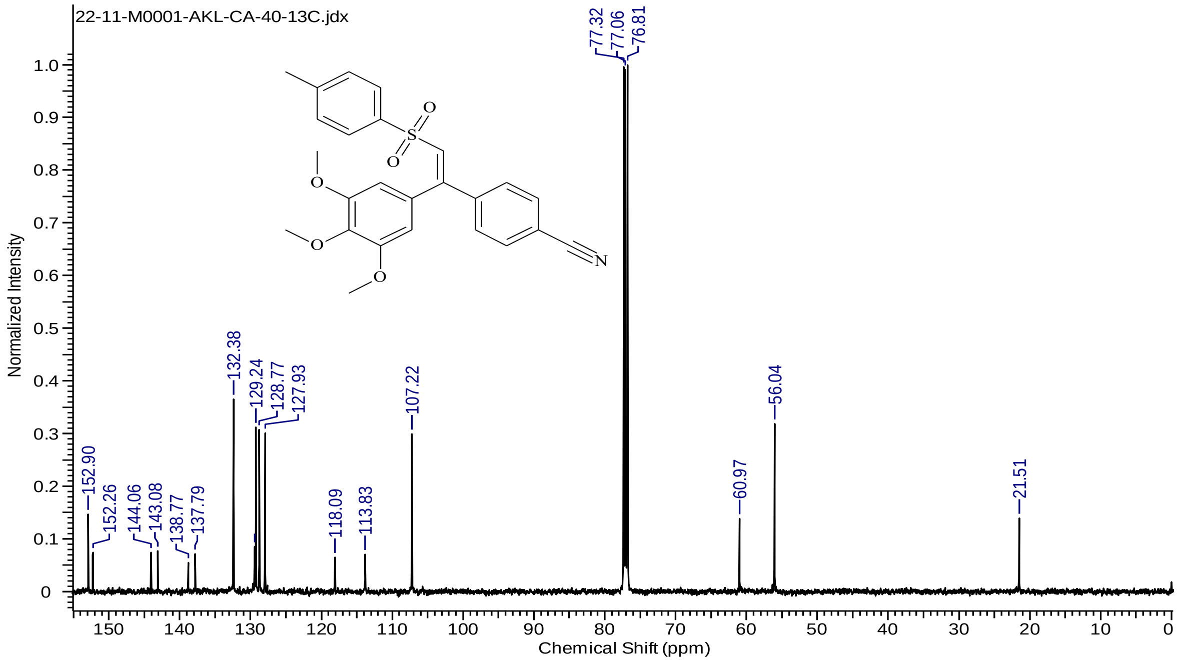

19. 4Q, $C_{25}H_{22}F_4O_5S$, (E)-5-(1-(2-fluoro-3-(trifluoromethyl)phenyl)-2-tosylvinyl)-1,2,3-trimethoxybenzene

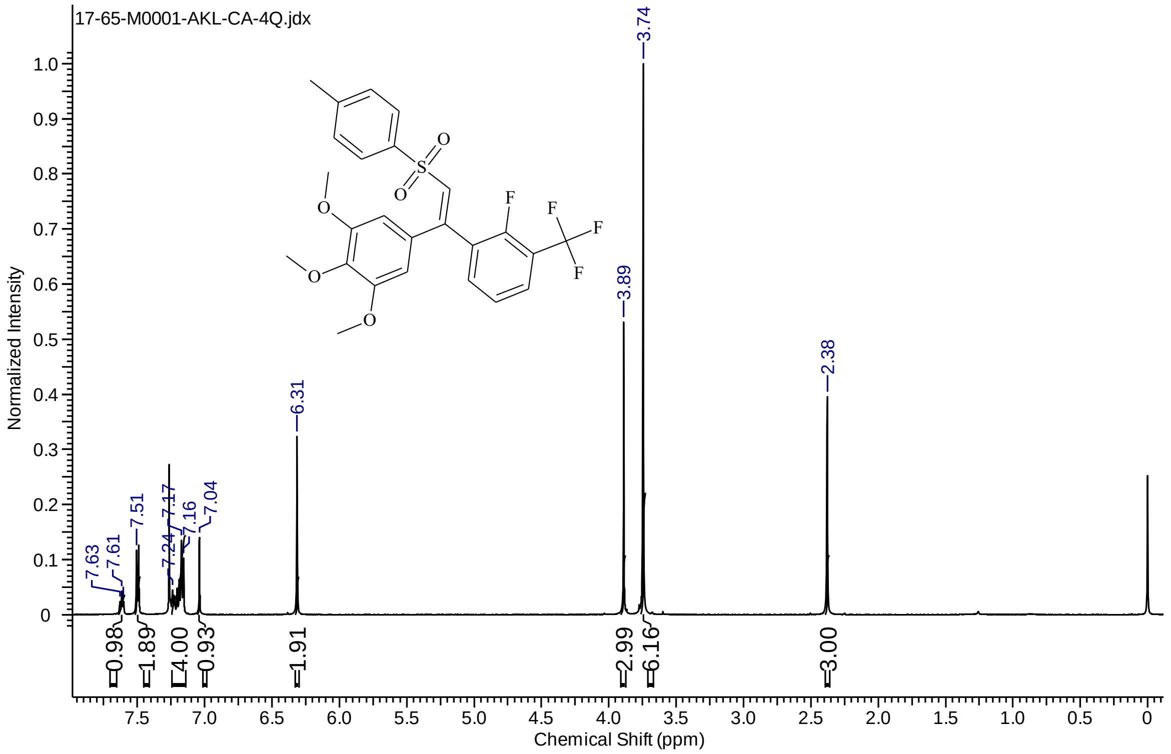

20. 4R, $C_{25}H_{25}FO_5S$, (E)-5-(1-(2-fluoro-5-methylphenyl)-2-tosylvinyl)-1,2,3-Trimethoxybenzene

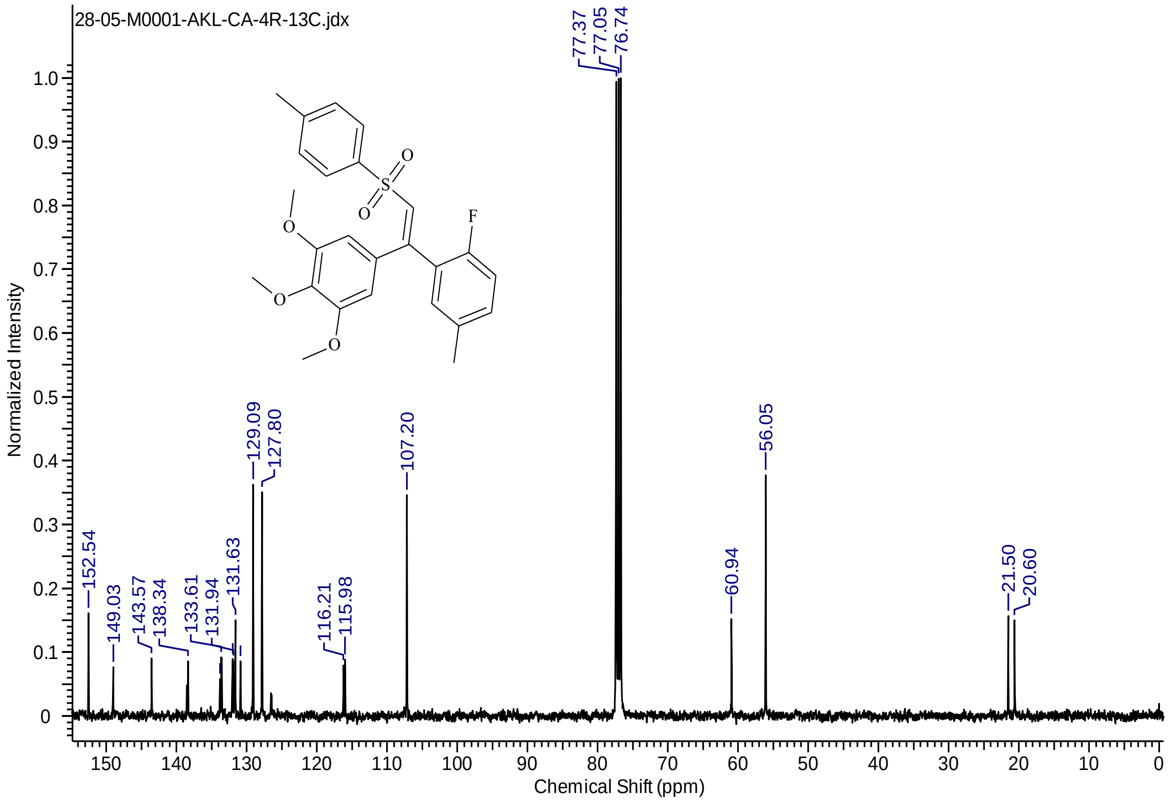

21. 4T, $C_{25}H_{25}FO_5S$, (E)-5-(1-(3-fluoro-4-methylphenyl)-2-tosylvinyl)-1,2,3-trimethoxybenzene

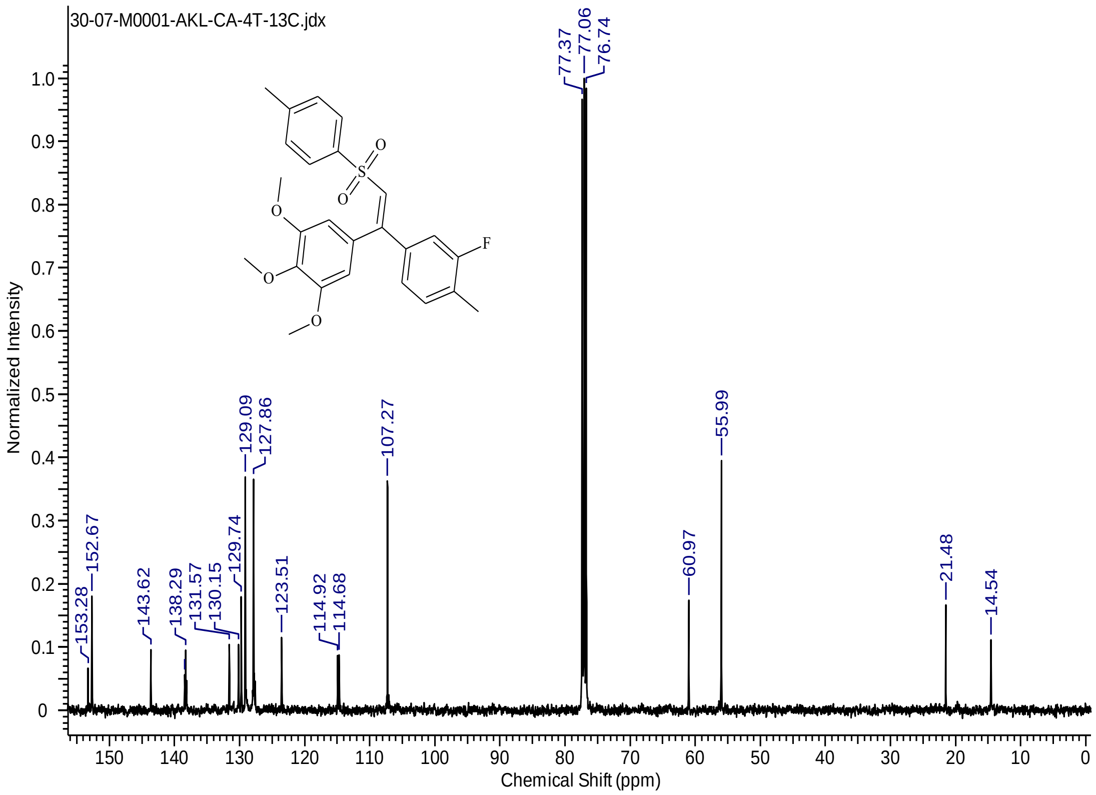

22. 4U, $C_{30}H_{28}O_5S$, (Z)-4-(2-tosyl-1-(3,4,5-trimethoxyphenyl)vinyl)-1,1'-biphenyl

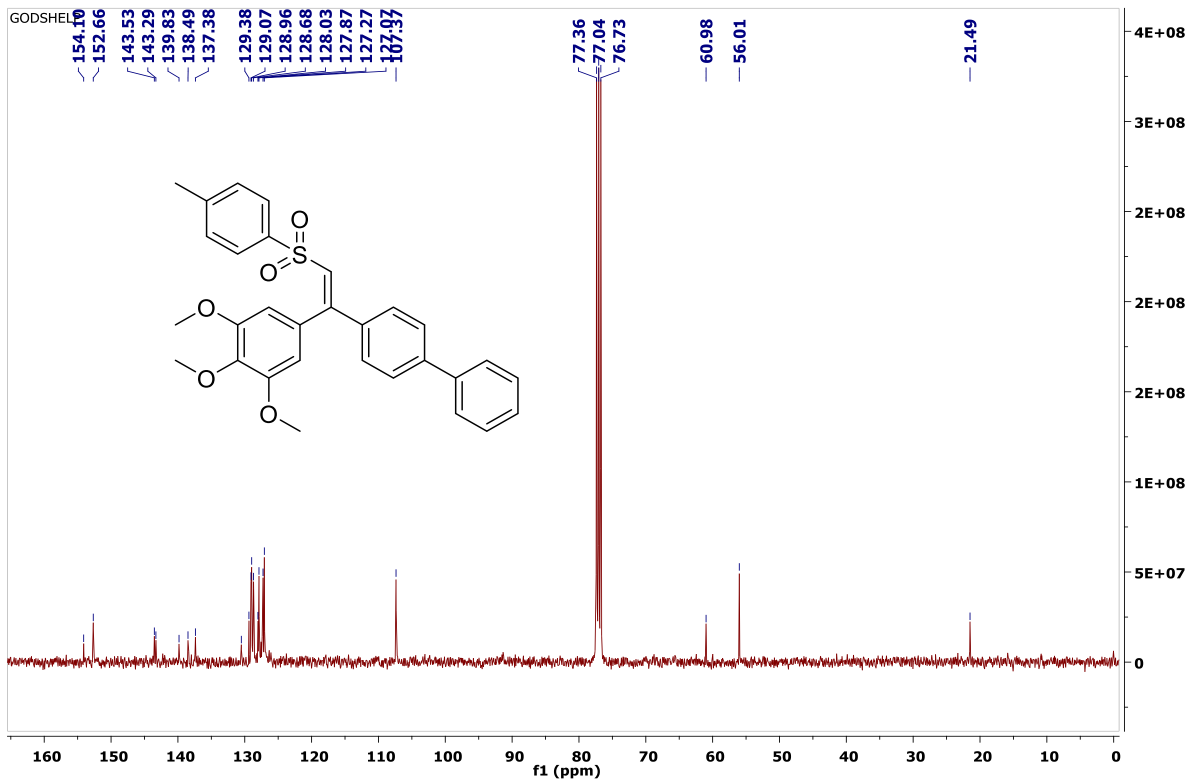

23. 4V, $C_{18}H_{18}O_5S$, 4-methylphenyl (3,4,5-trimethoxyphenyl)ethynyl sulfone

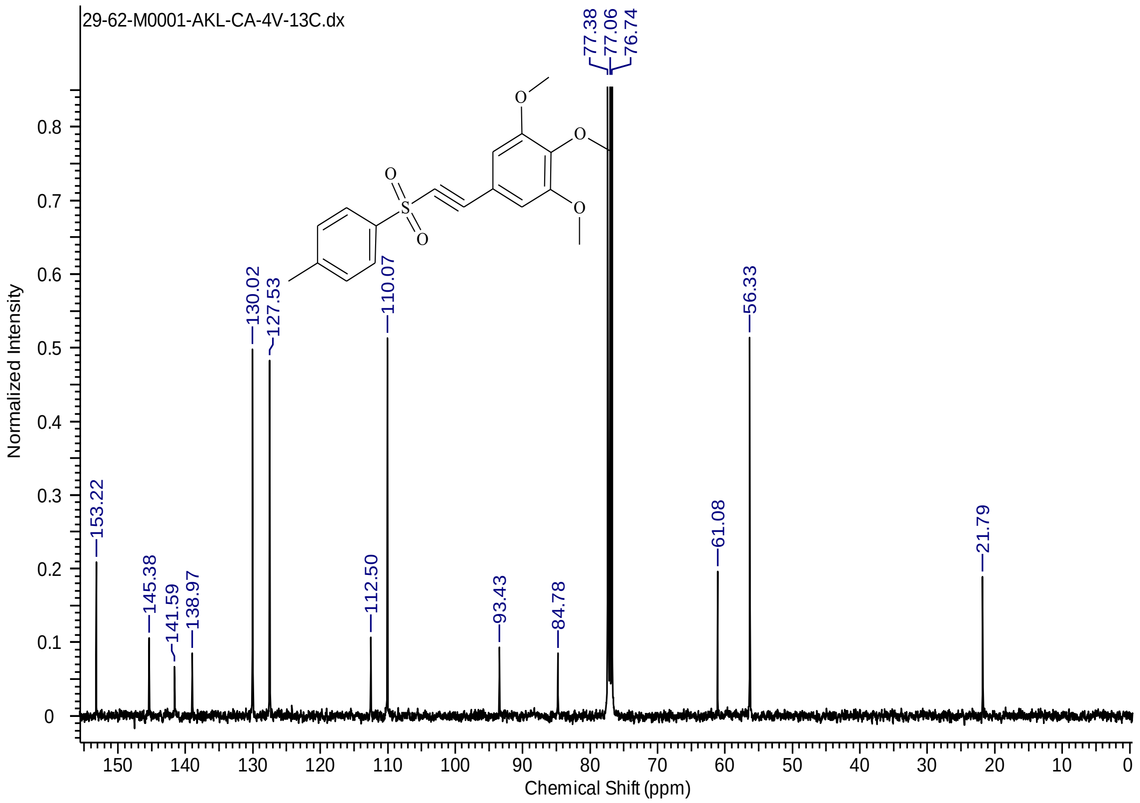

MS SPECTRA OF THE SYNTHESIZED COMPOUNDS

1. $C_{11}H_{12}Br_2O_3$; 5-(2,2-dibromovinyl)-1,2,3-trimethoxybenzene

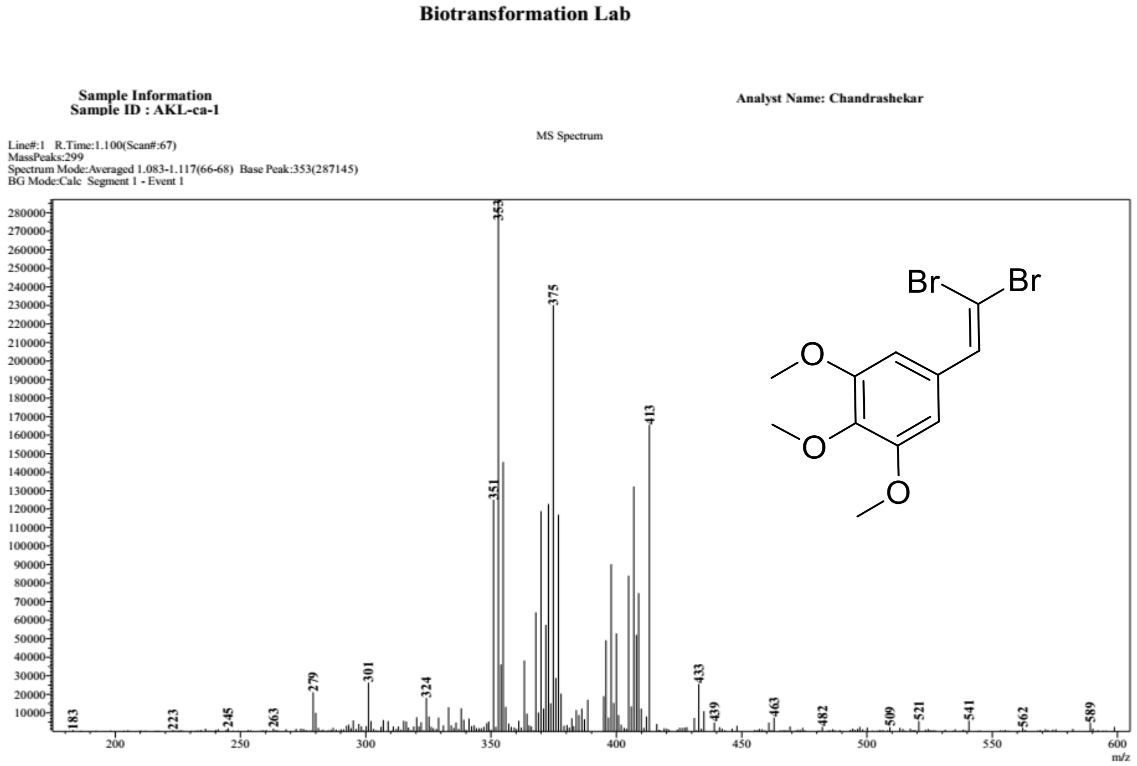

2. $C_{11}H_{12}O_3$, 5-ethynyl-1,2,3-trimethoxybenzene

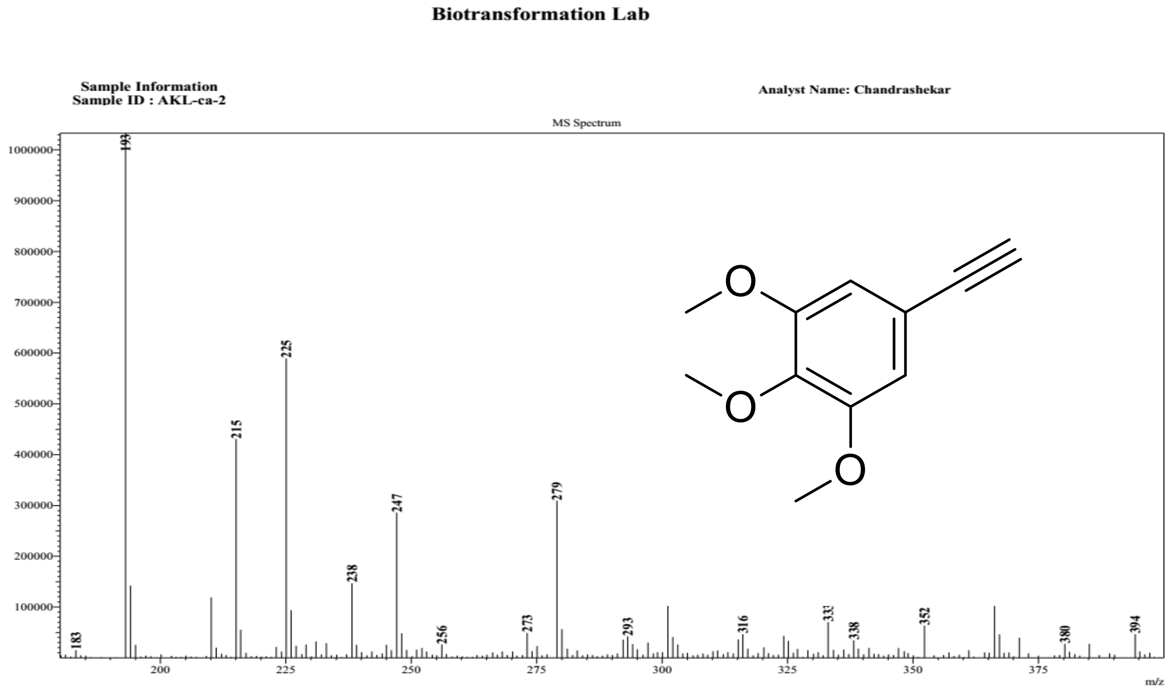

3. $C_{18}H_{19}IO_5S$, (E)-5-(1-iodo-2-tosylvinyl)-1,2,3-trimethoxybenzene

Biotransformation Lab

Sample Information
Sample ID : AKL-ca3b

Analyst Name: Chandrashekar

MS Spectrum

Line#:1 R.Time:1.050(Scan#:64)
MassPeaks:255
Spectrum Mode:Averaged 1.033-1.067(63-65) Base Peak:497(3771446)
BG Mode:Calc Segment 1 - Event 1

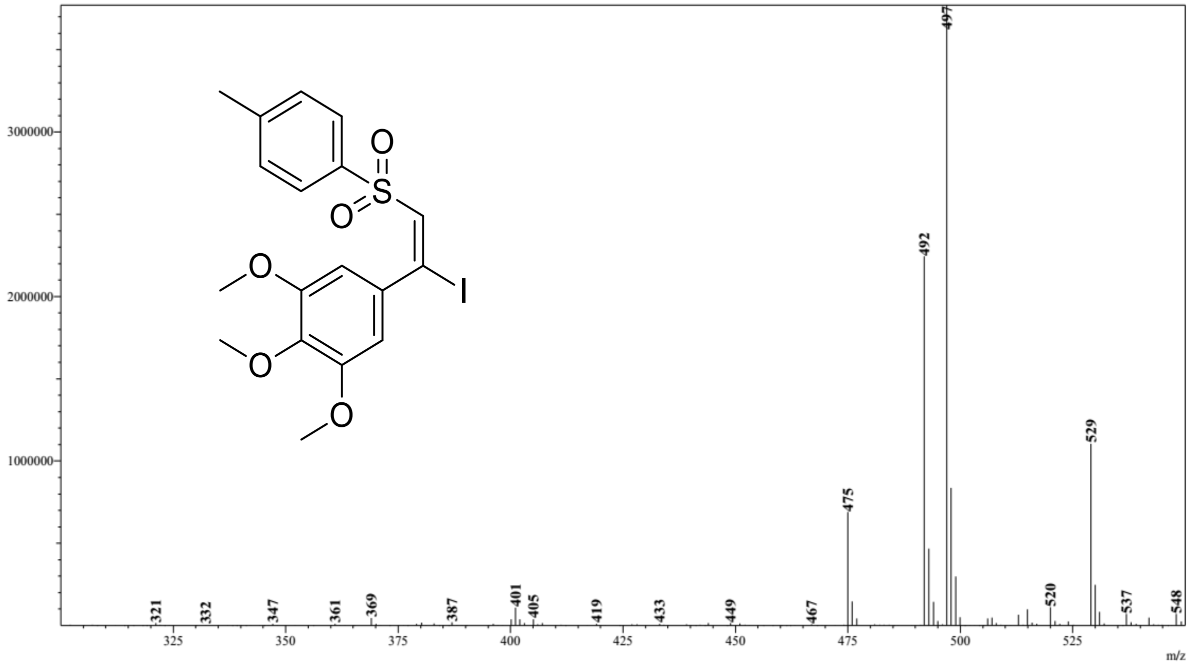

4. 4A, $C_{24}H_{24}O_5S$, (Z)-1,2,3-trimethoxy-5-(1-phenyl-2-tosylvinyl)benzene

Biotransformation Lab

Sample Information
Sample ID : AKL-ca4a

Analyst Name: Chandrashekar

MS Spectrum

Line#:1 R.Time:0.800(Scan#:49)
MassPeaks:245
Spectrum Mode:Averaged 0.767-0.833(47-51) Base Peak:562(1381693)
BG Mode:Calc Segment 1 - Event 1

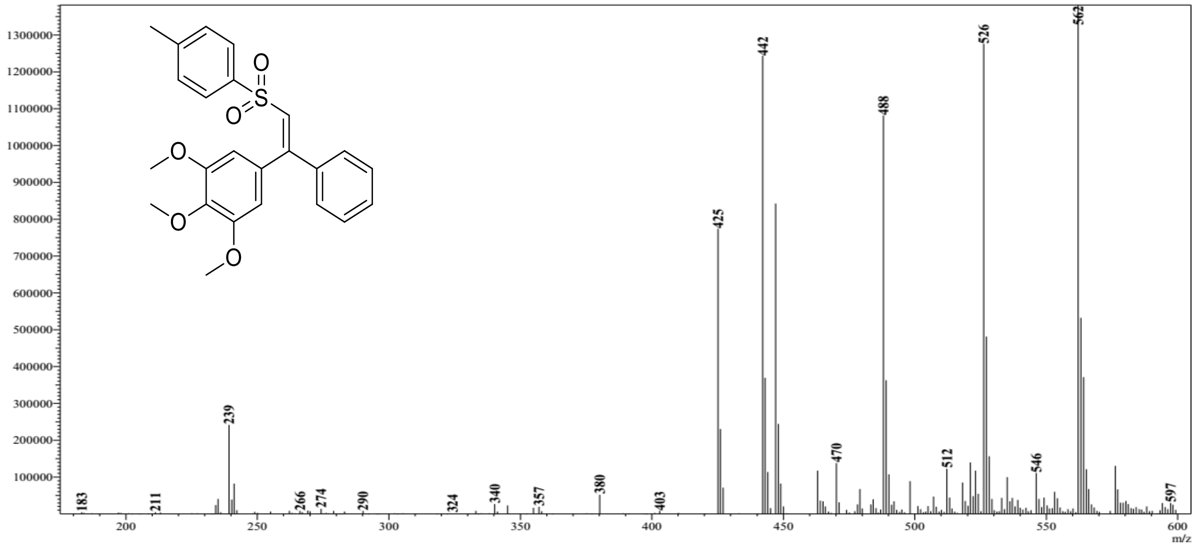

5. 4B, (Z)-1,2,3-trimethoxy-5-(1-(4-methoxyphenyl)-2-tosylvinyl)benzene

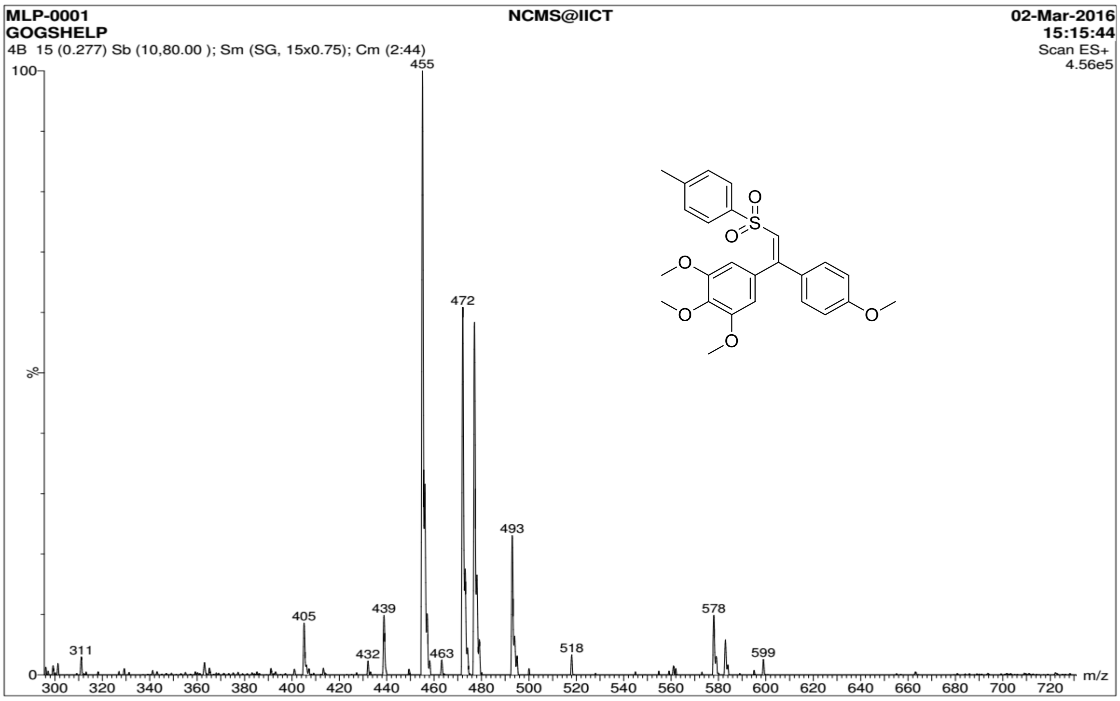

6. 4C, $C_{26}H_{28}O_7S$, (E)-5-(1-(2,4-dimethoxyphenyl)-2-tosylvinyl)-1,2,3-trimethoxybenzene

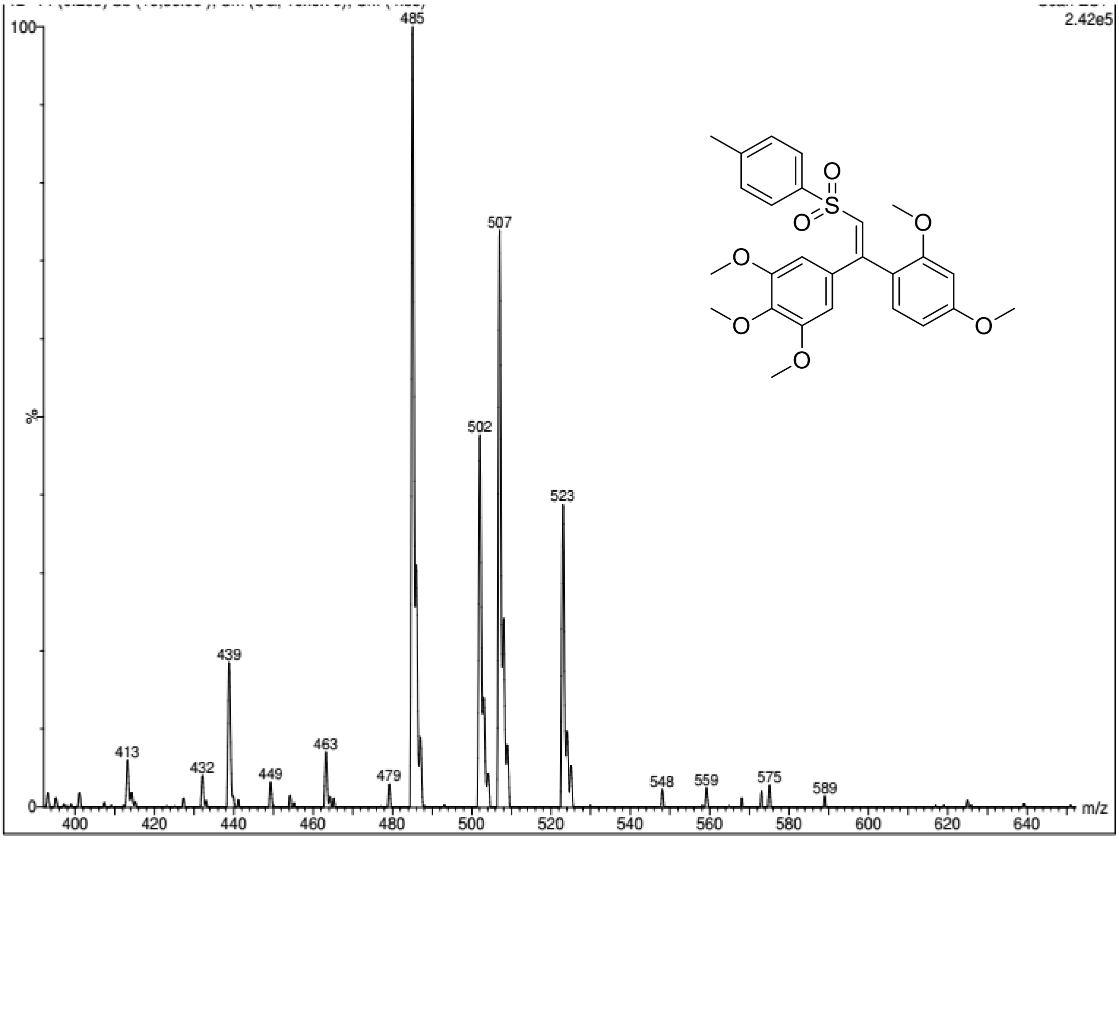

7. 4D, $C_{26}H_{28}O_7S$, (Z)-5-(1-(3,4-dimethoxyphenyl)-2-tosylvinyl)-1,2,3-trimethoxybenzene

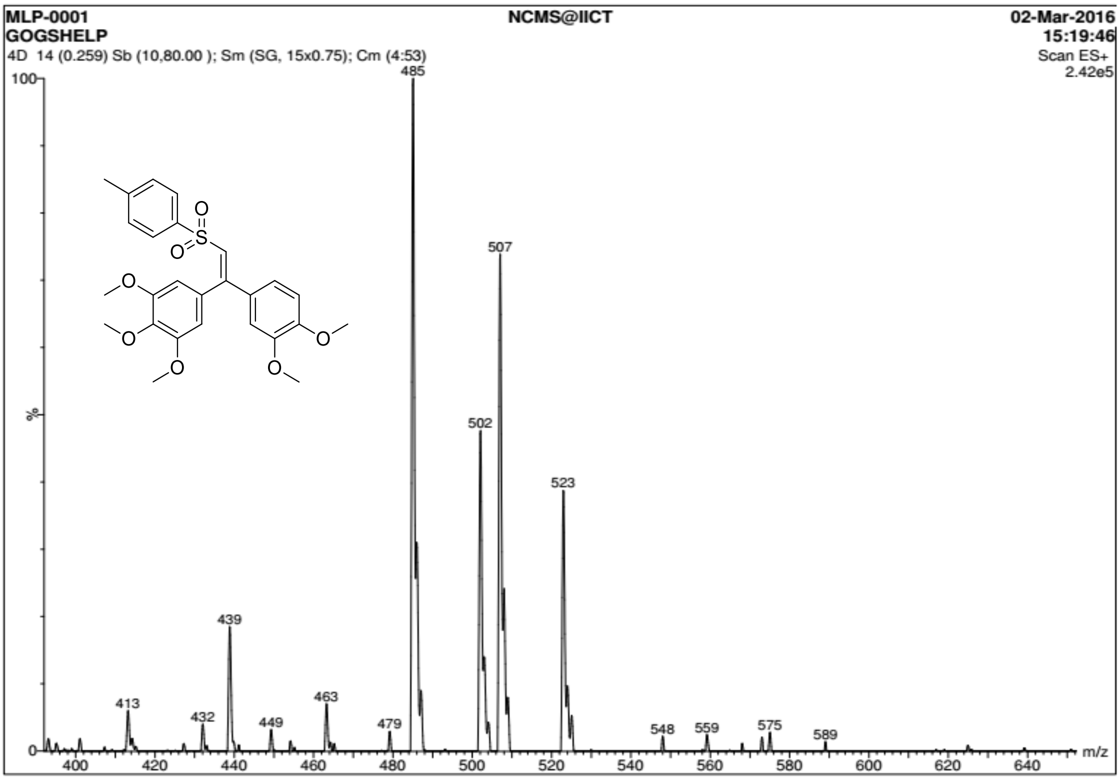

8. 4E, $C_{26}H_{28}O_7S$, (Z)-5-(1-(3,5-dimethoxyphenyl)-2-tosylvinyl)-1,2,3-trimethoxybenzene

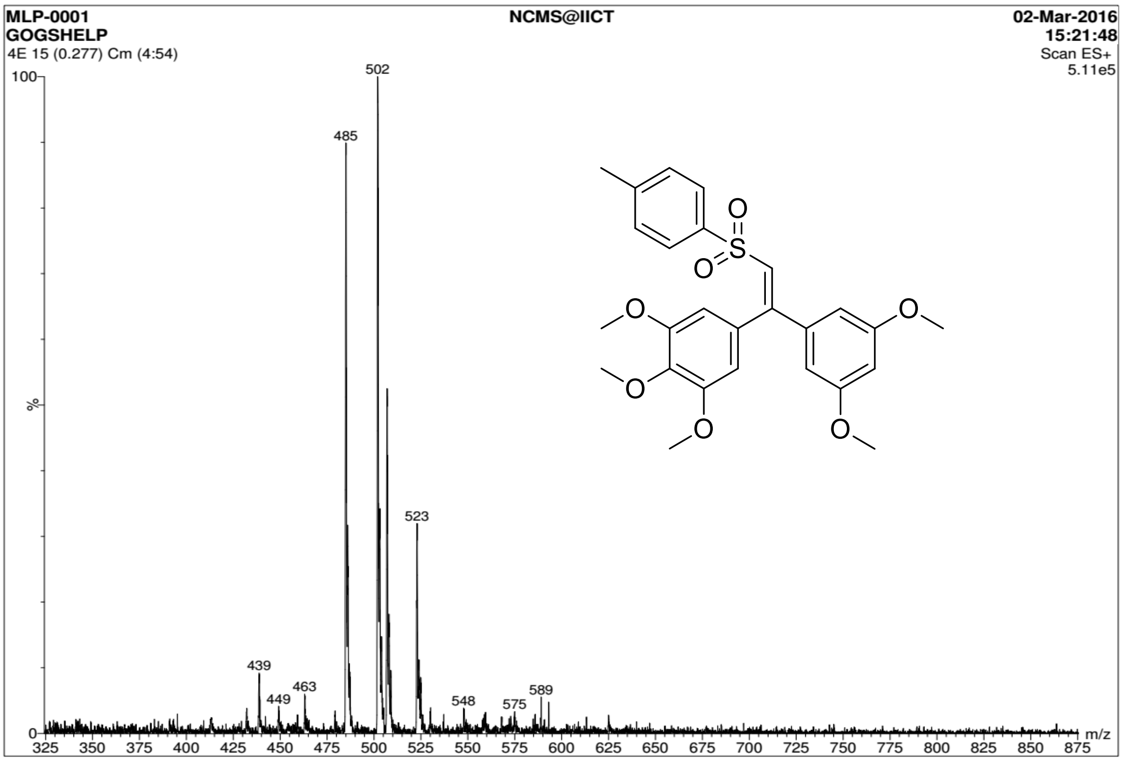

9. 4F, $C_{18}H_{18}O_5S$, 4-methylphenyl (3,4,5-trimethoxyphenyl)ethynyl sulfone

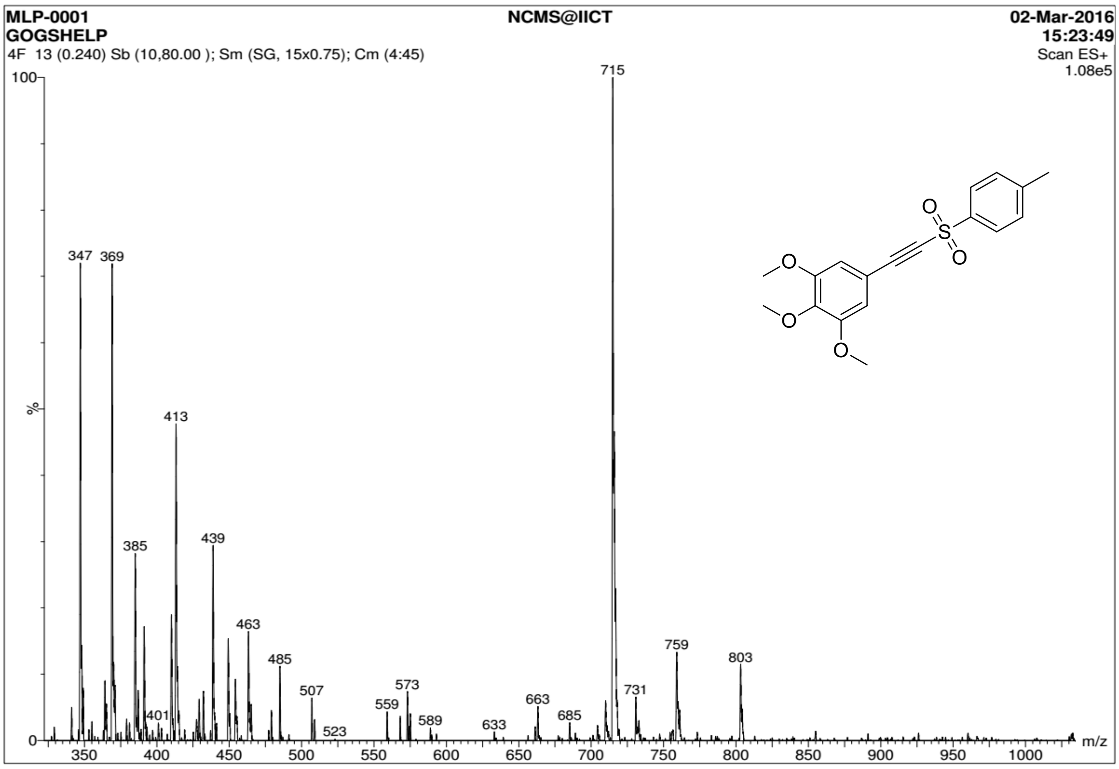

10. 4G, $C_{24}H_{23}FO_5S$, (Z)-5-(1-(4-fluorophenyl)-2-tosylvinyl)-1,2,3-trimethoxybenzene

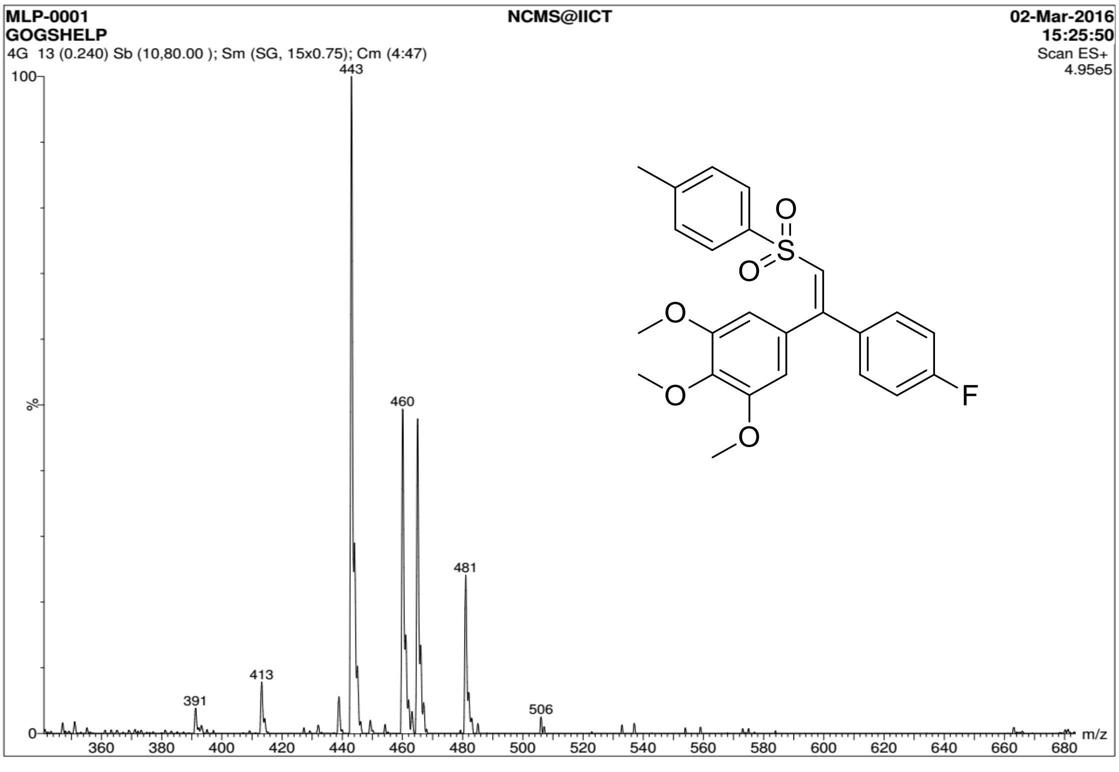

11. 4H, $C_{25}H_{23}F_3O_5S$, (Z)-1,2,3-trimethoxy-5-(2-tosyl-1-(4-(trifluoromethyl)phenyl)vinyl)benzene

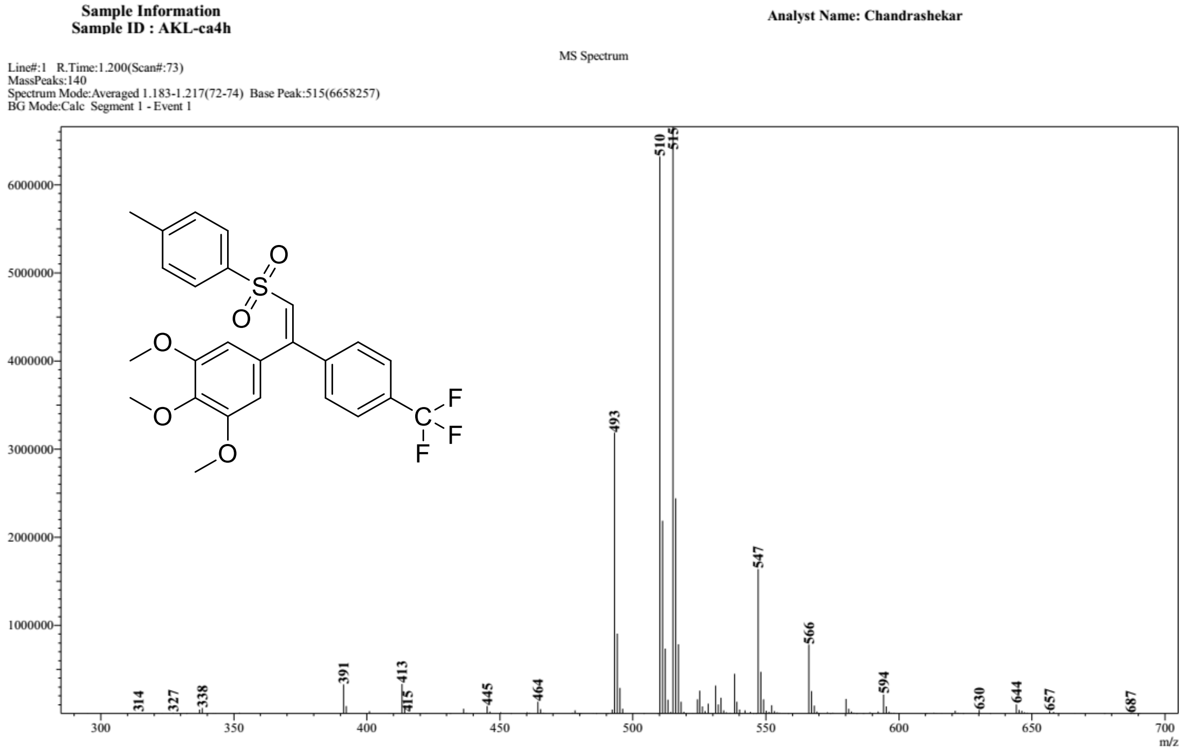

12. 4I, $C_{24}H_{22}F_2O_5S$, (E)-5-(1-(2,4-difluorophenyl)-2-tosylvinyl)-1,2,3-trimethoxybenzene

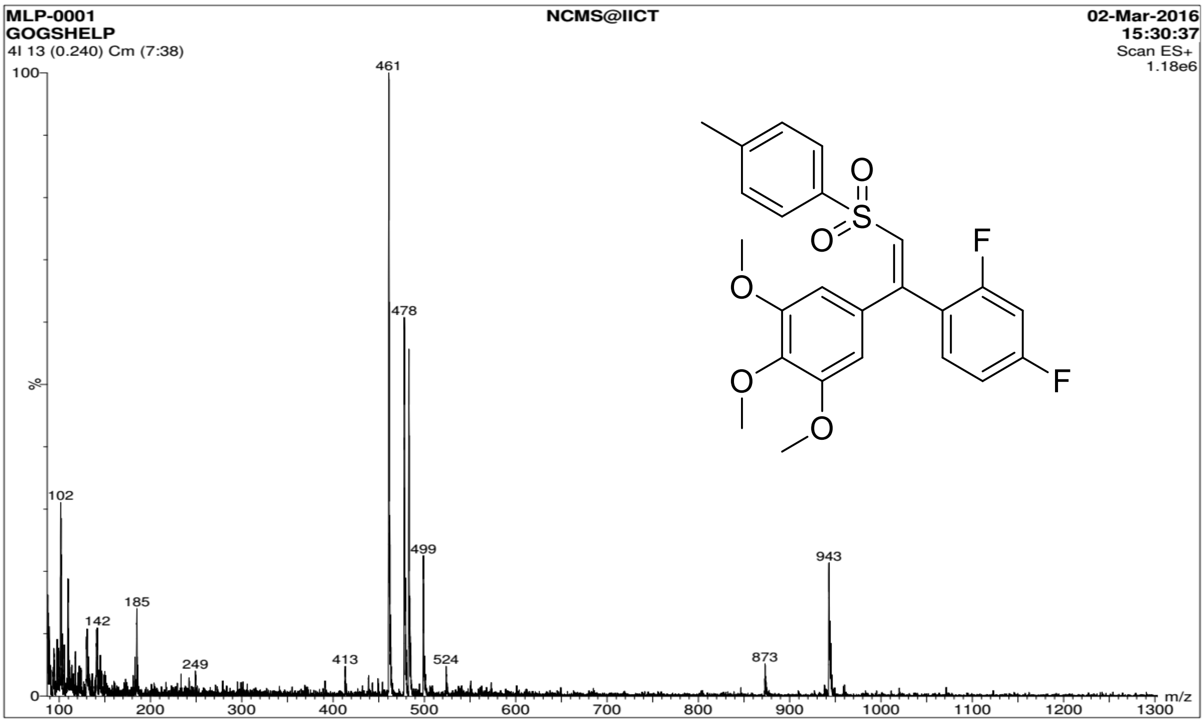

13. 4J,$C_{24}H_{22}F_2O_5S$, (E)-5-(1-(3,4-difluorophenyl)-2-tosylvinyl)-1,2,3-trimethoxybenzene

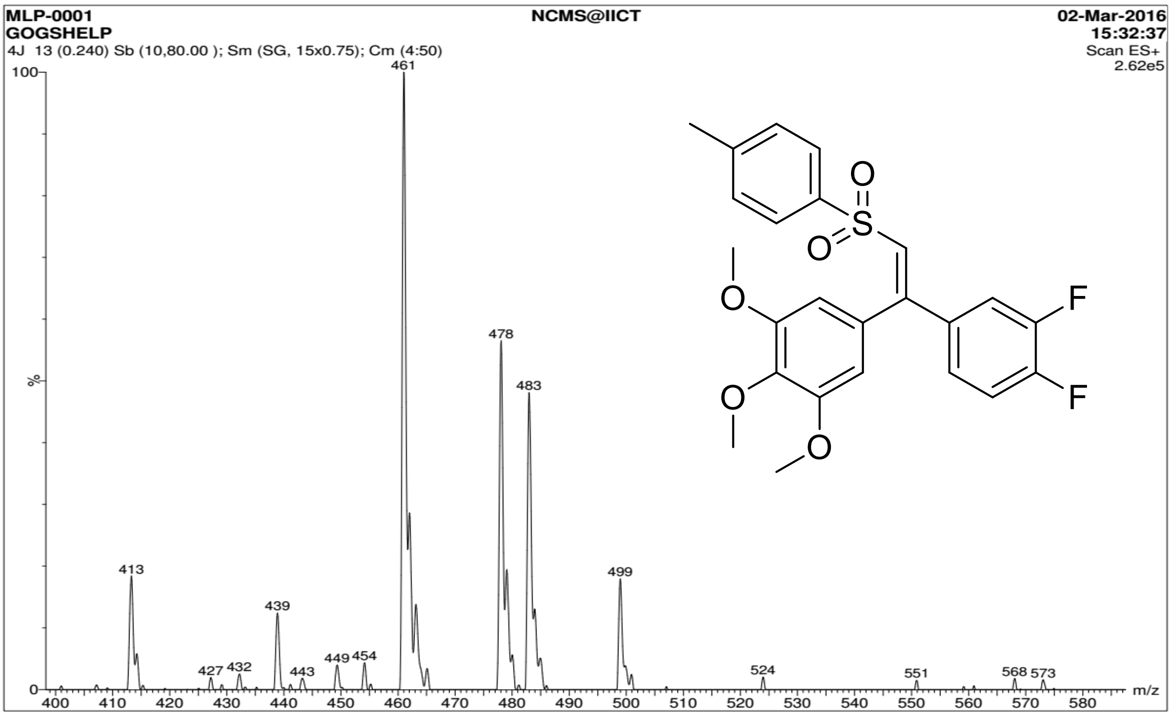

14. 4K, $C_{26}H_{27}FO_6S$, (E)-5-(1-(5-ethoxy-2-fluorophenyl)-2-tosylvinyl)-1,2,3-trimethoxybenzene

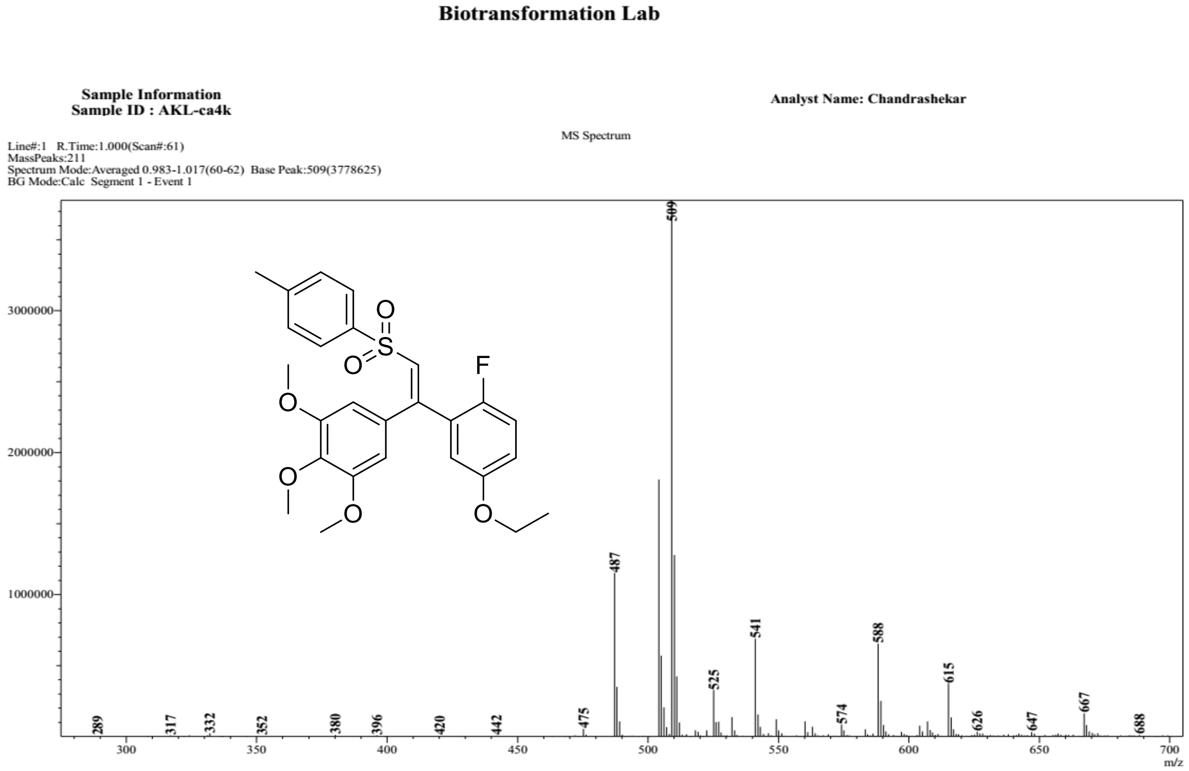

15. 4L, $C_{24}H_{22}ClFO_5S$, (E)-5-(1-(3-chloro-4-fluorophenyl)-2-tosylvinyl)-1,2,3-trimethoxybenzene

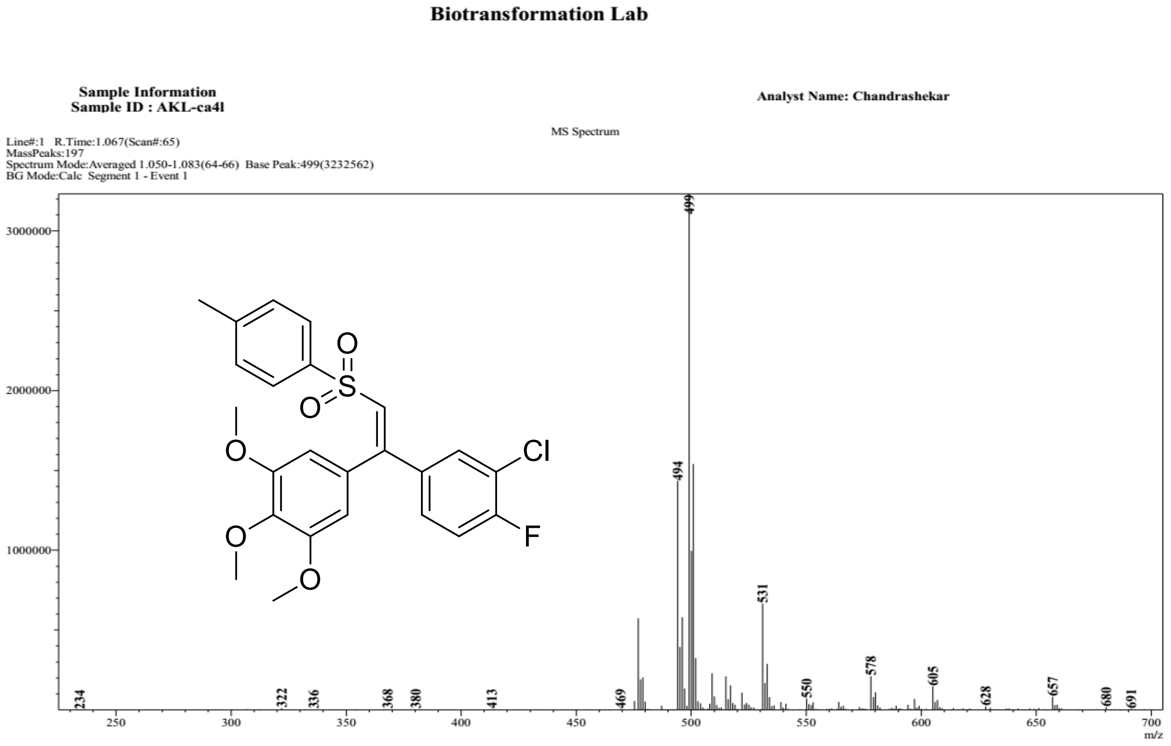

16. 4M, $C_{24}H_{23}ClO_5S$, (Z)-5-(1-(4-chlorophenyl)-2-tosylvinyl)-1,2,3-trimethoxybenzene

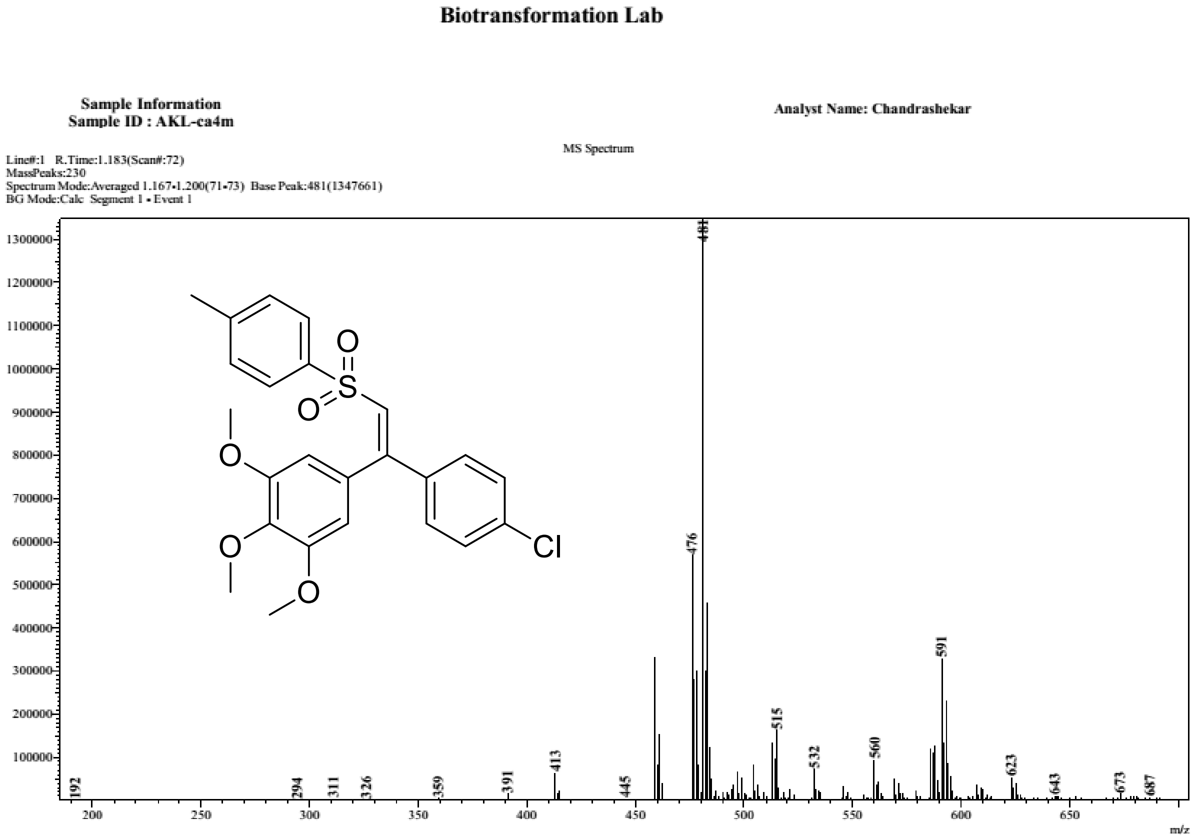

17. 4N, $C_{24}H_{22}Cl_2O_5S$, (E)-5-(1-(3,4-dichlorophenyl)-2-tosylvinyl)-1,2,3-trimethoxybenzene

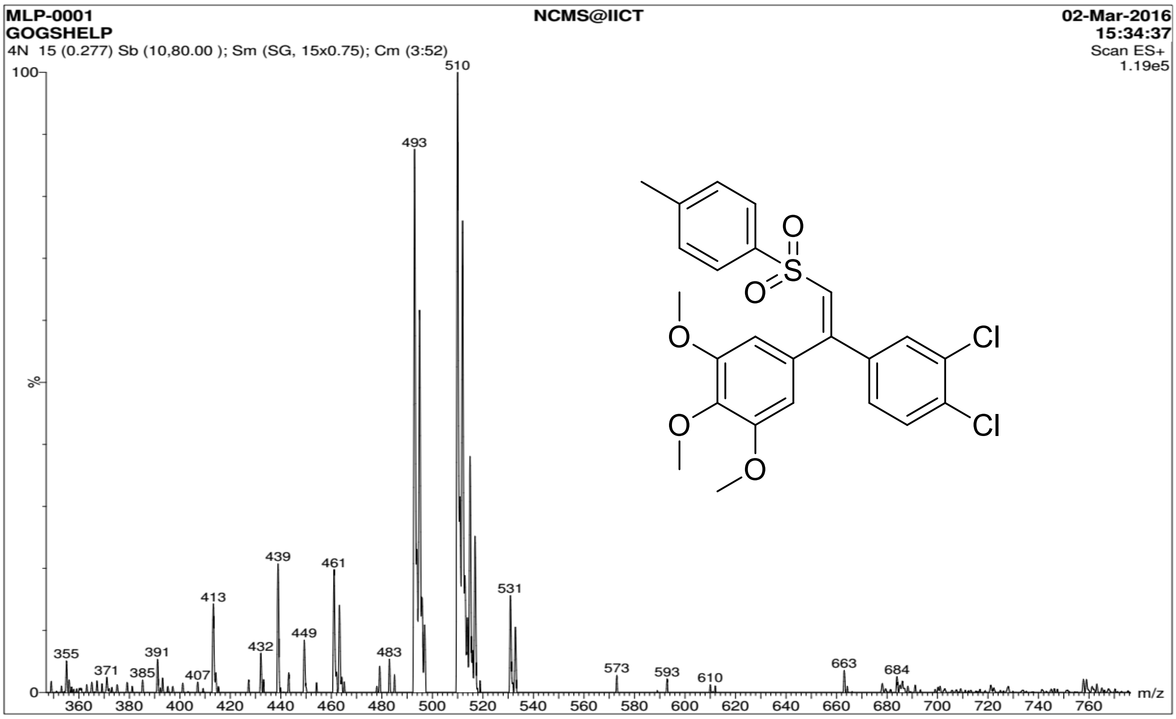

18. 4O, $C_{25}H_{23}NO_5S$, (Z)-4-(2-tosyl-1-(3,4,5trimethoxyphenyl)vinyl) benzonitrile

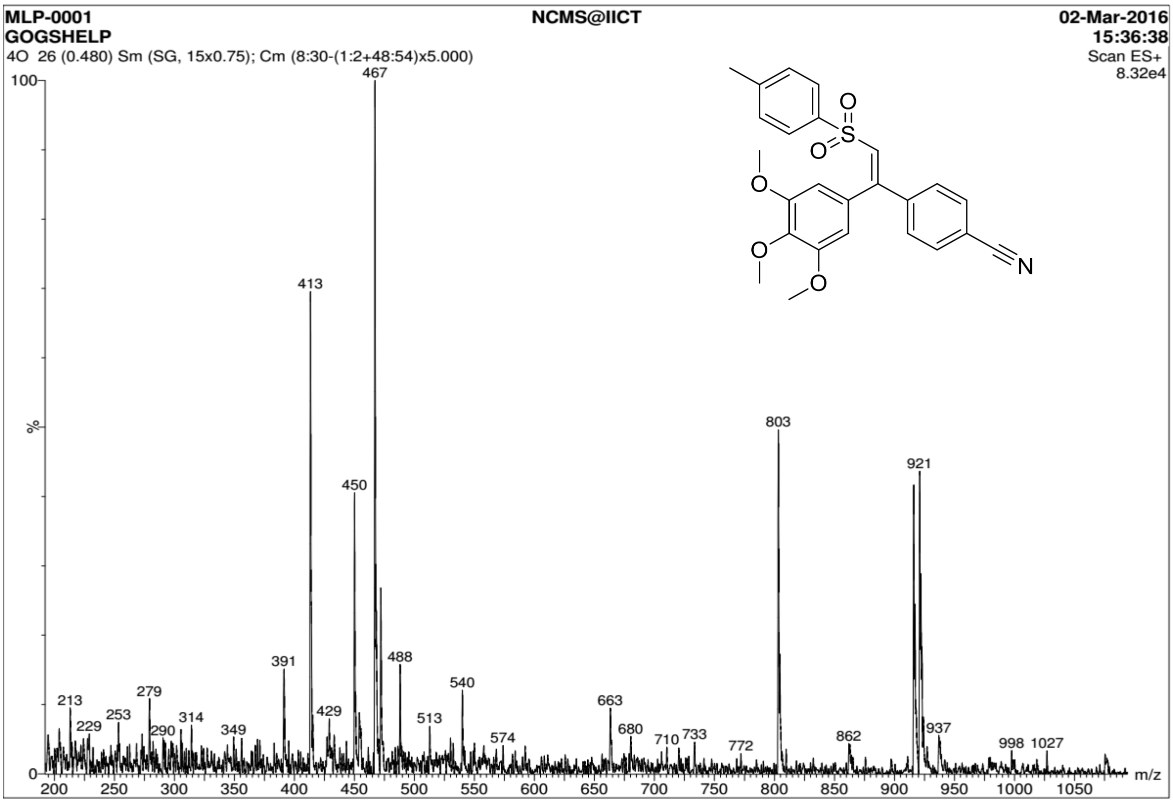

19. 4Q, $C_{25}H_{22}F_4O_5S$, (E)-5-(1-(2-fluoro-3-(trifluoromethyl)phenyl)-2-tosylvinyl)-1,2,3-trimethoxybenzene

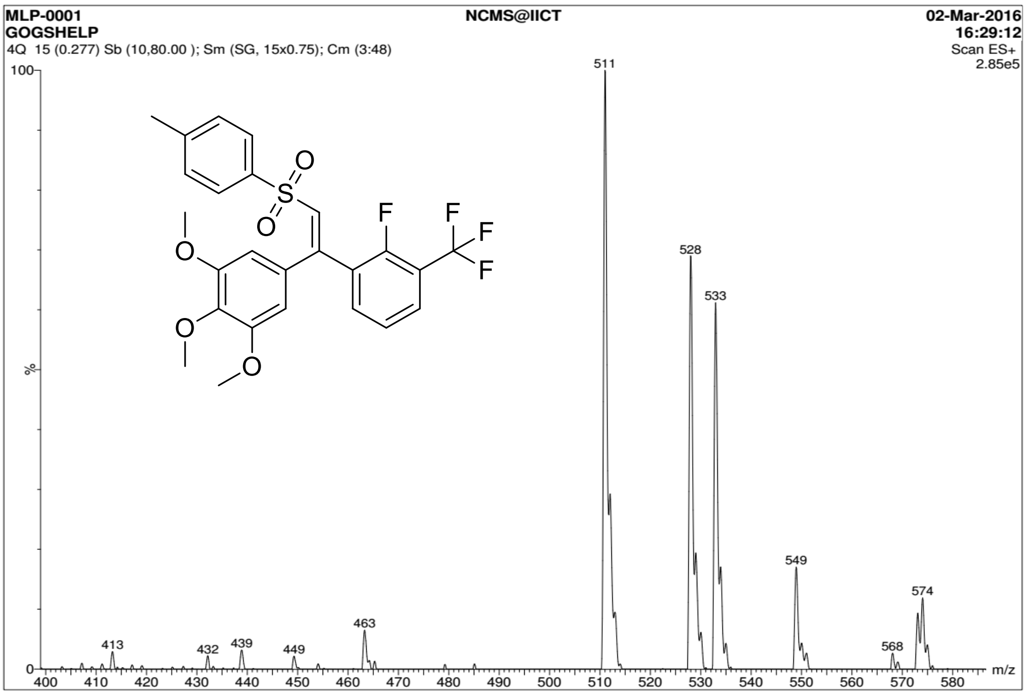

20. 4R, $C_{25}H_{25}FO_5S$, (E)-5-(1-(2-fluoro-5-methylphenyl)-2-tosylvinyl)-1,2,3-

Trimethoxybenzene

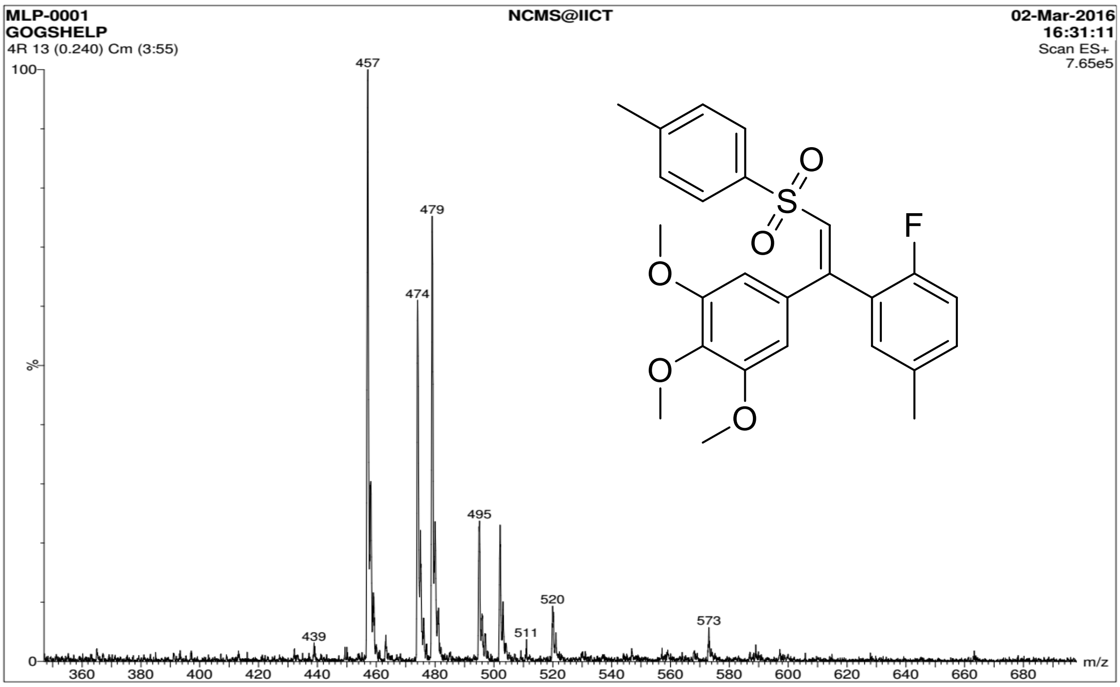

21. 4T, $C_{25}H_{25}FO_5S$, (E)-5-(1-(3-fluoro-4-methylphenyl)-2-tosylvinyl)-1,2,3-trimethoxybenzene

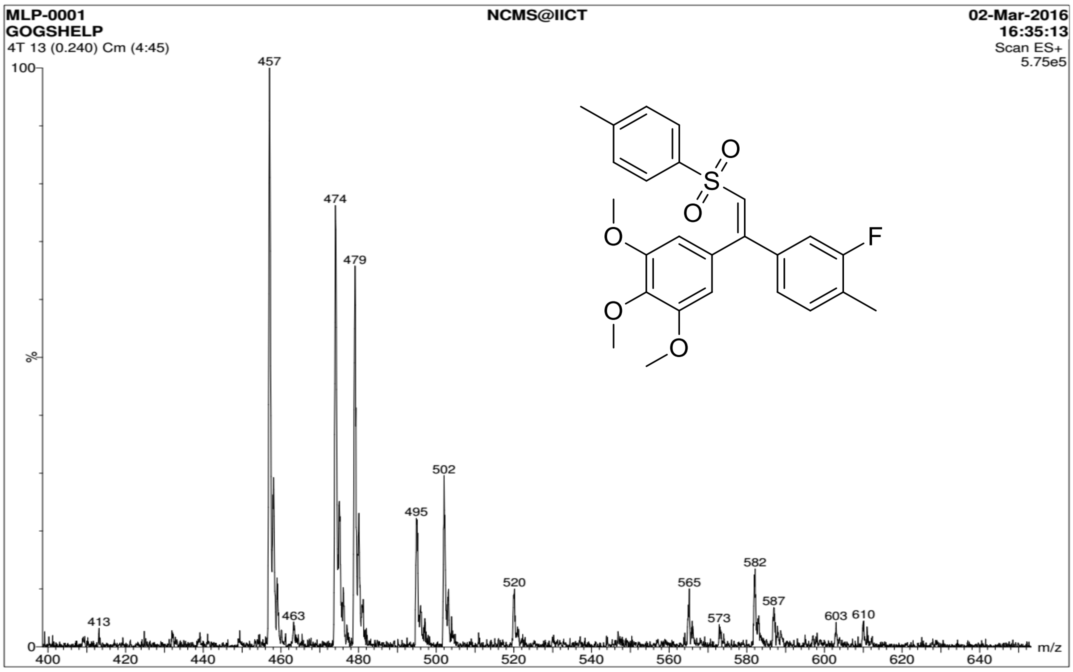

22. 4U, $C_{30}H_{28}O_5S$, (Z)-4-(2-tosyl-1-(3,4,5-trimethoxyphenyl)vinyl)-1,1'-biphenyl

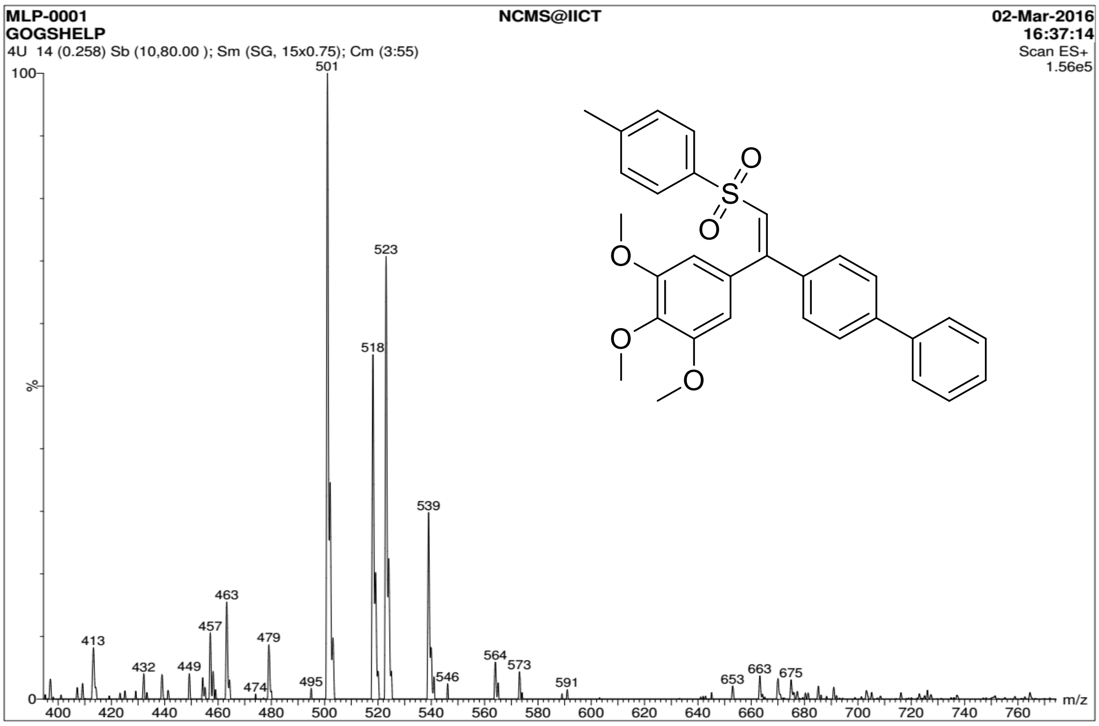

23. 4V, $C_{18}H_{18}O_5S$, 4-methylphenyl (3,4,5-trimethoxyphenyl)ethynyl sulfone

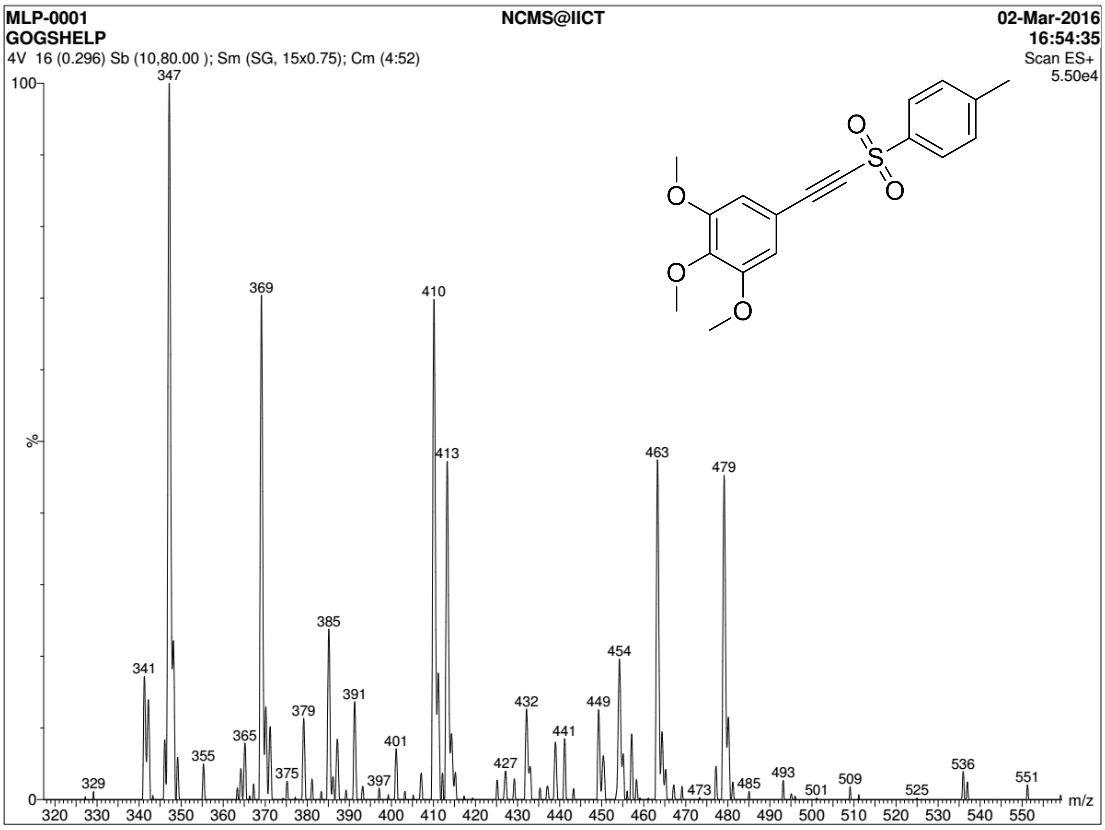

HRMS SPECTRA OF SYNTHESIZED COMPOUNDS

1. $C_{18}H_{19}IO_5S$, (E)-5-(1-iodo-2-tosylvinyl)-1,2,3-trimethoxybenzene

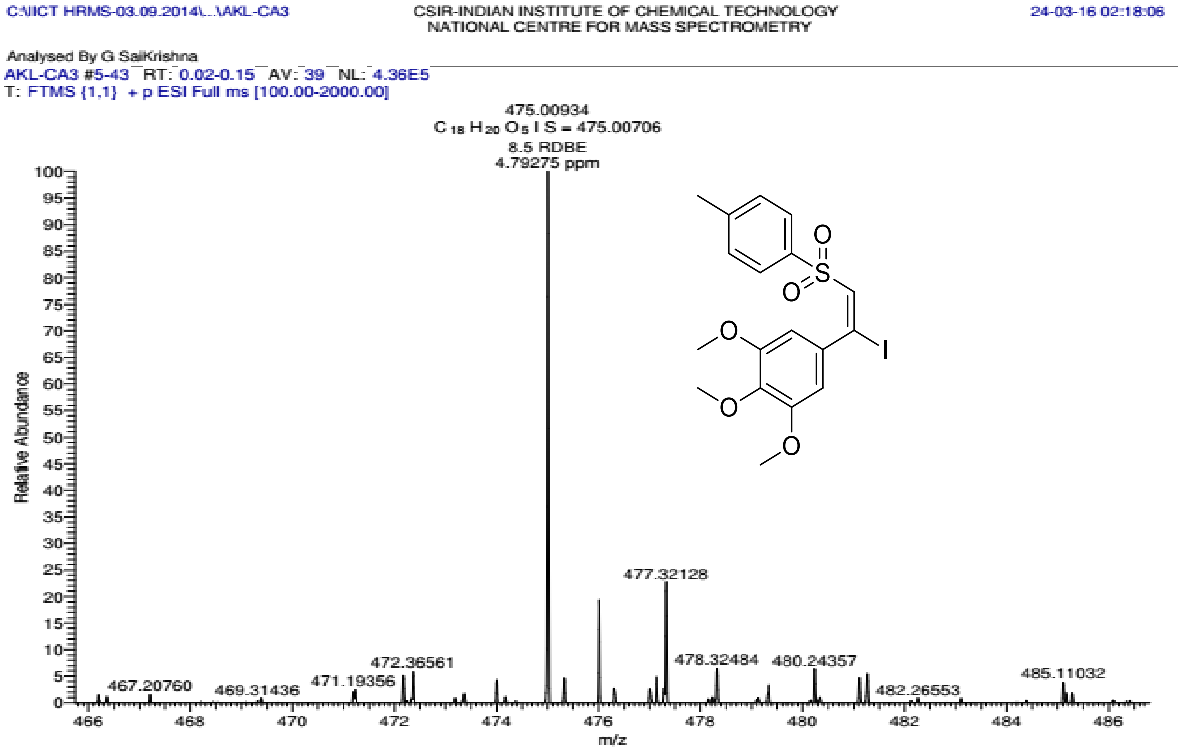

2. 4A, $C_{24}H_{24}O_5S$, (Z)-1,2,3-trimethoxy-5-(1-phenyl-2-tosylvinyl)benzene

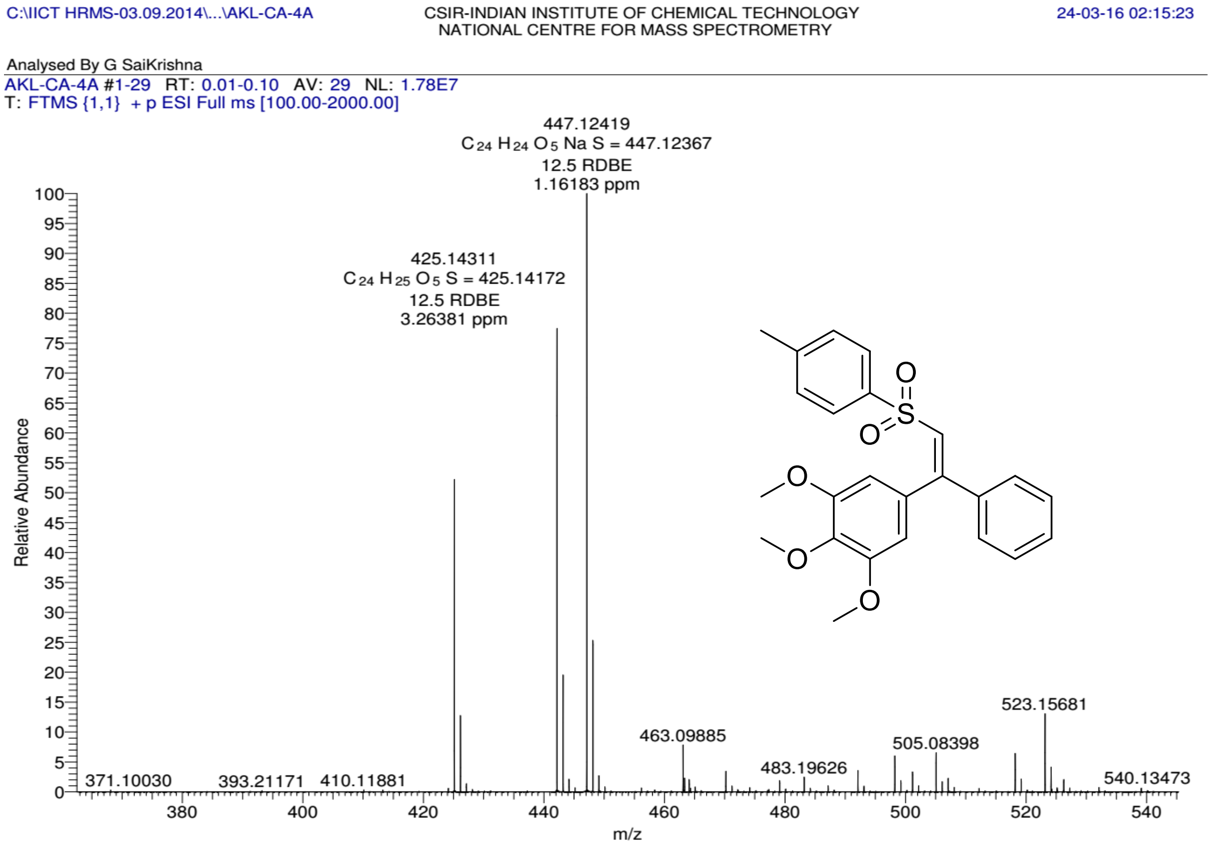

3. 4B, (Z)-1,2,3-trimethoxy-5-(1-(4-methoxyphenyl)-2-tosylvinyl)benzene

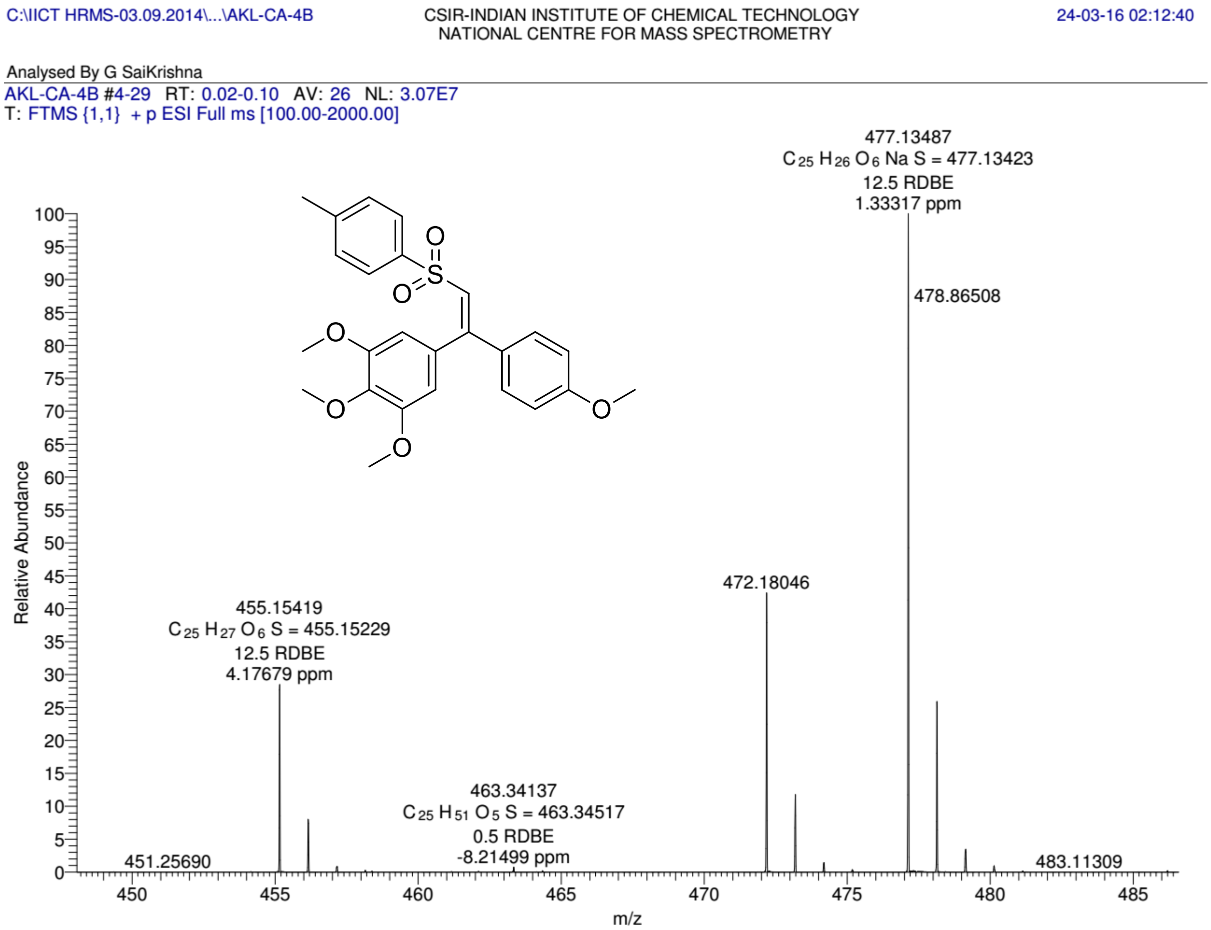

4. 4C, $C_{26}H_{28}O_7S$, (E)-5-(1-(2,4-dimethoxyphenyl)-2-tosylvinyl)-1,2,3-trimethoxybenzene

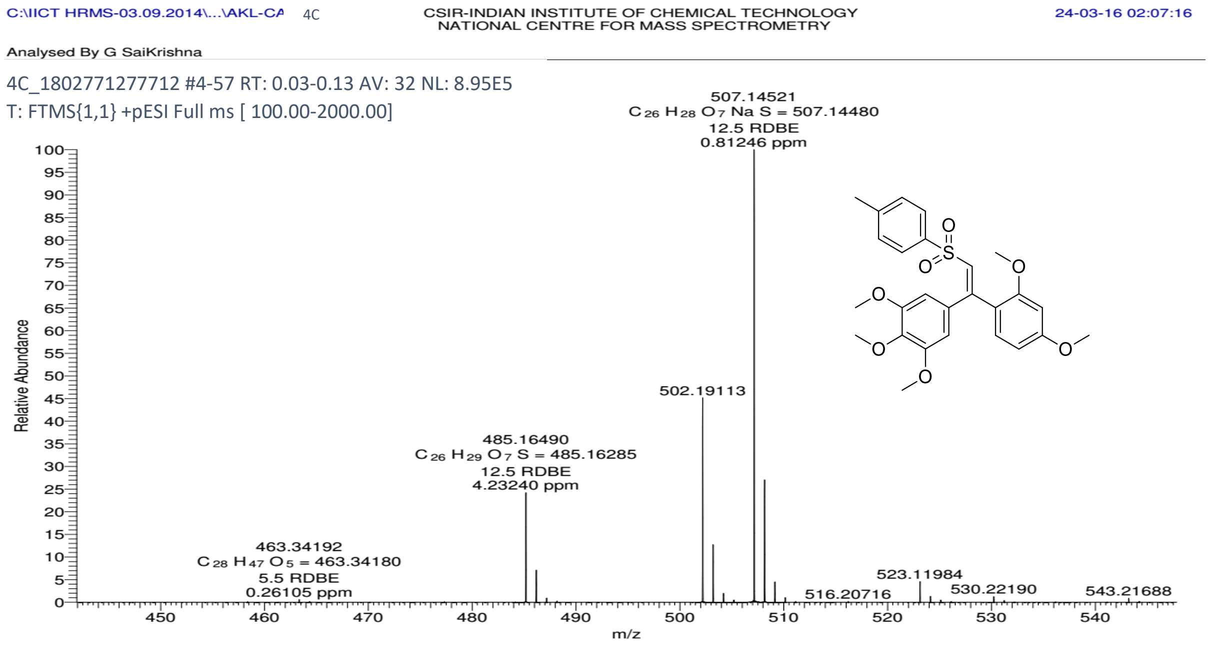

5. 4D, $C_{26}H_{28}O_7S$, (Z)-5-(1-(3,4-dimethoxyphenyl)-2-tosylvinyl)-1,2,3-trimethoxybenzene

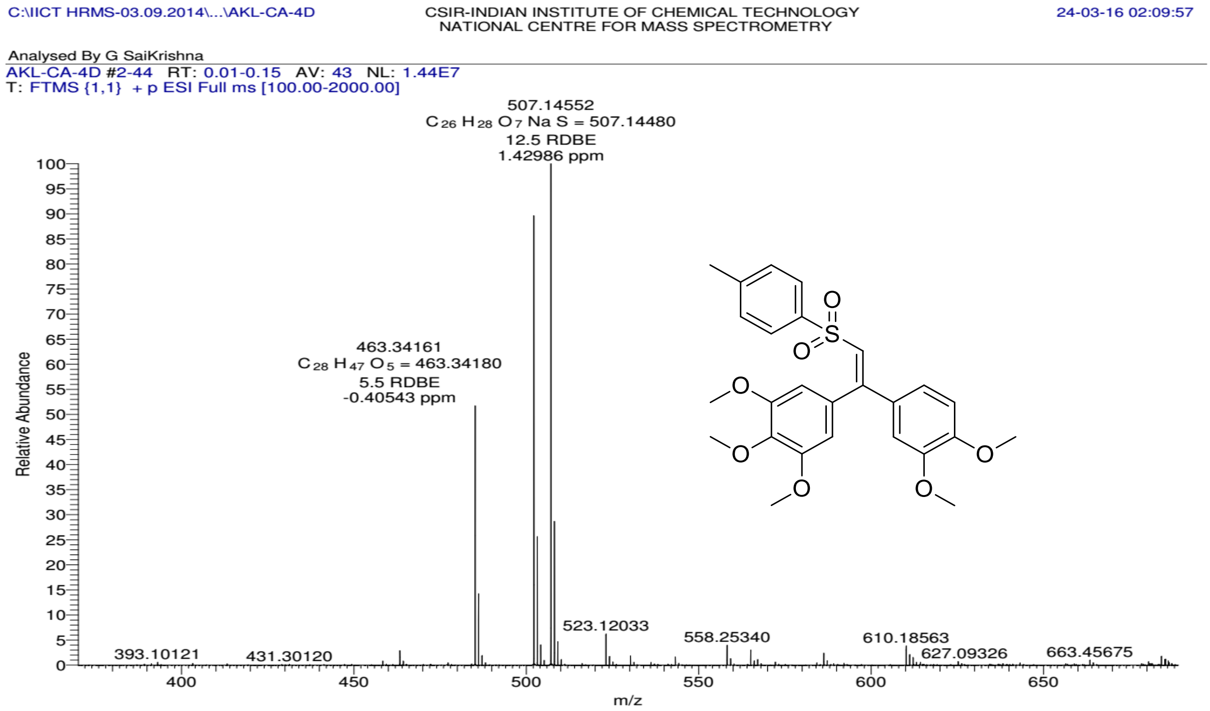

6. 4E, $C_{26}H_{28}O_7S$, (Z)-5-(1-(3,5-dimethoxyphenyl)-2-tosylvinyl)-1,2,3-trimethoxybenzene

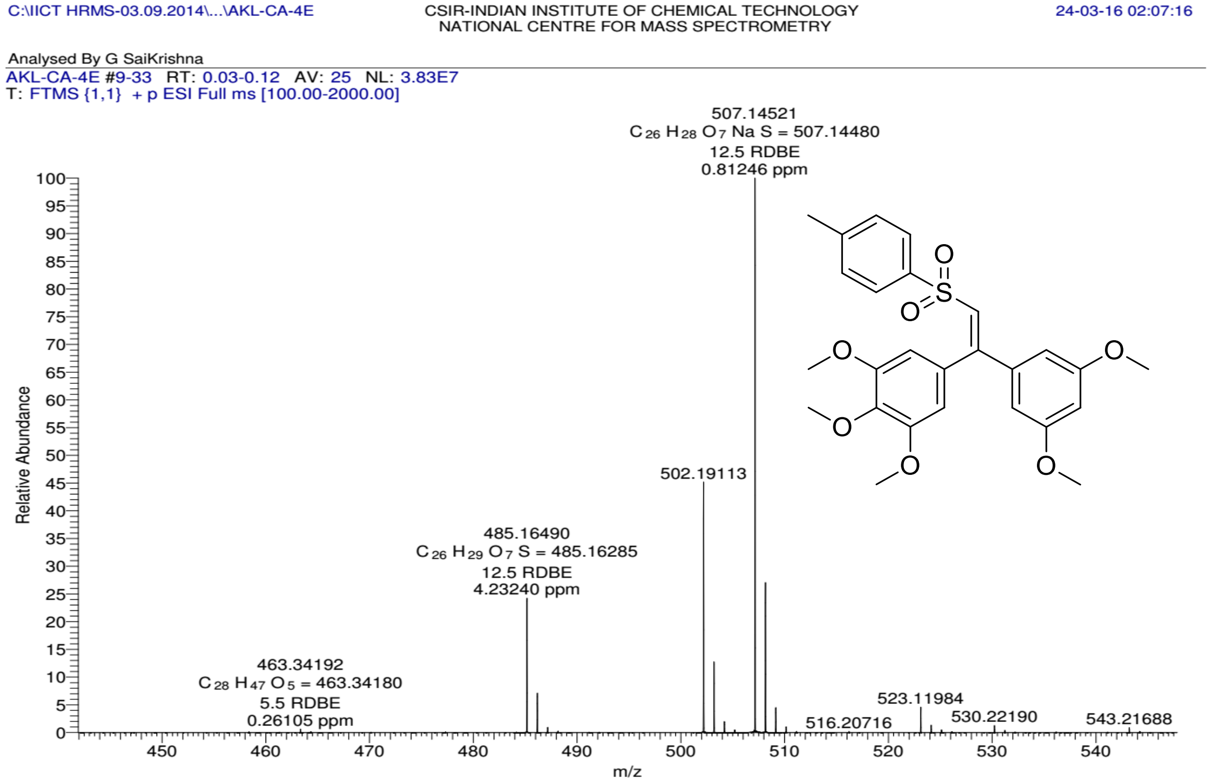

7. 4F, $C_{18}H_{18}O_5S$, 4-methylphenyl (3,4,5-trimethoxyphenyl)ethynyl sulfone

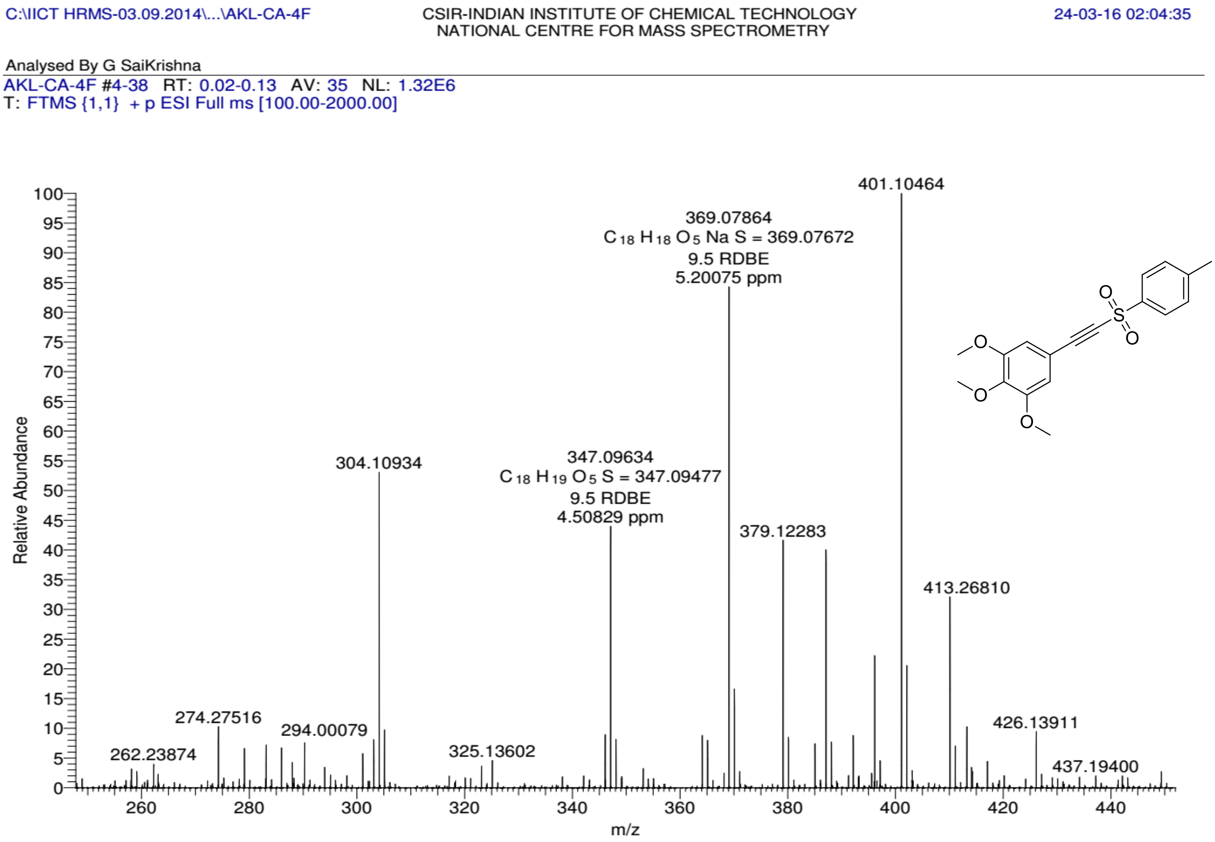

8. 4G, $C_{24}H_{23}FO_5S$, (Z)-5-(1-(4-fluorophenyl)-2-tosylvinyl)-1,2,3-trimethoxybenzene

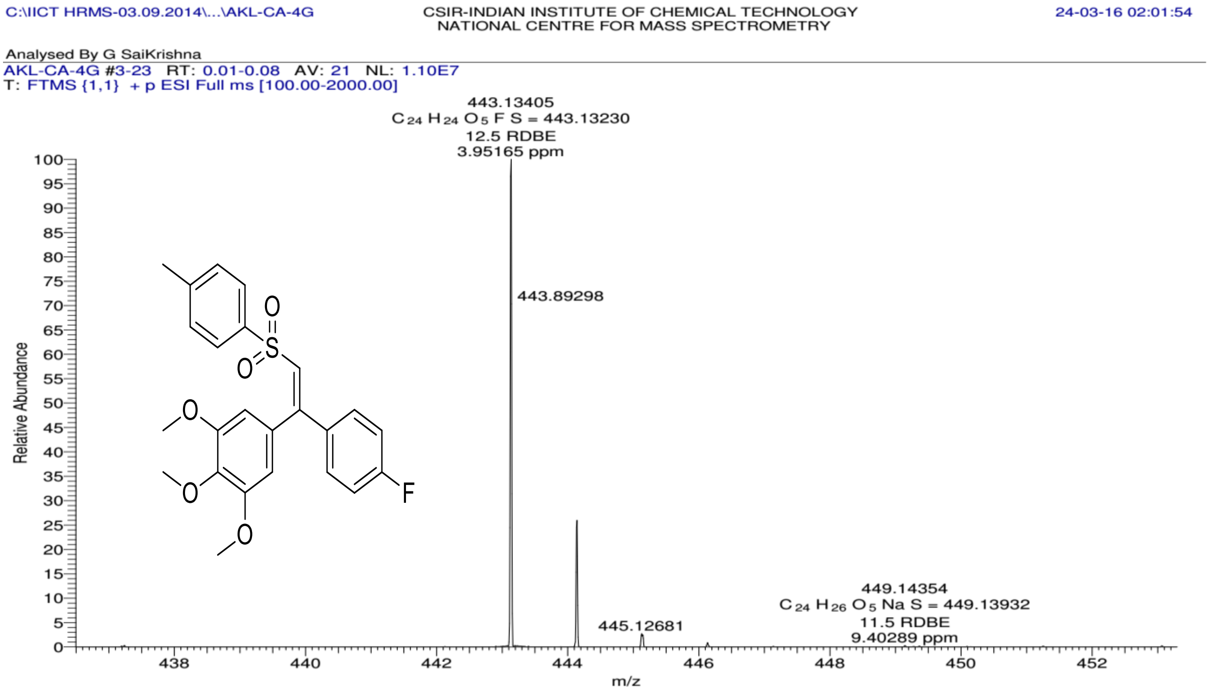

9. 4H, $C_{25}H_{23}F_3O_5S$, (Z)-1,2,3-trimethoxy-5-(2-tosyl-1-(4-(trifluoromethyl)phenyl)vinyl)benzene

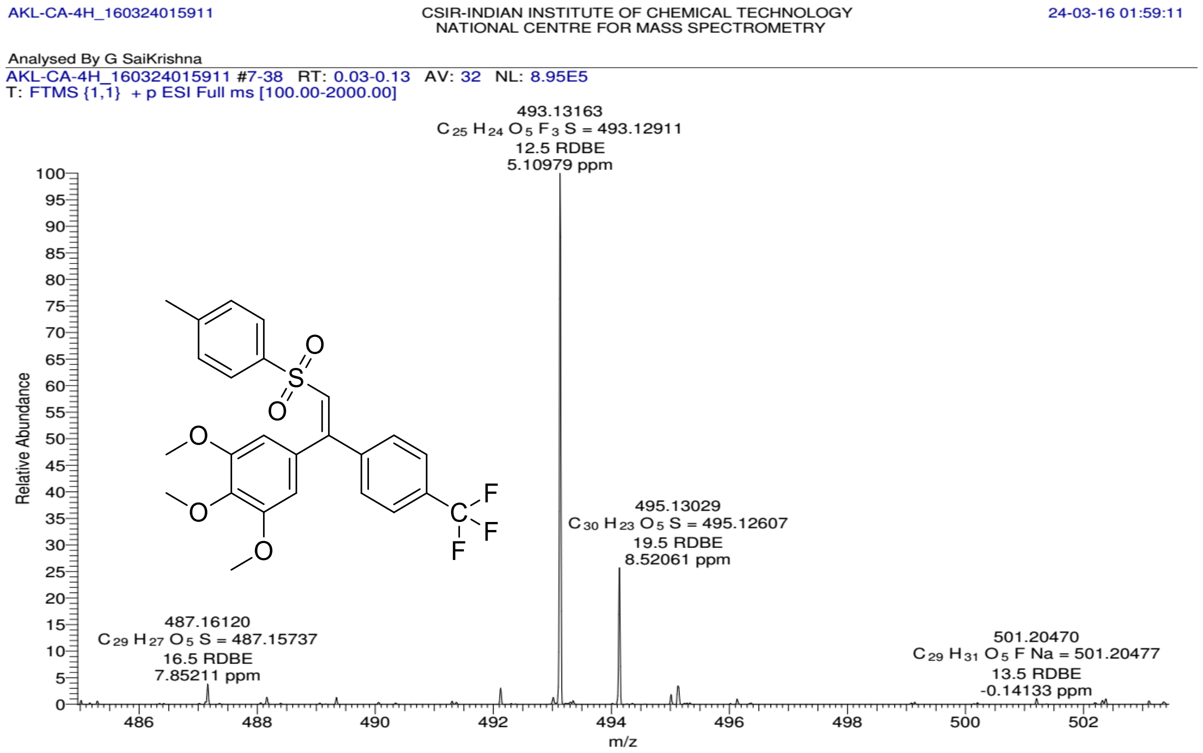

10. 4I, $C_{24}H_{22}F_2O_5S$, (E)-5-(1-(2,4-difluorophenyl)-2-tosylvinyl)-1,2,3-trimethoxybenzene

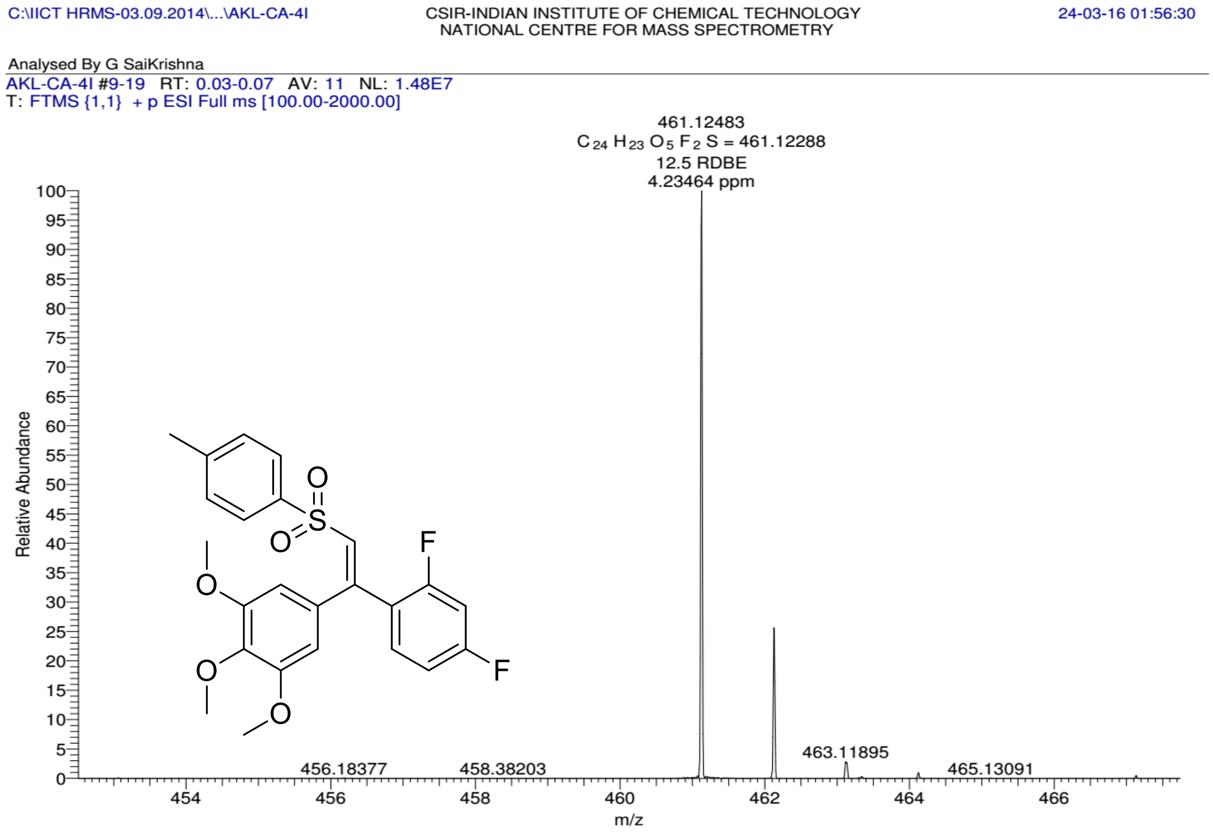

11. 4J, $C_{24}H_{22}F_2O_5S$, (E)-5-(1-(3,4-difluorophenyl)-2-tosylvinyl)-1,2,3-trimethoxybenzene

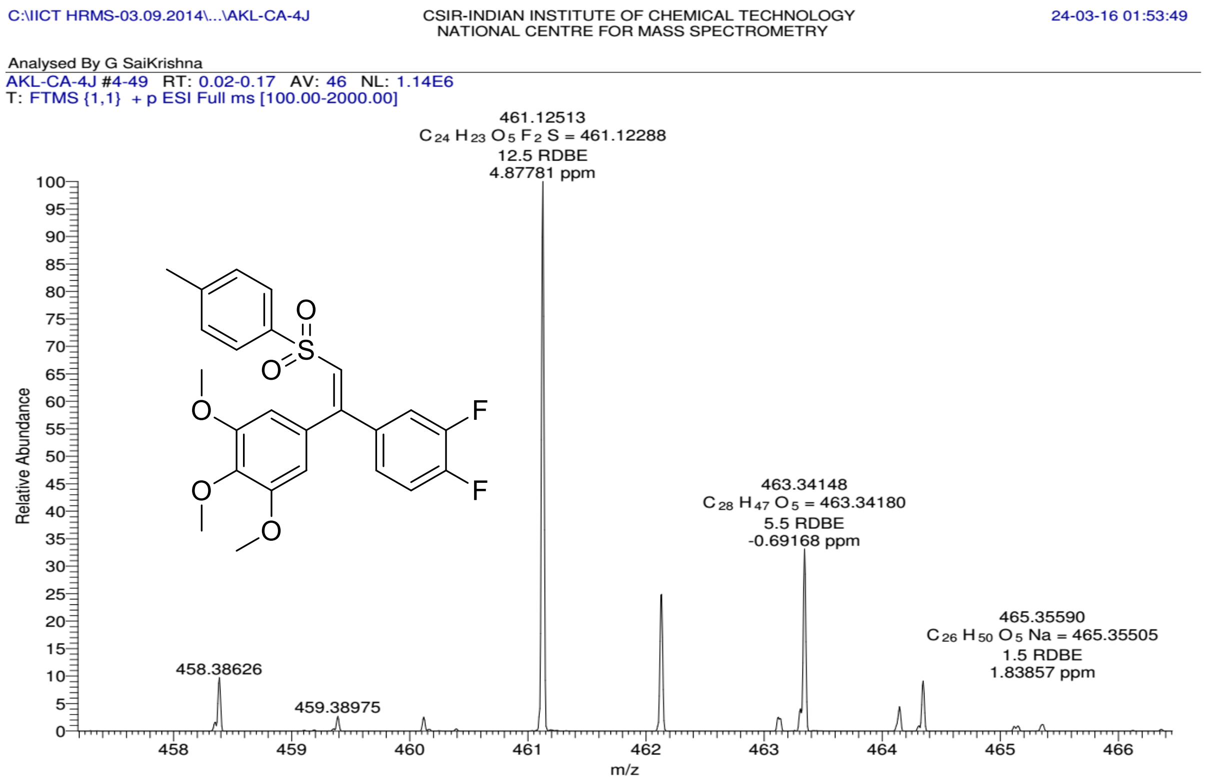

12. 4K, $C_{26}H_{27}FO_6S$, (E)-5-(1-(5-ethoxy-2-fluorophenyl)-2-tosylvinyl)-1,2,3-trimethoxybenzene

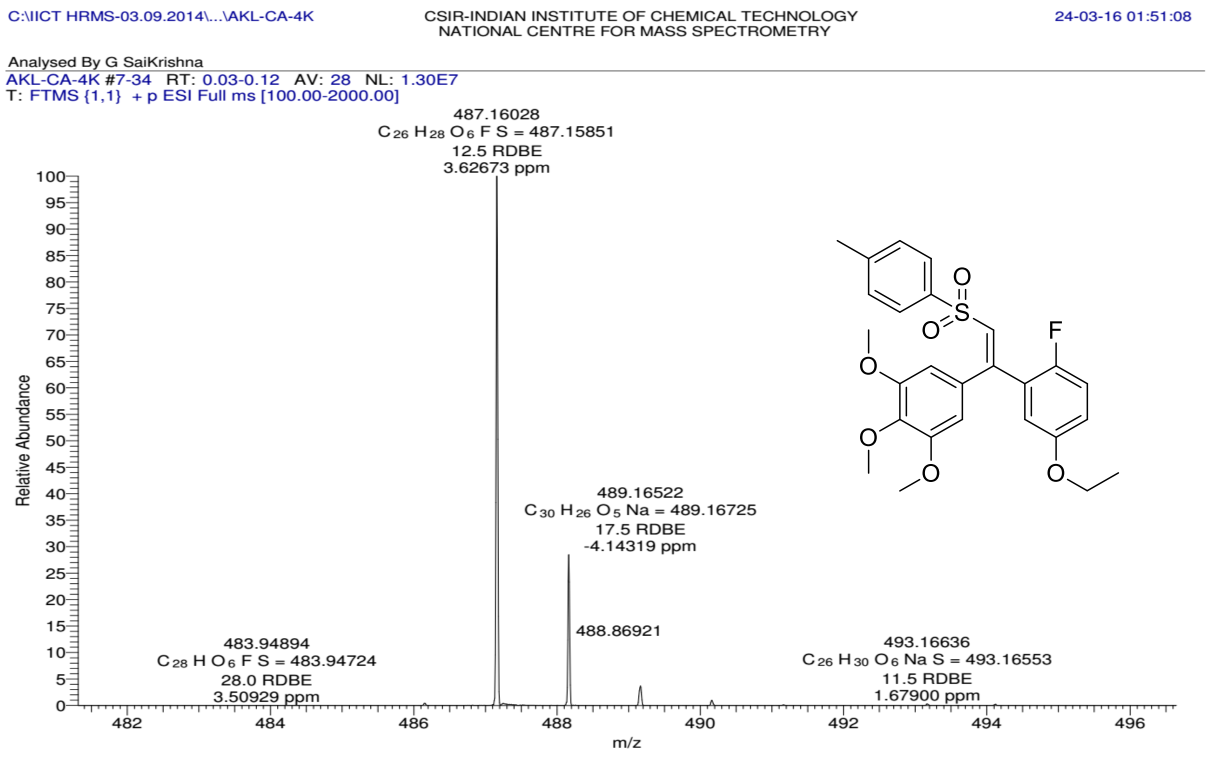

13. 4L, $C_{24}H_{22}ClFO_5S$, (E)-5-(1-(3-chloro-4-fluorophenyl)-2-tosylvinyl)-1,2,3-trimethoxybenzene

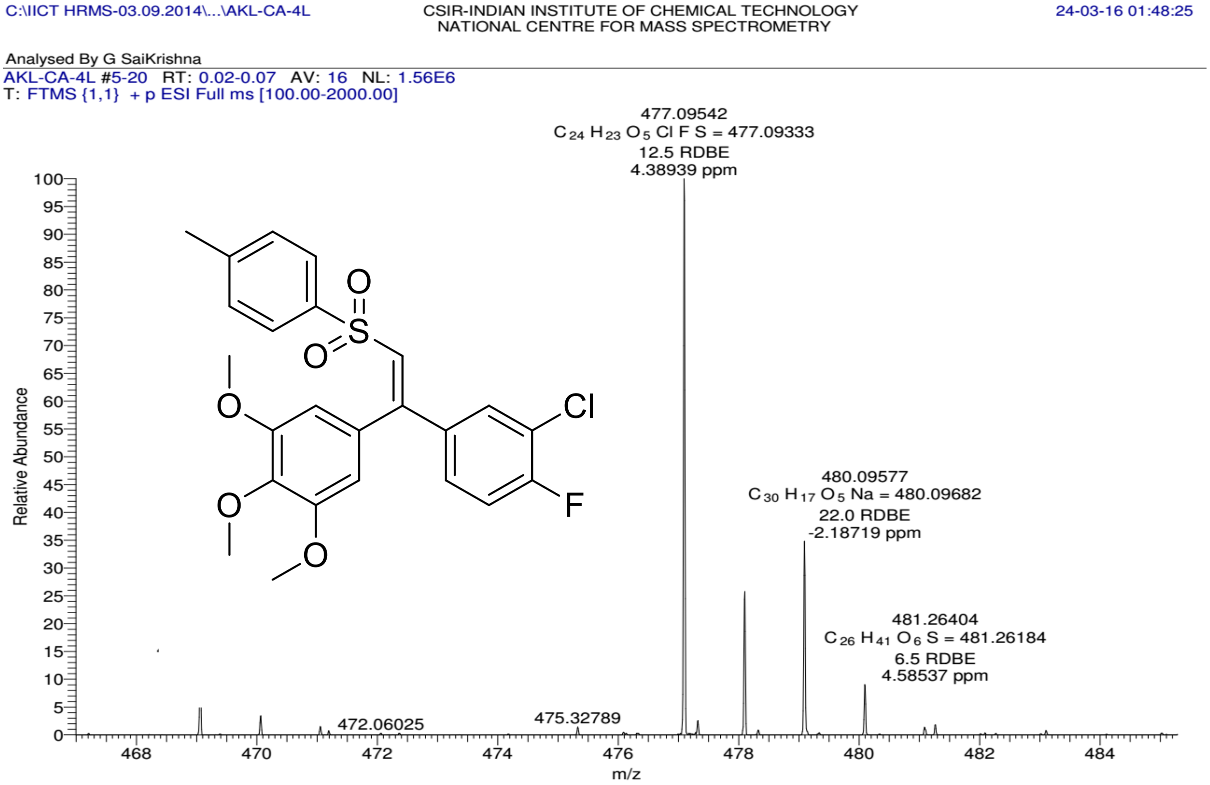

14. 4M, $C_{24}H_{23}ClO_5S$, (Z)-5-(1-(4-chlorophenyl)-2-tosylvinyl)-1,2,3-trimethoxybenzene

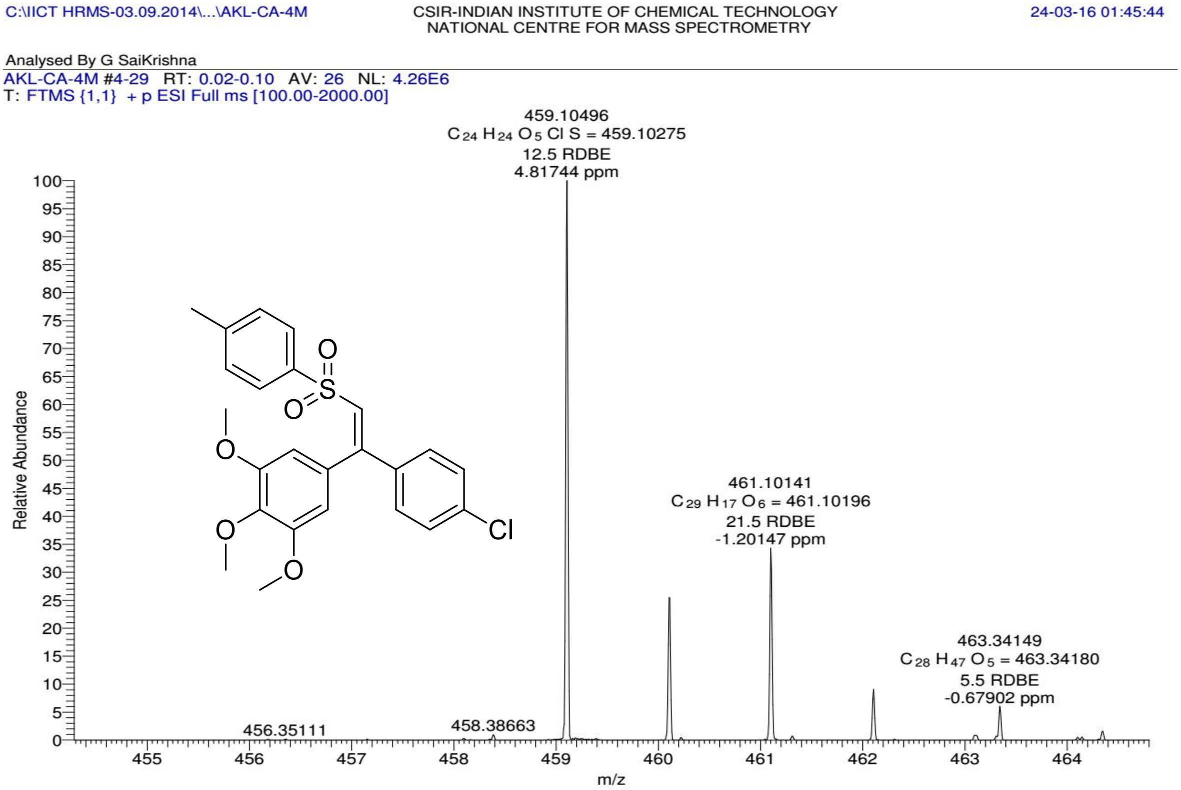

15. 4N, $C_{24}H_{22}Cl_2O_5S$, (E)-5-(1-(3,4-dichlorophenyl)-2-tosylvinyl)-1,2,3-trimethoxybenzene

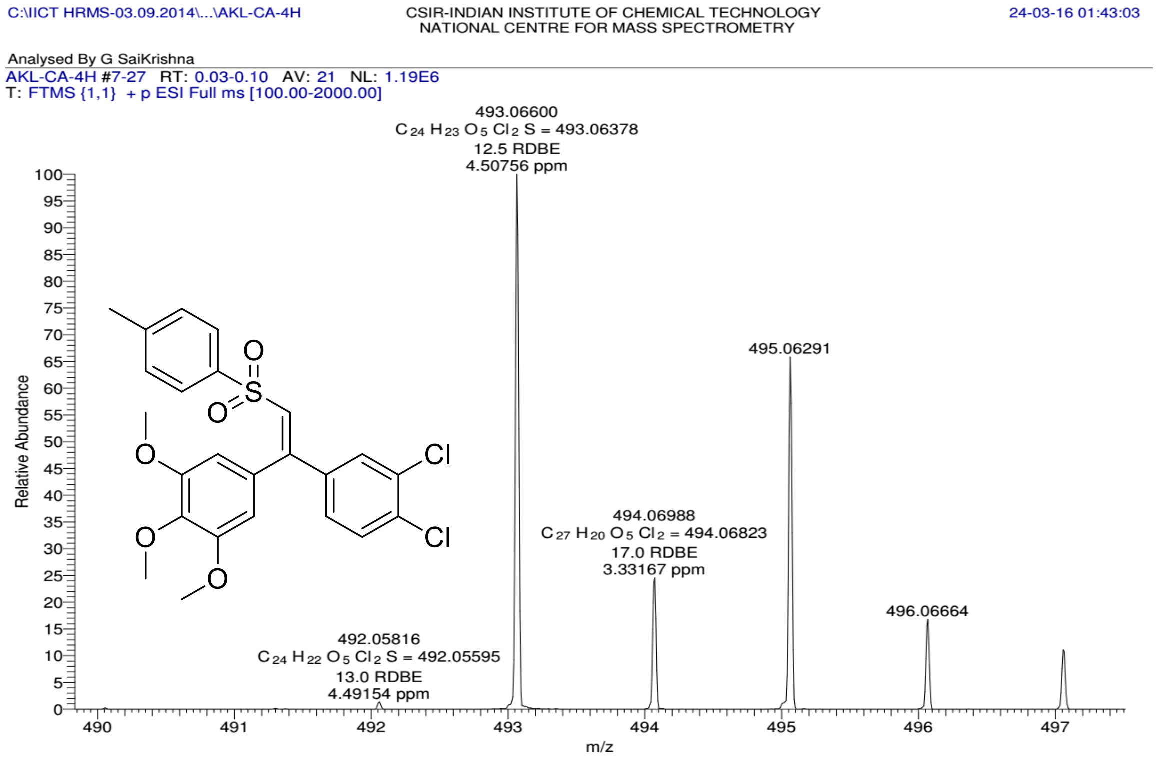

16. 4O, $C_{25}H_{23}NO_5S$, (Z)-4-(2-tosyl-1-(3,4,trimethoxyphenyl)vinyl)benzonitrile

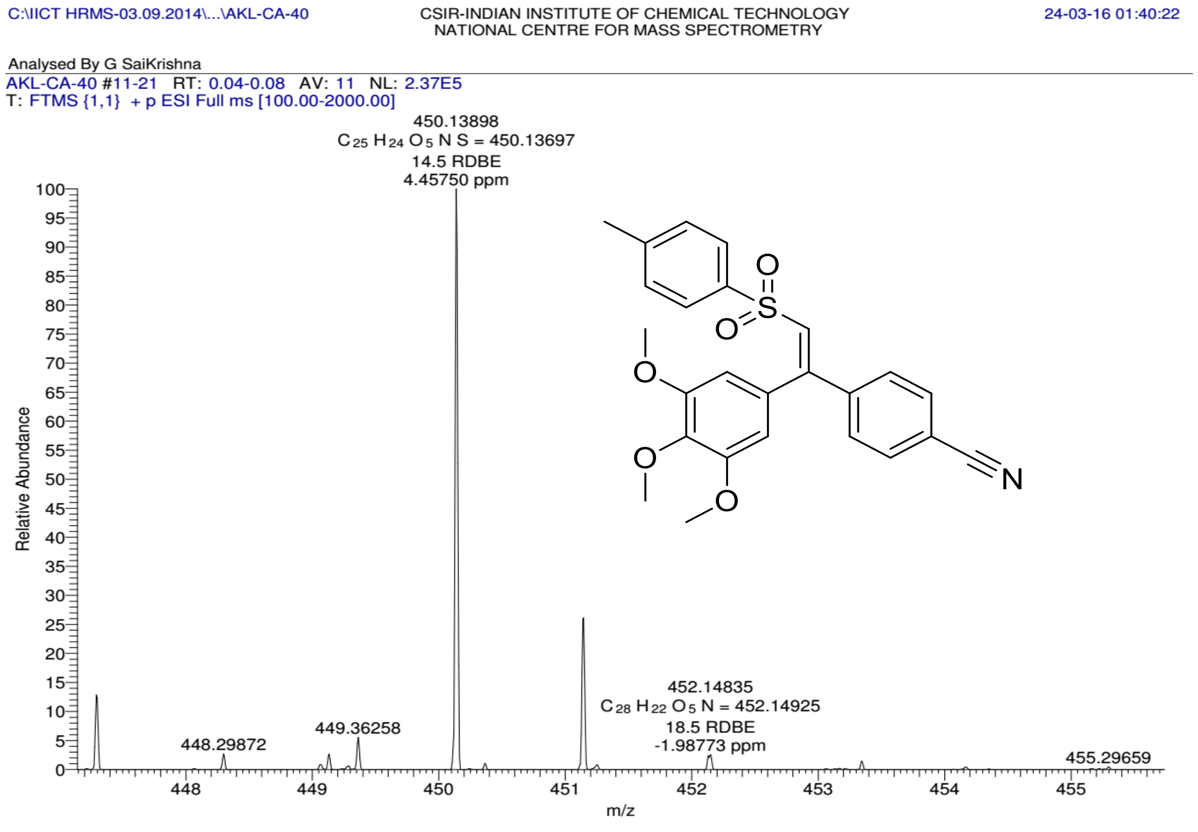

17. 4Q, $C_{25}H_{22}F_4O_5S$, (E)-5-(1-(2-fluoro-3-(trifluoromethyl)phenyl)-2-tosylvinyl)-1,2,3-trimethoxybenzene

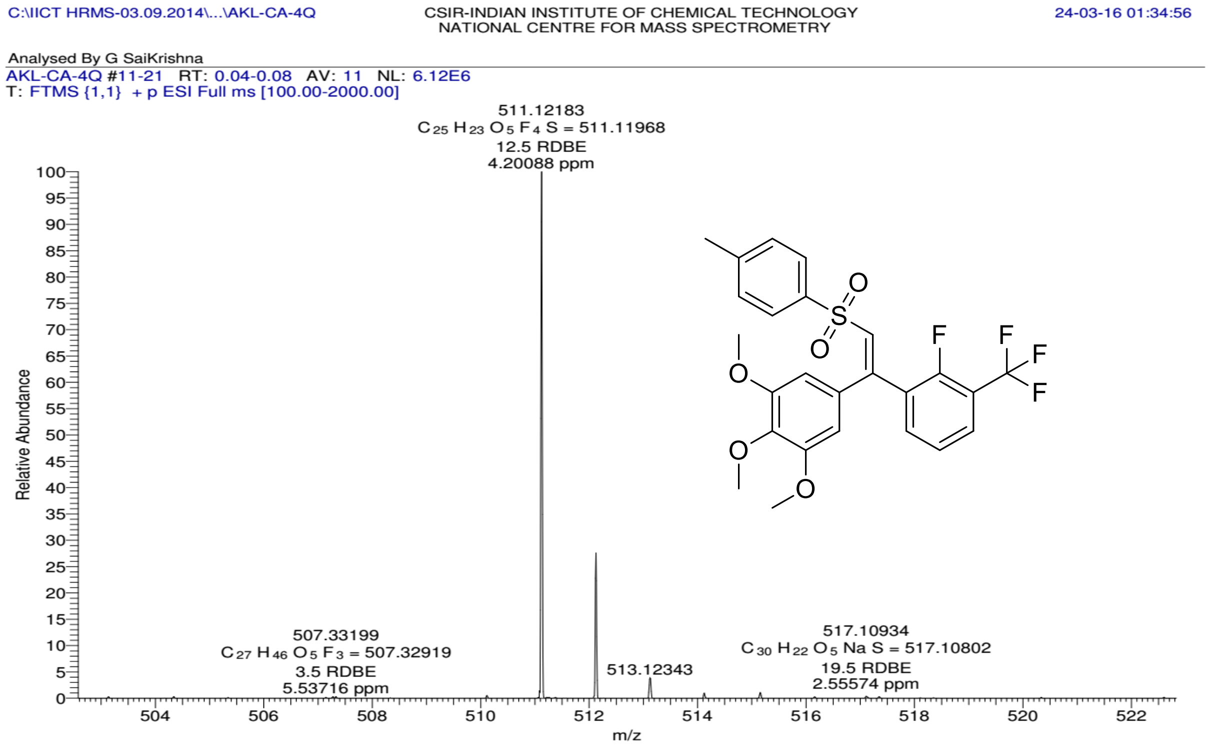

18. 4R, $C_{25}H_{25}FO_5S$, (E)-5-(1-(2-fluoro-5-methylphenyl)-2-tosylvinyl)-1,2,3-

Trimethoxybenzene

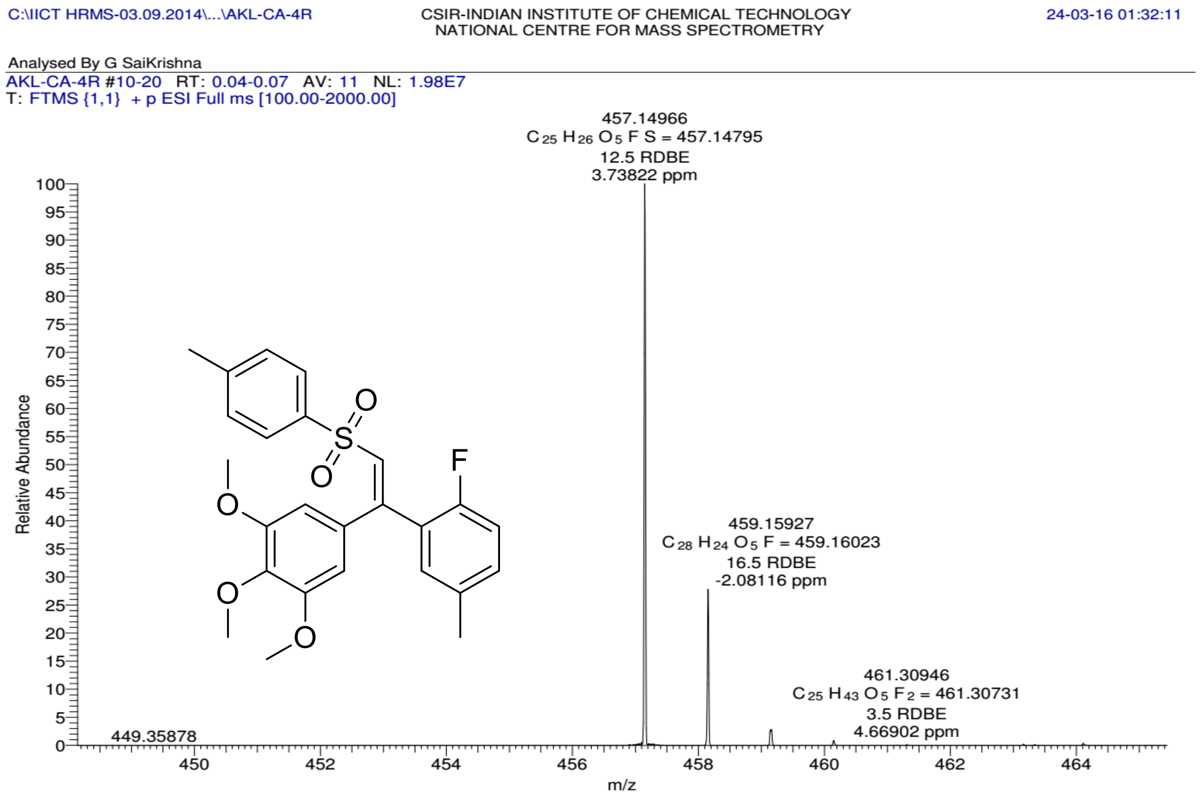

19. 4T, $C_{25}H_{25}FO_5S$, (E)-5-(1-(3-fluoro-4-methylphenyl)-2-tosylvinyl)-1,2,3-trimethoxybenzene

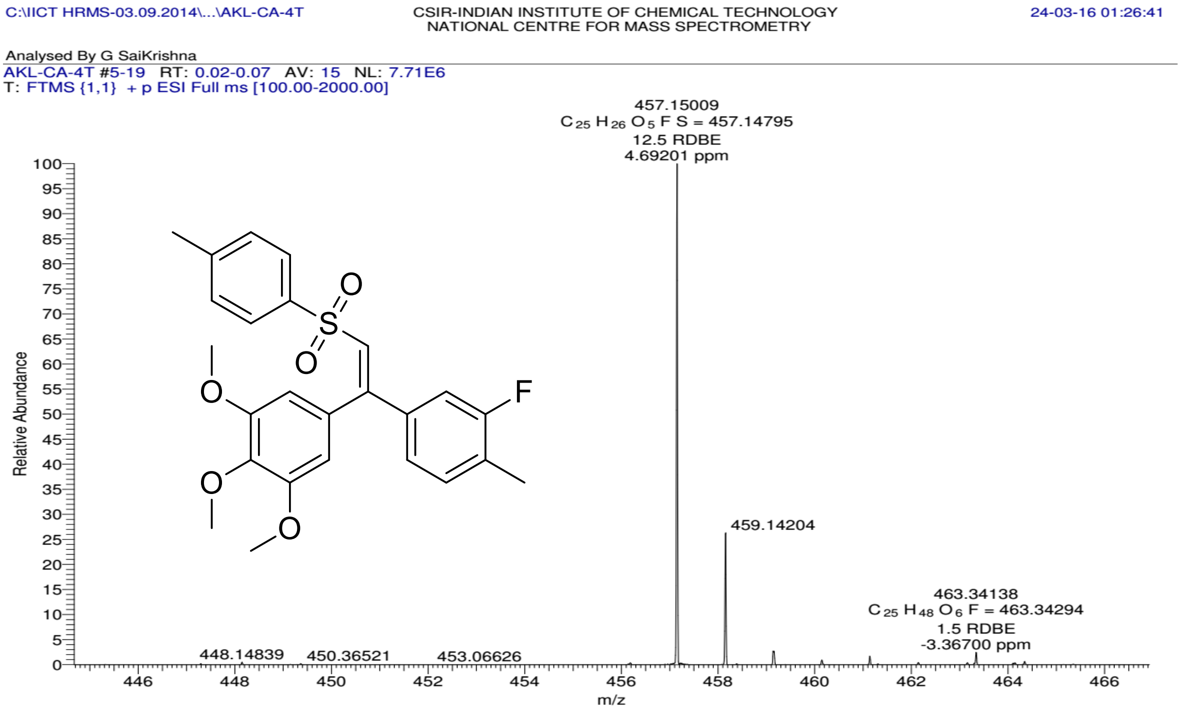

20. 4U, $C_{30}H_{28}O_5S$, (Z)-4-(2-tosyl-1-(3,4,5-trimethoxyphenyl)vinyl)-1,1'-biphenyl

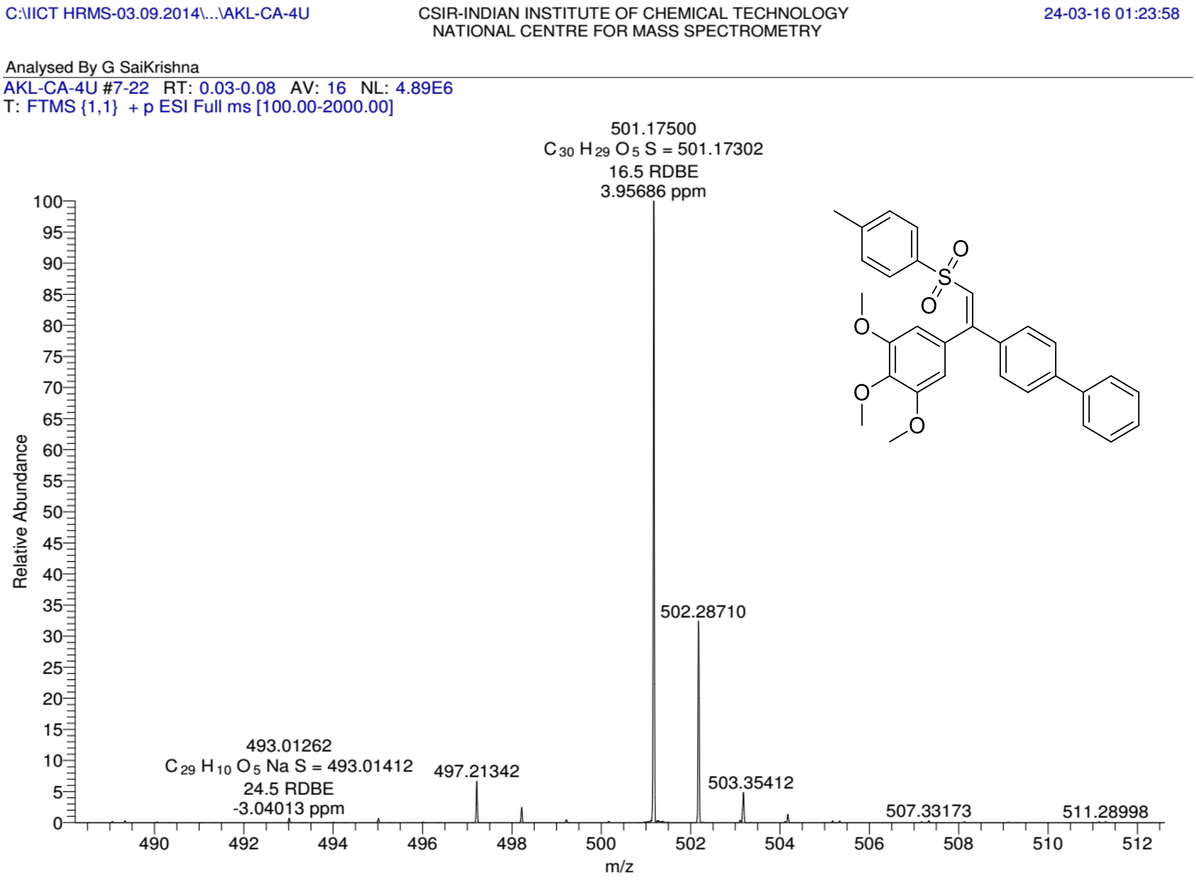

21. 4V, $C_{18}H_{18}O_5S$, 4-methylphenyl (3,4,5-trimethoxyphenyl)ethynyl sulfone

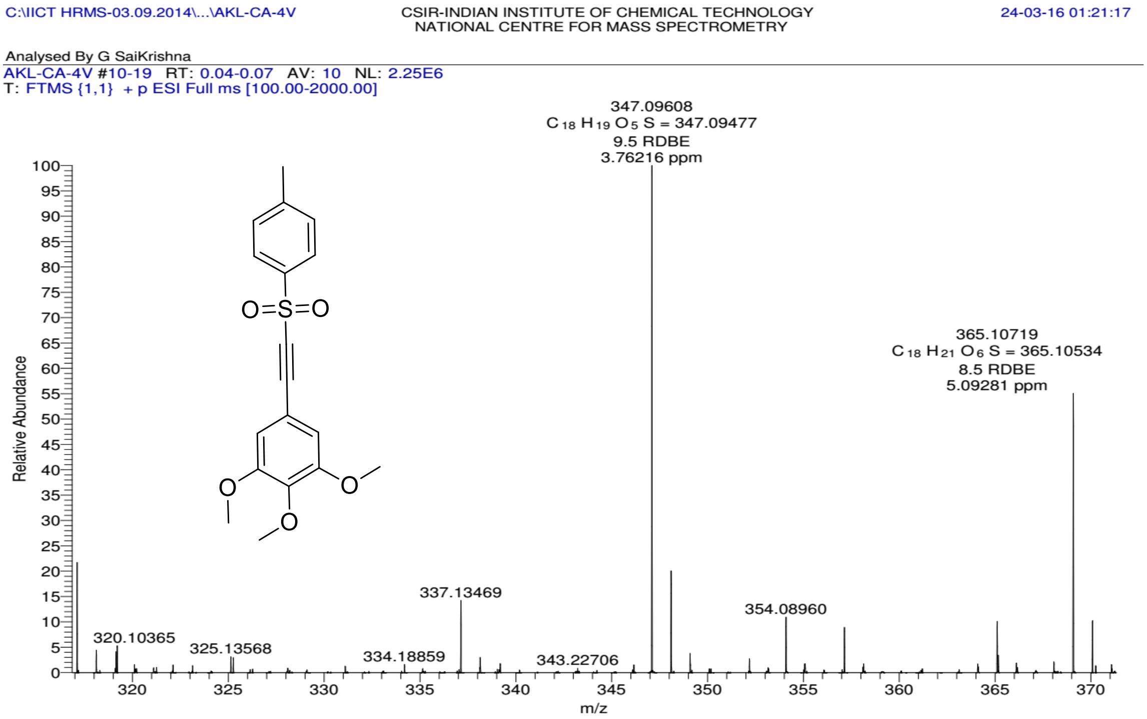

IR SPECTRA OF SYNTHESIZED COMPOUNDS

1. $C_{18}H_{19}IO_5S$, (E)-5-(1-iodo-2-tosylvinyl)-1,2,3-trimethoxybenzene

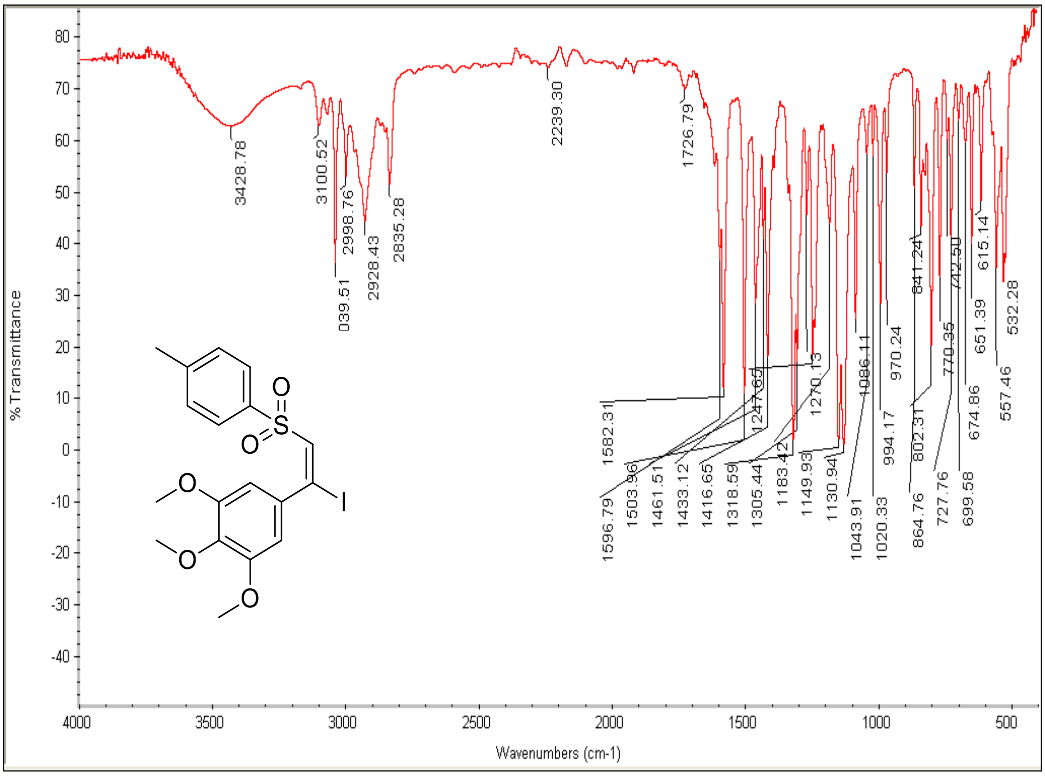

2. 4A, $C_{24}H_{24}O_5S$, (Z)-1,2,3-trimethoxy-5-(1-phenyl-2-tosylvinyl)benzene

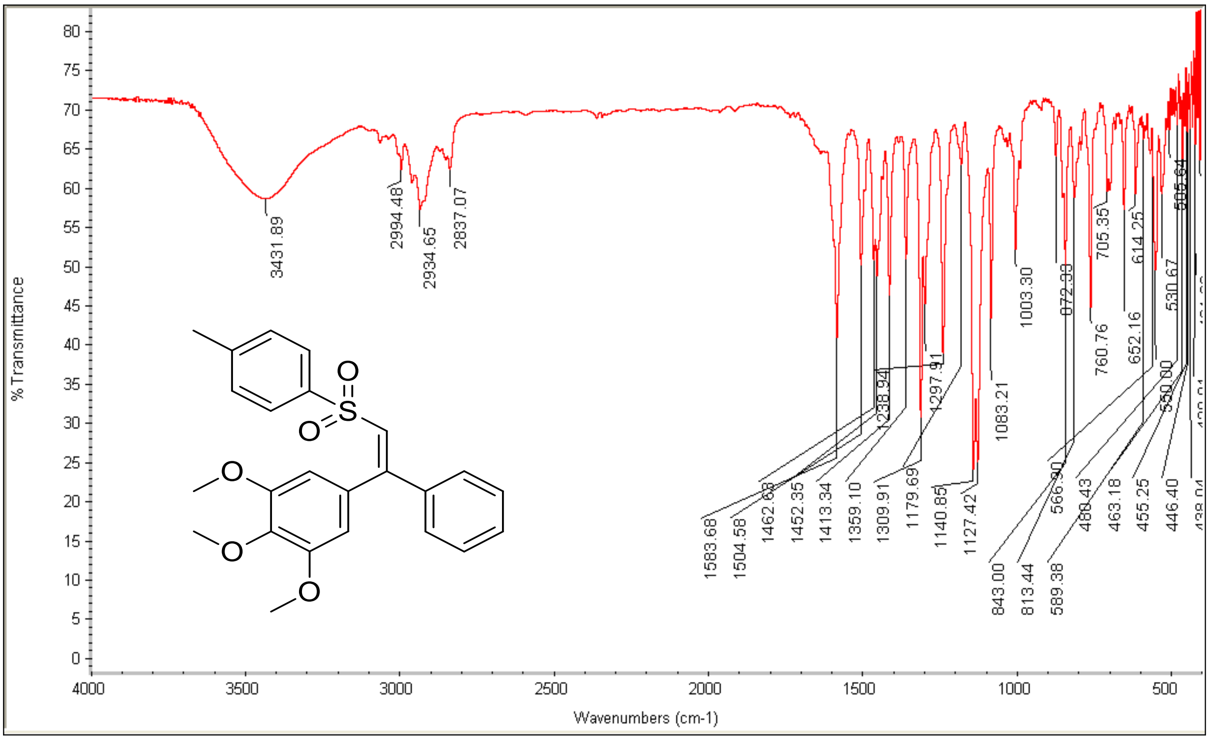

3. 4B, (Z)-1,2,3-trimethoxy-5-(1-(4-methoxyphenyl)-2-tosylvinyl)benzene

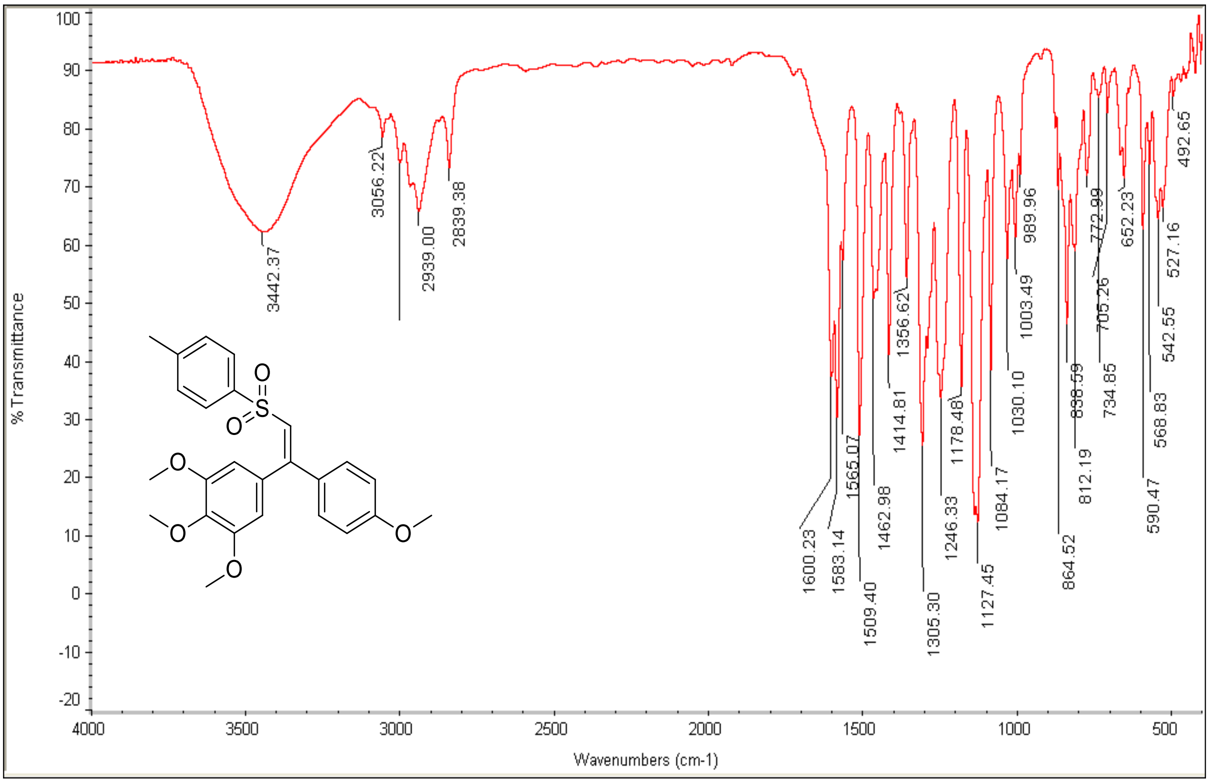

4. 4C, $C_{26}H_{28}O_7S$, (E)-5-(1-(2,4-dimethoxyphenyl)-2-tosylvinyl)-1,2,3-trimethoxybenzene

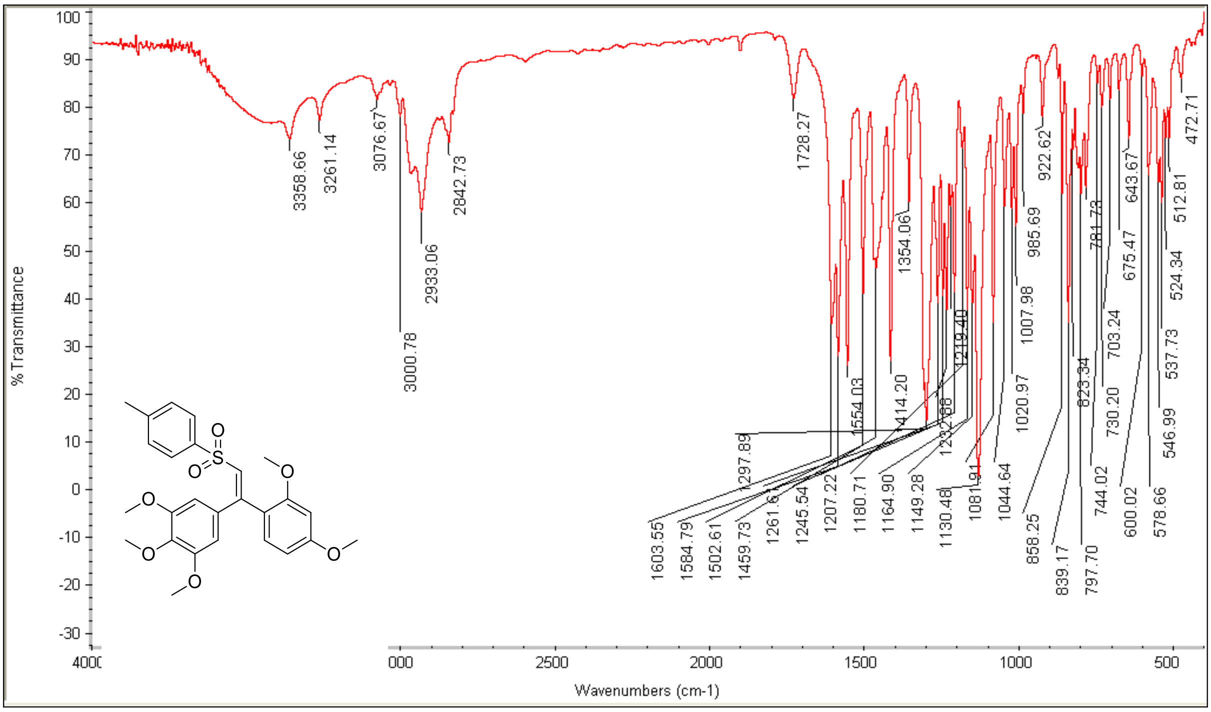

5. 4D, $C_{26}H_{28}O_7S$, (Z)-5-(1-(3,4-dimethoxyphenyl)-2-tosylvinyl)-1,2,3-trimethoxybenzene

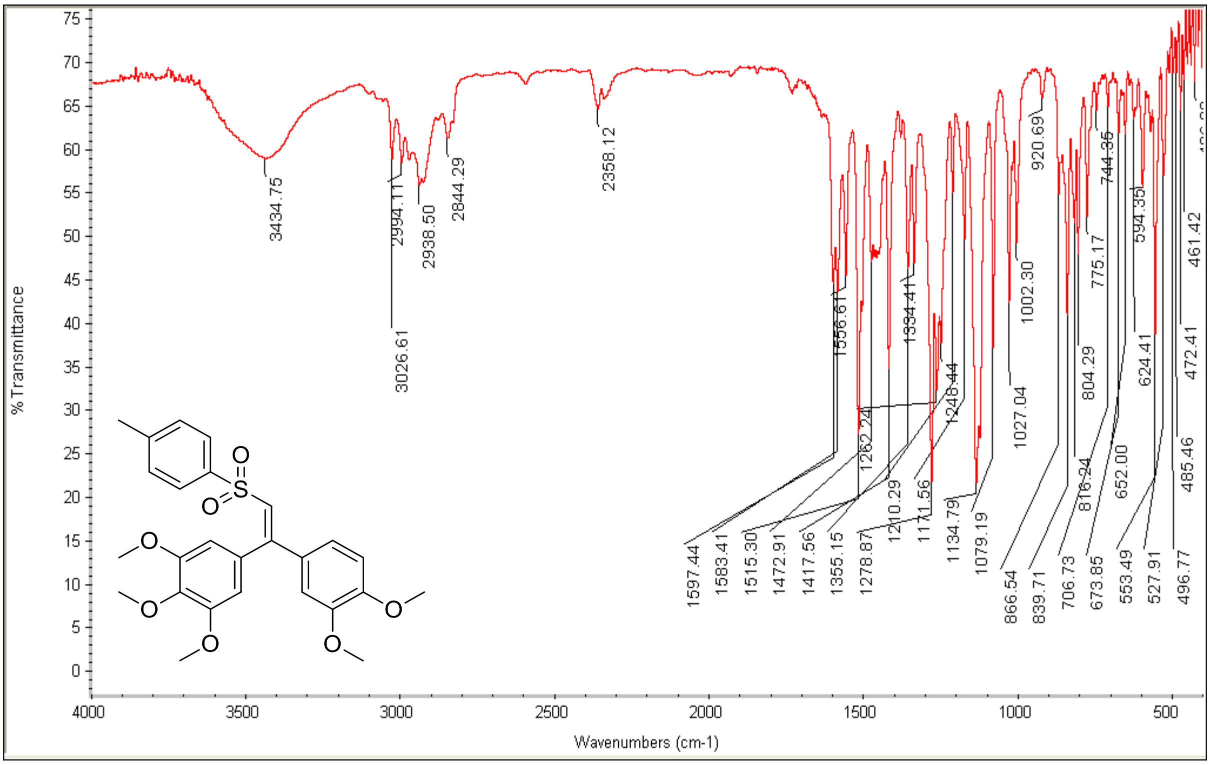

6. 4E, $C_{26}H_{28}O_7S$, (Z)-5-(1-(3,5-dimethoxyphenyl)-2-tosylvinyl)-1,2,3-trimethoxybenzene

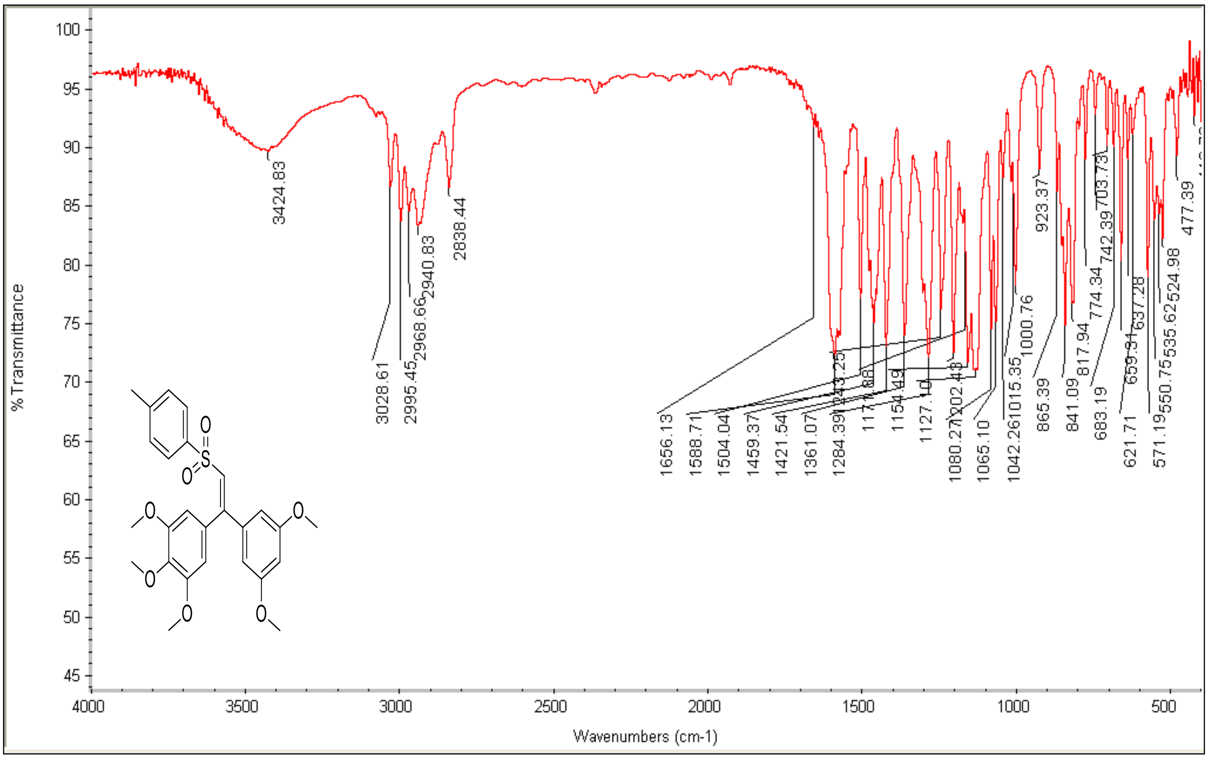

7. **4F, $C_{18}H_{18}O_5S$, 4-methylphenyl (3,4,5-trimethoxyphenyl)ethynyl sulfone**

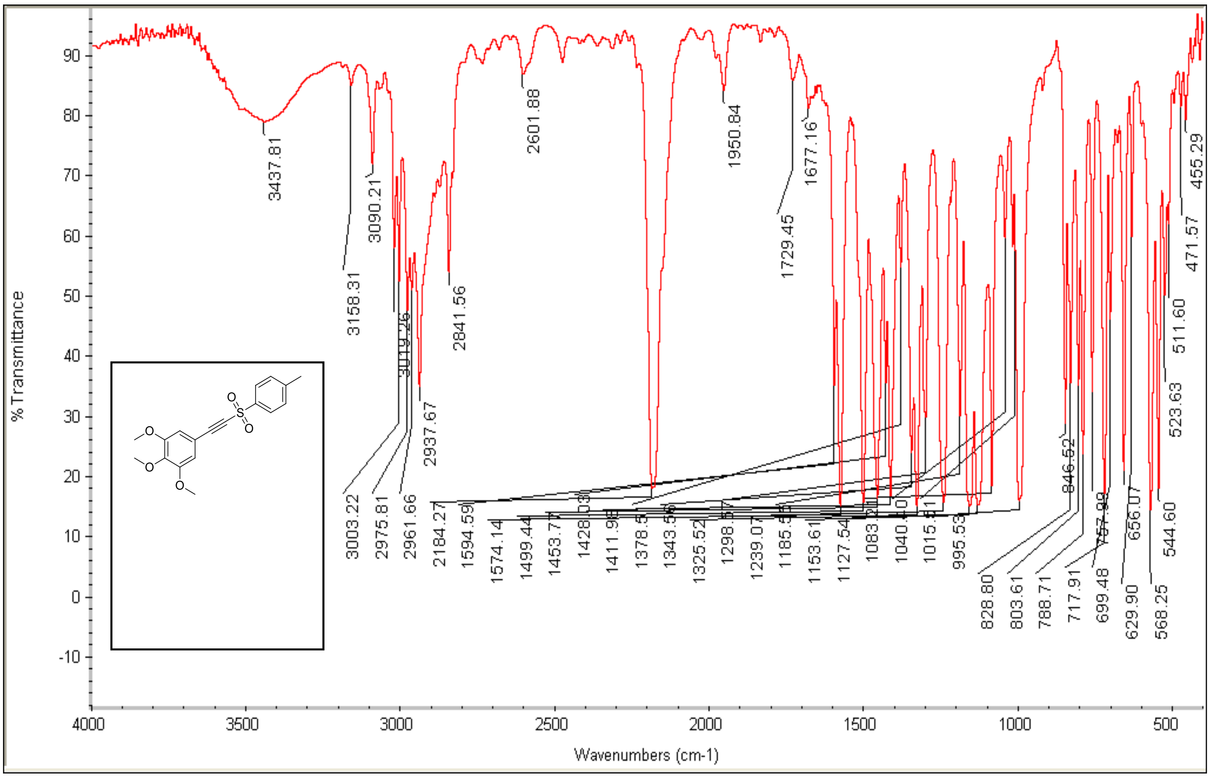

8. **4G, $C_{24}H_{23}FO_5S$, (Z)-5-(1-(4-fluorophenyl)-2-tosylvinyl)-1,2,3-trimethoxybenzene**

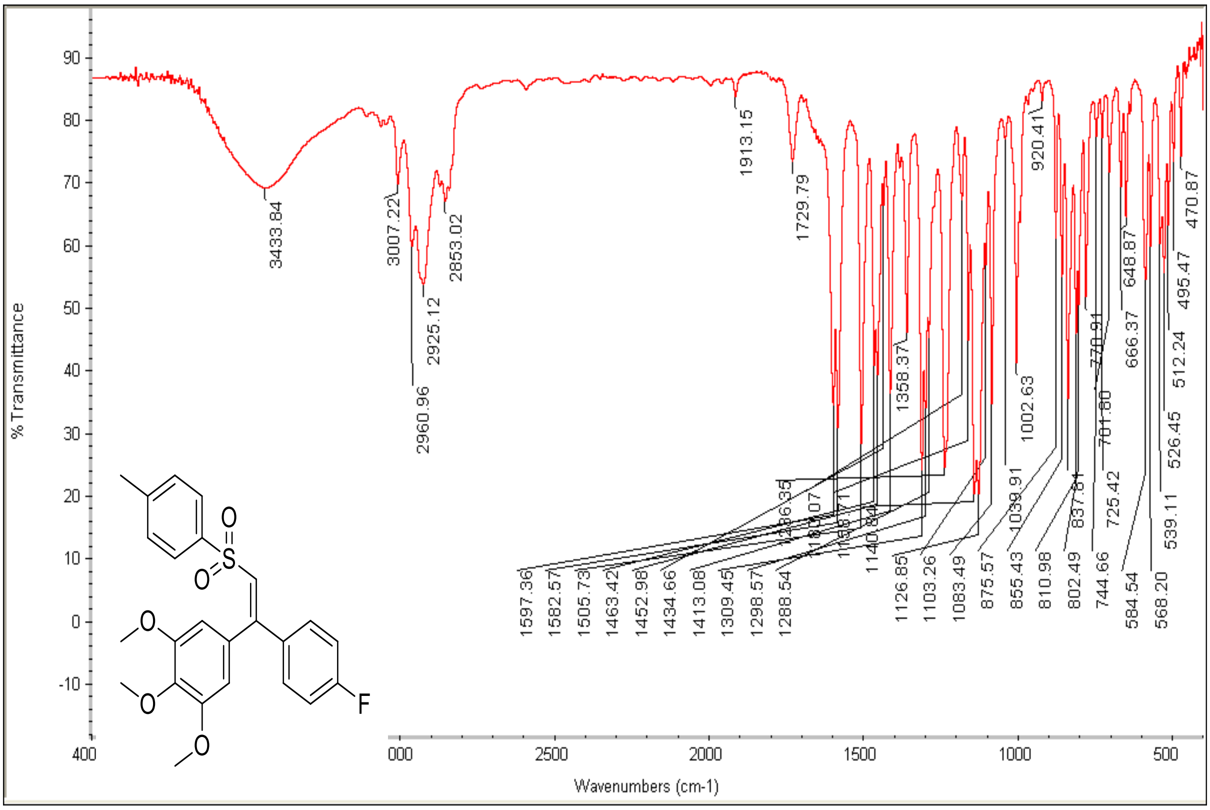

9. 4H, $C_{25}H_{23}F_3O_5S$, (Z)-1,2,3-trimethoxy-5-(2-tosyl-1-(4-(trifluoromethyl)phenyl)vinyl)benzene

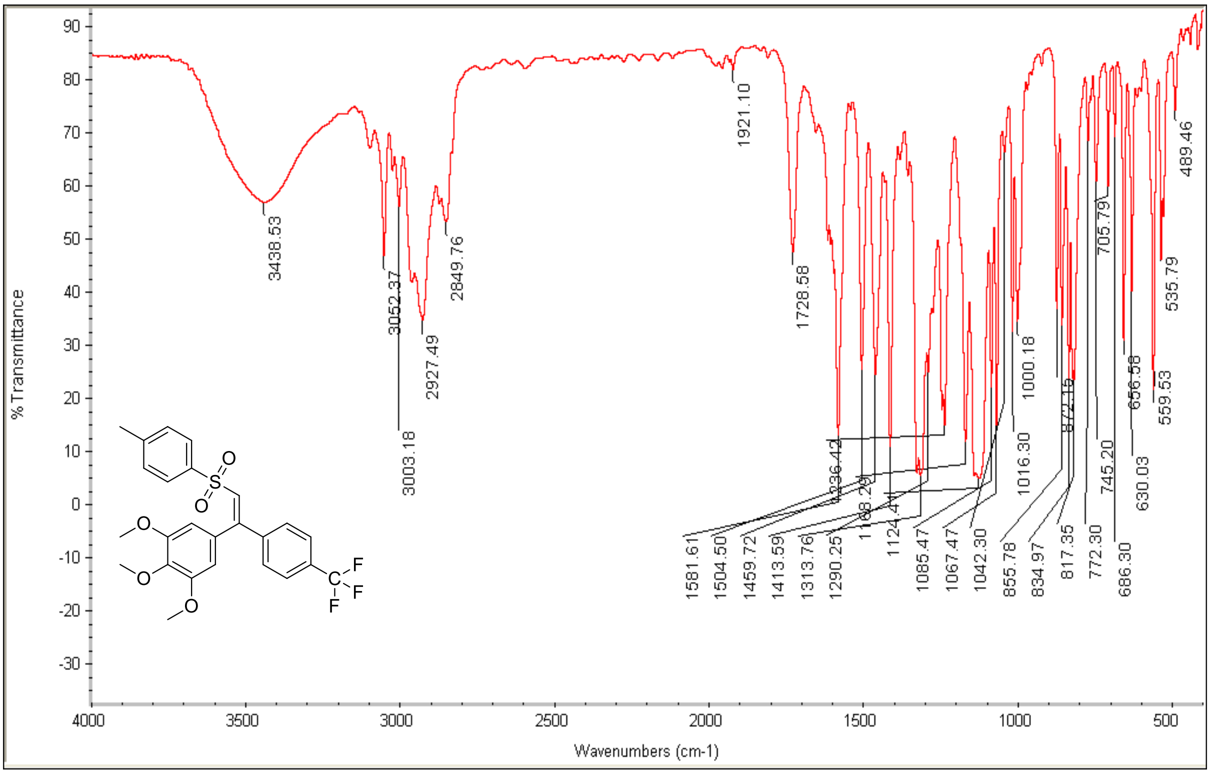

10. 4I, $C_{24}H_{22}F_2O_5S$, (E)-5-(1-(2,4-difluorophenyl)-2-tosylvinyl)-1,2,3-trimethoxybenzene

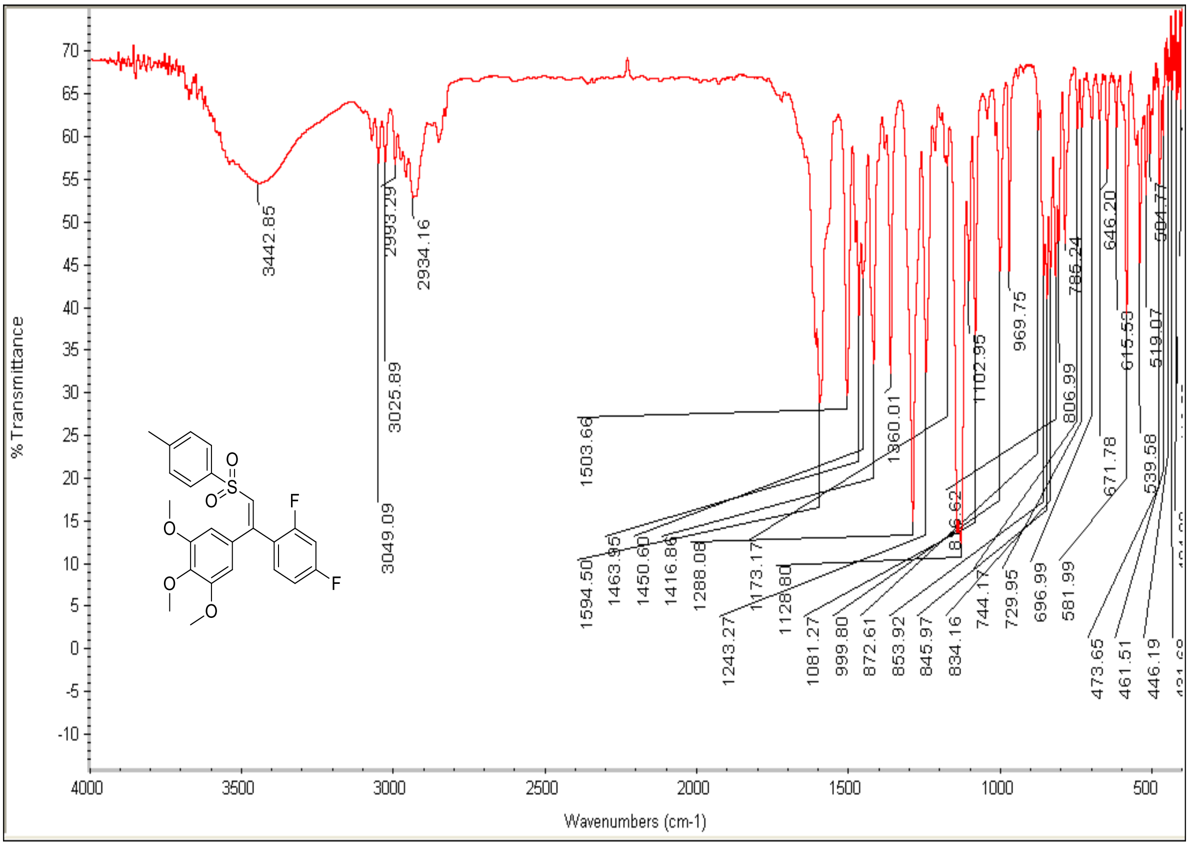

11. 4J, $C_{24}H_{22}F_2O_5S$, (E)-5-(1-(3,4-difluorophenyl)-2-tosylvinyl)-1,2,3-trimethoxybenzene

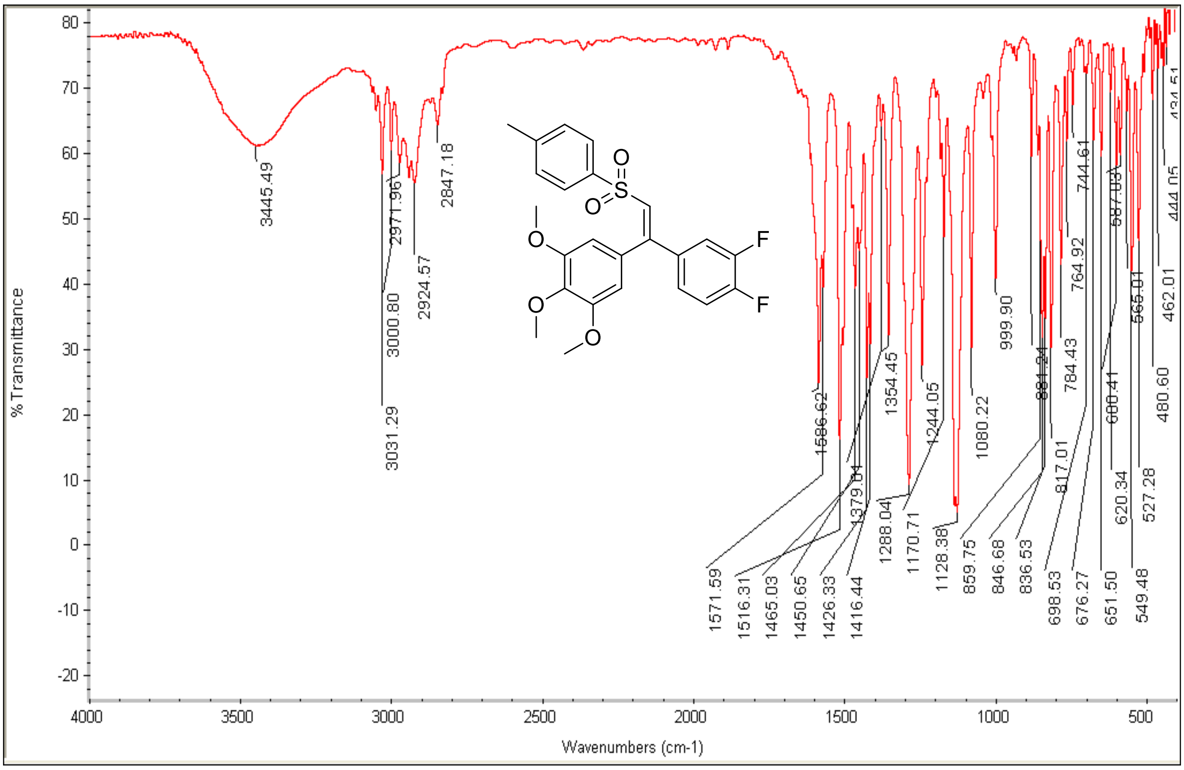

12. 4K, $C_{26}H_{27}FO_6S$, (E)-5-(1-(5-ethoxy-2-fluorophenyl)-2-tosylvinyl)-1,2,3-trimethoxybenzene

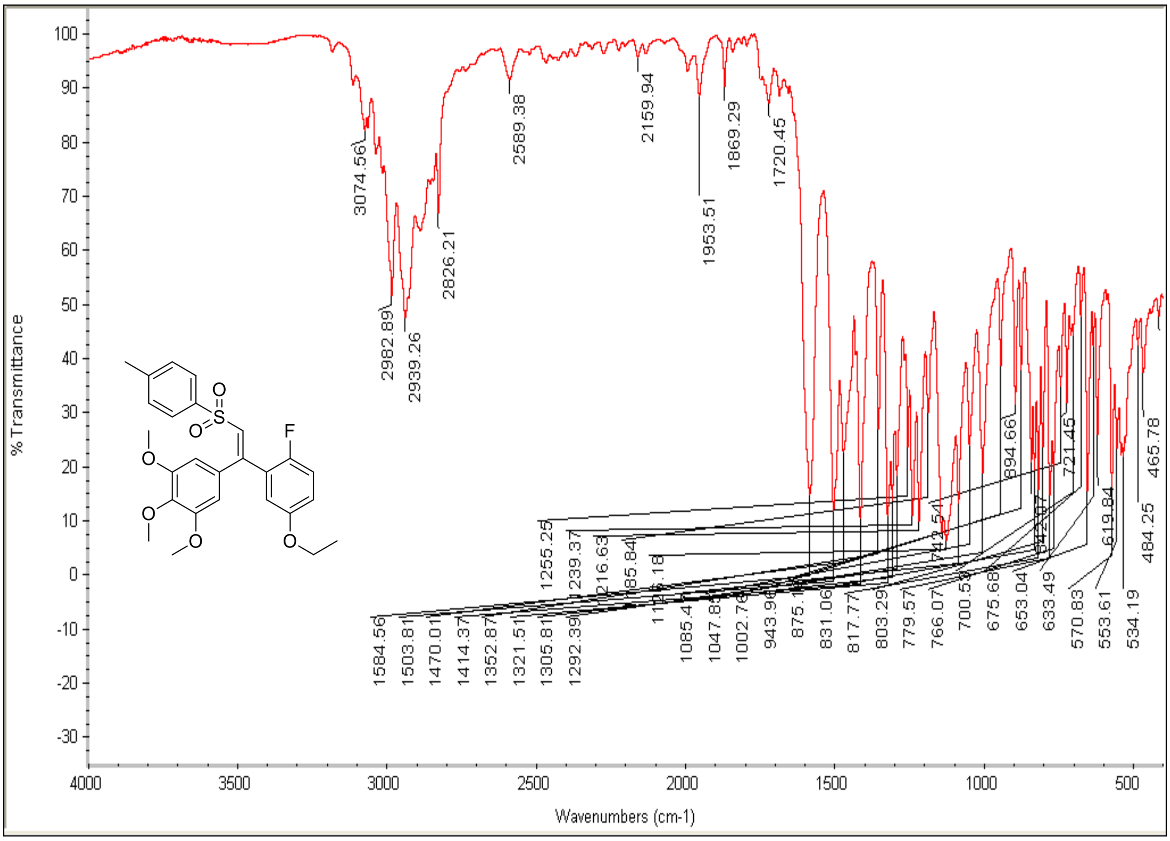

13. 4L, $C_{24}H_{22}ClFO_5S$, (E)-5-(1-(3-chloro-4-fluorophenyl)-2-tosylvinyl)-1,2,3-trimethoxybenzene

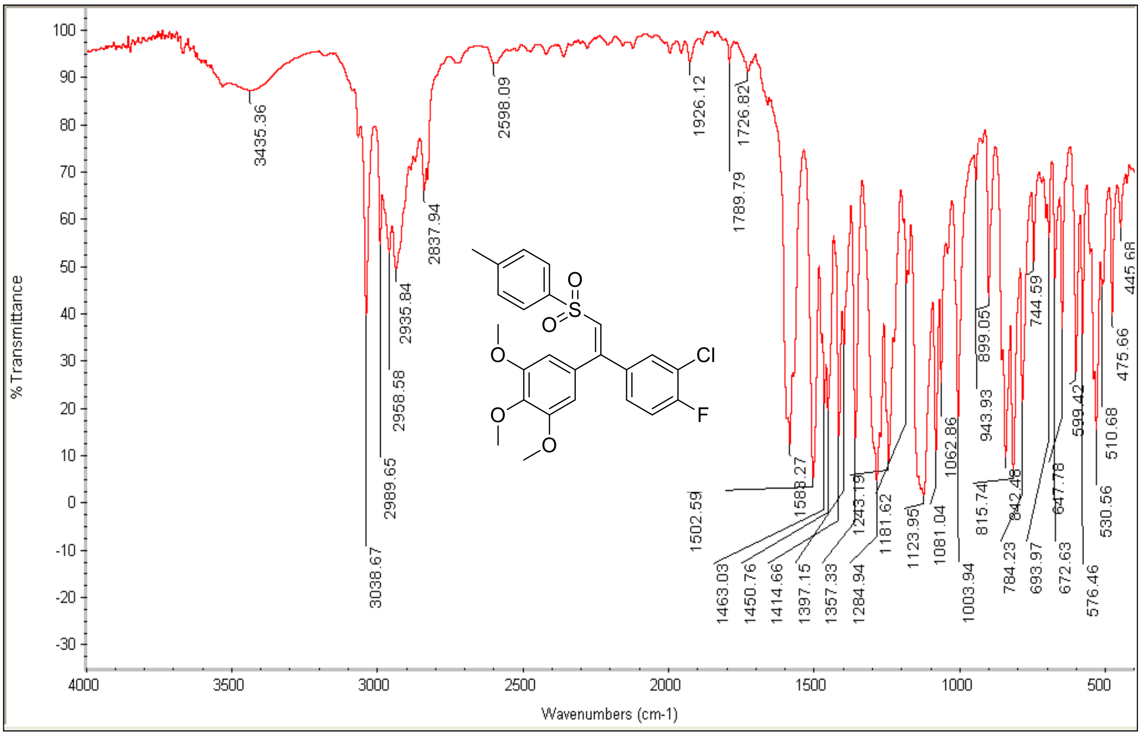

14. 4M, $C_{24}H_{23}ClO_5S$, (Z)-5-(1-(4-chlorophenyl)-2-tosylvinyl)-1,2,3-trimethoxybenzene

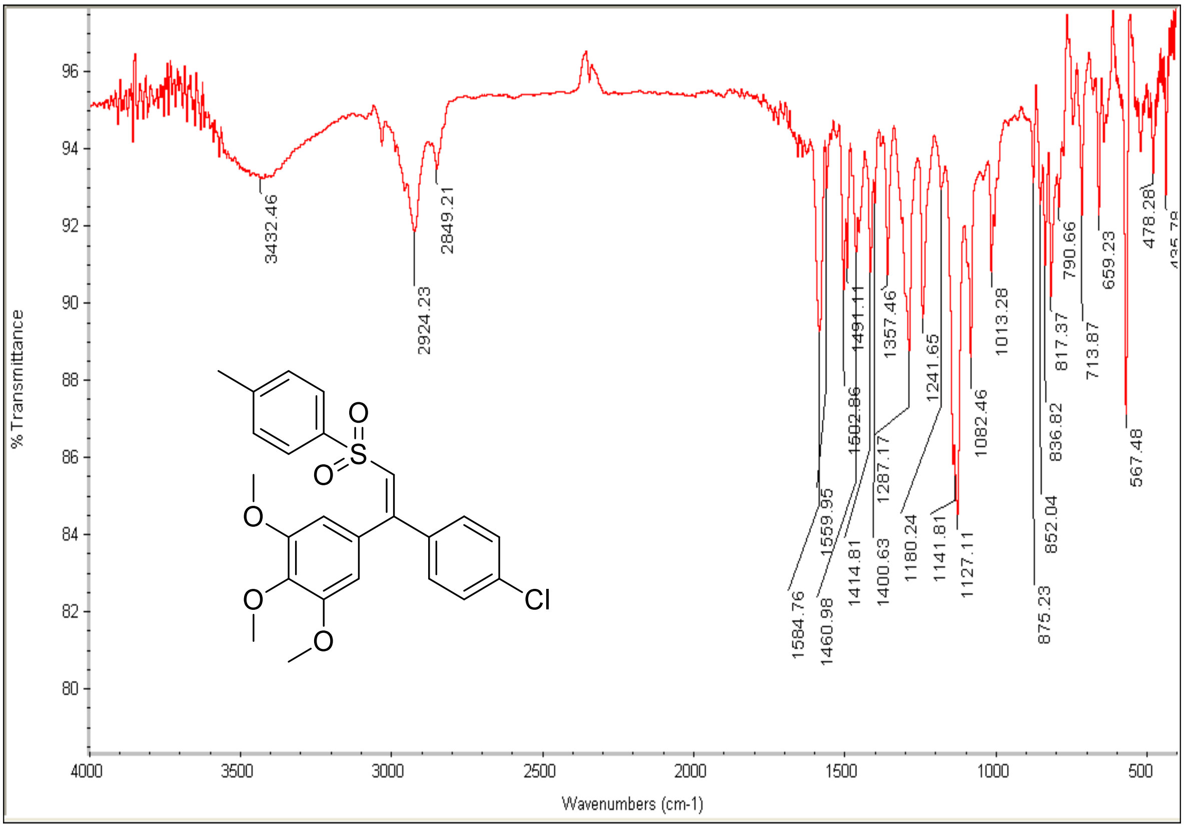

15. 4N, $C_{24}H_{22}Cl_2O_5S$, (E)-5-(1-(3,4-dichlorophenyl)-2-tosylvinyl)-1,2,3-trimethoxybenzene

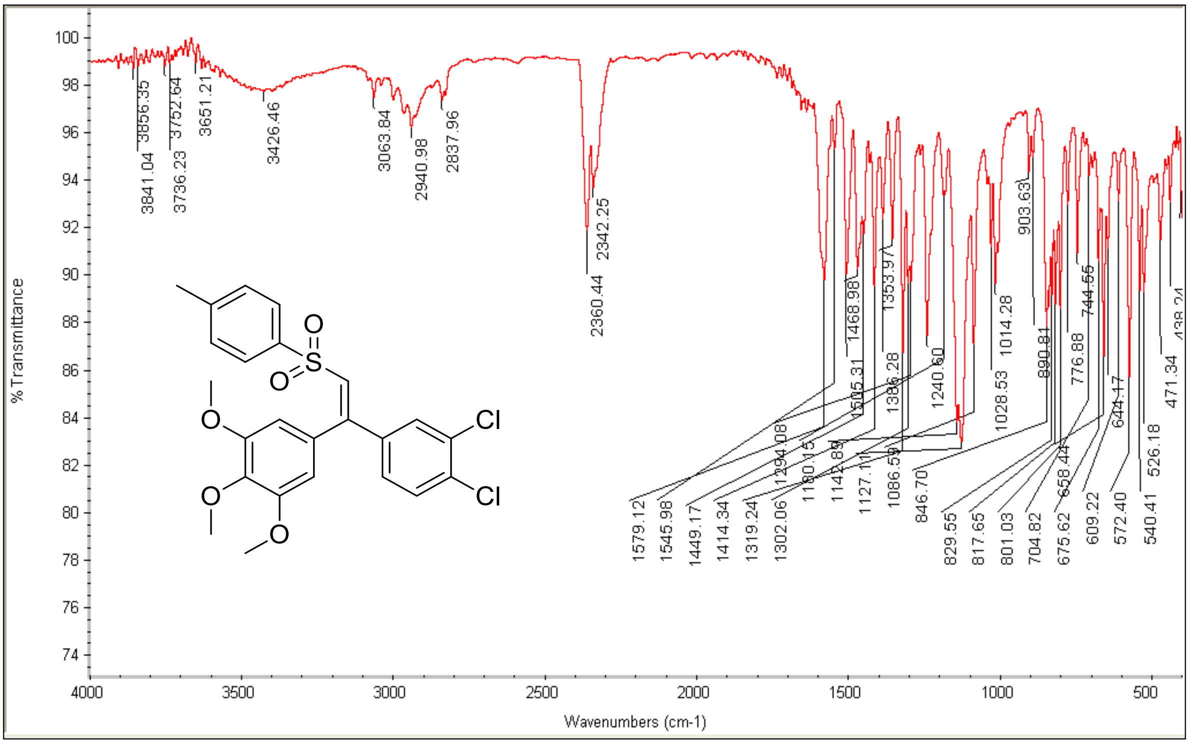

16. 4O, $C_{25}H_{23}NO_5S$, (Z)-4-(2-tosyl-1-(3,4,trimethoxyphenyl)vinyl)benzonitrile

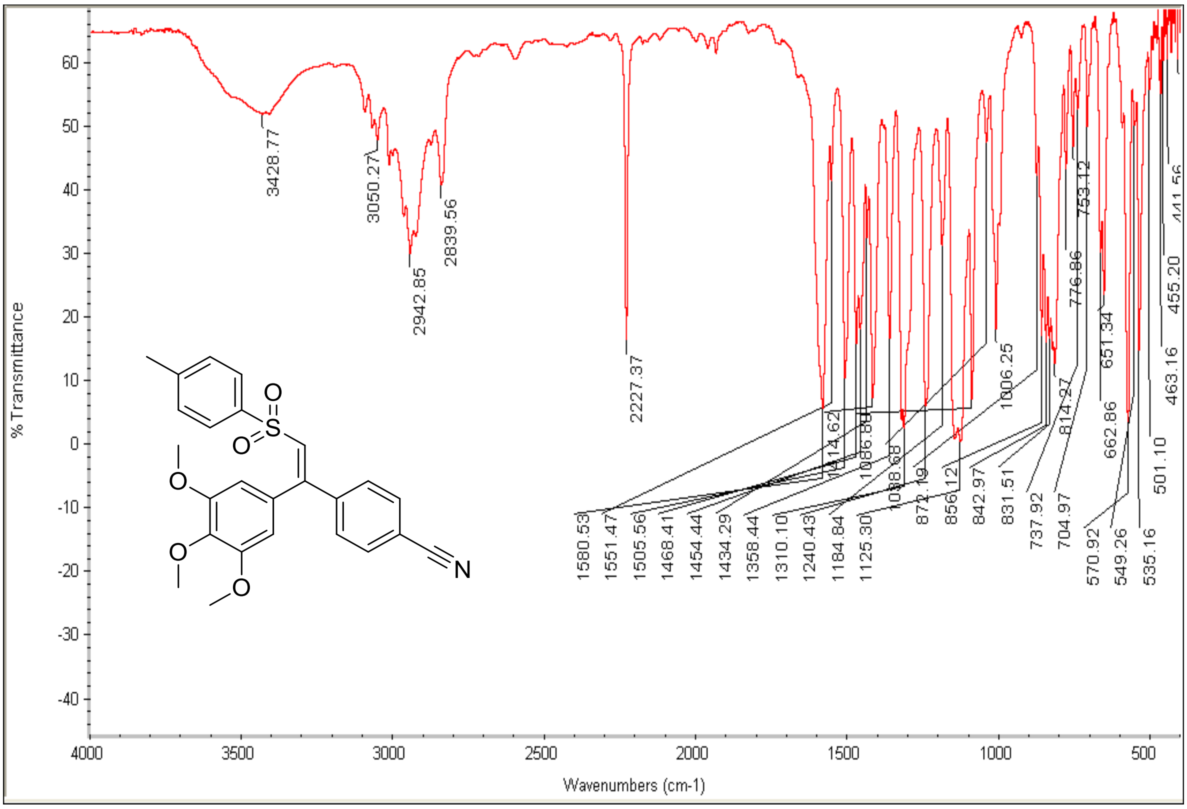

17. 4Q, $C_{25}H_{22}F_4O_5S$, (E)-5-(1-(2-fluoro-3-(trifluoromethyl)phenyl)-2-tosylvinyl)-1,2,3-trimethoxybenzene

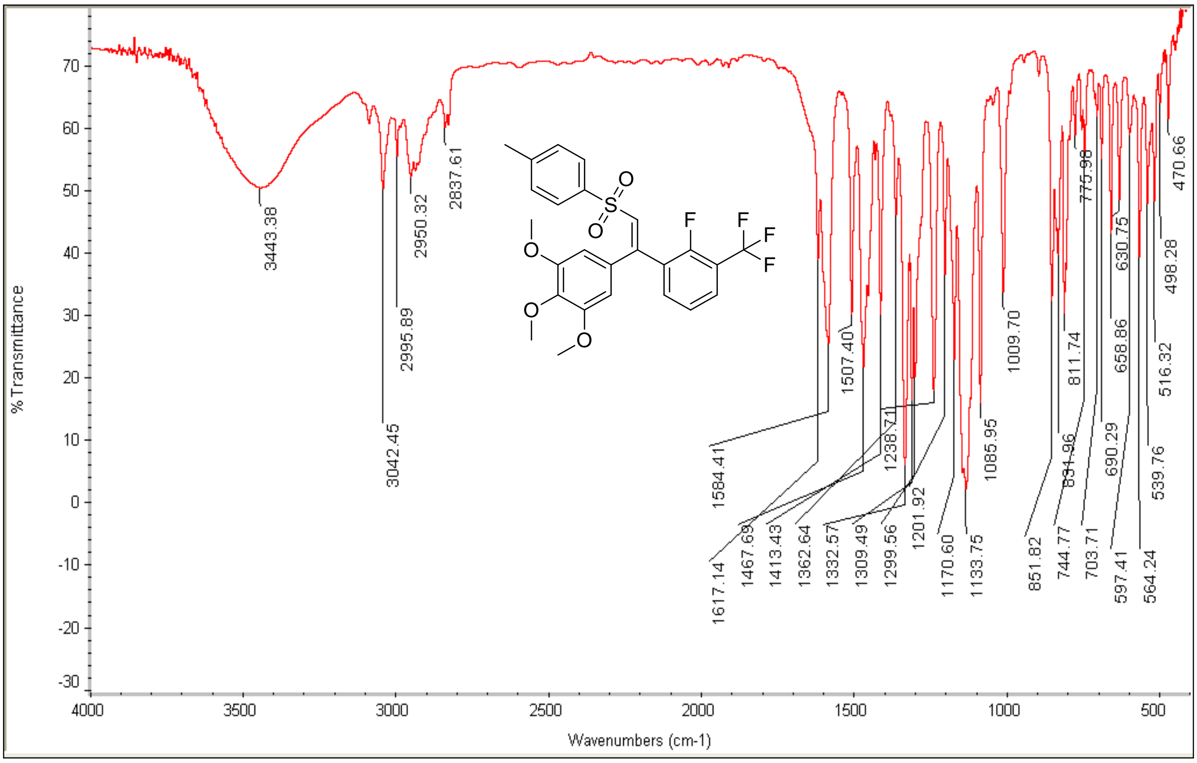

18. 4R, $C_{25}H_{25}FO_5S$, (E)-5-(1-(2-fluoro-5-methylphenyl)-2-tosylvinyl)-1,2,3-

Trimethoxybenzene

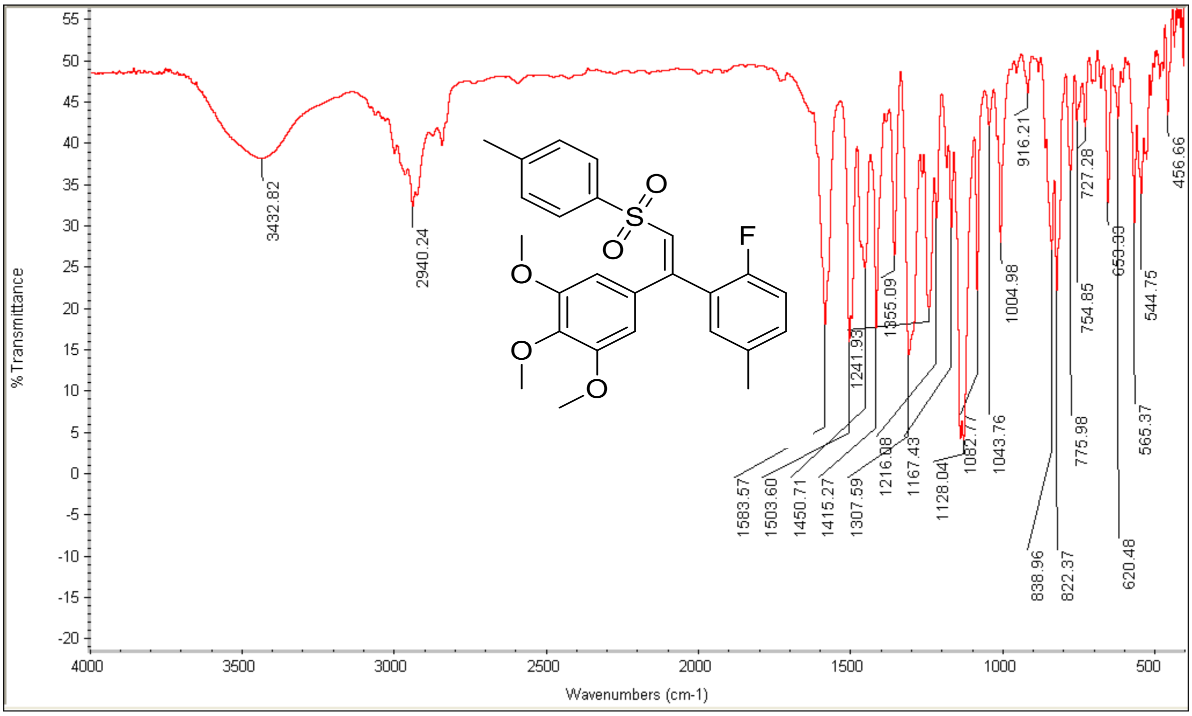

19. 4T, $C_{25}H_{25}FO_5S$, (E)-5-(1-(3-fluoro-4-methylphenyl)-2-tosylvinyl)-1,2,3-trimethoxybenzene

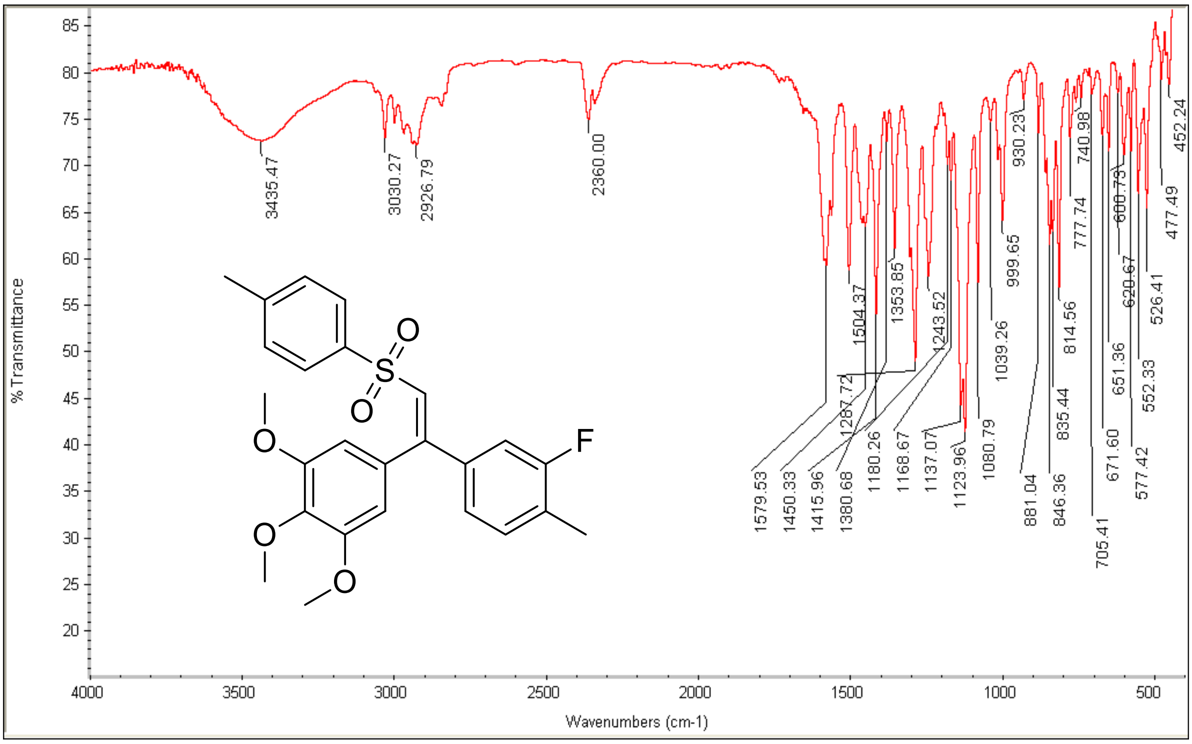

20. 4U, $C_{30}H_{28}O_5S$, (Z)-4-(2-tosyl-1-(3,4,5-trimethoxyphenyl)vinyl)-1,1'-biphenyl

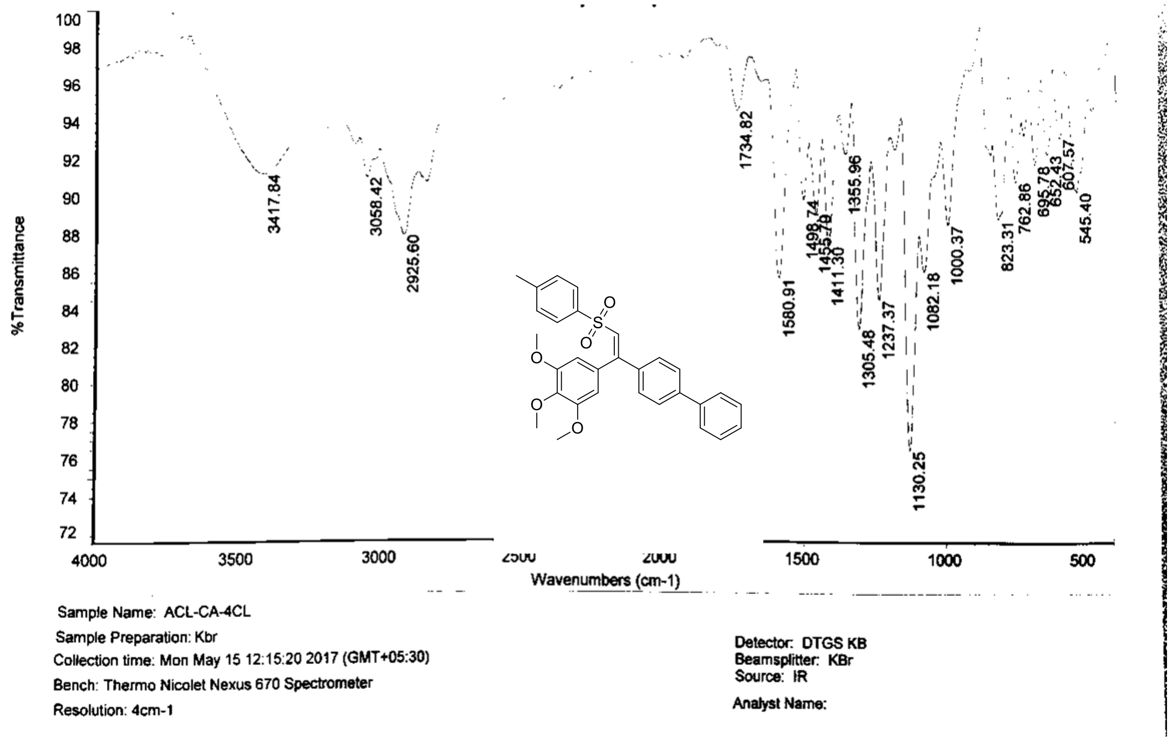

21. 4V, $C_{18}H_{18}O_5S$, 4-methylphenyl (3,4,5-trimethoxyphenyl)ethynyl sulfone

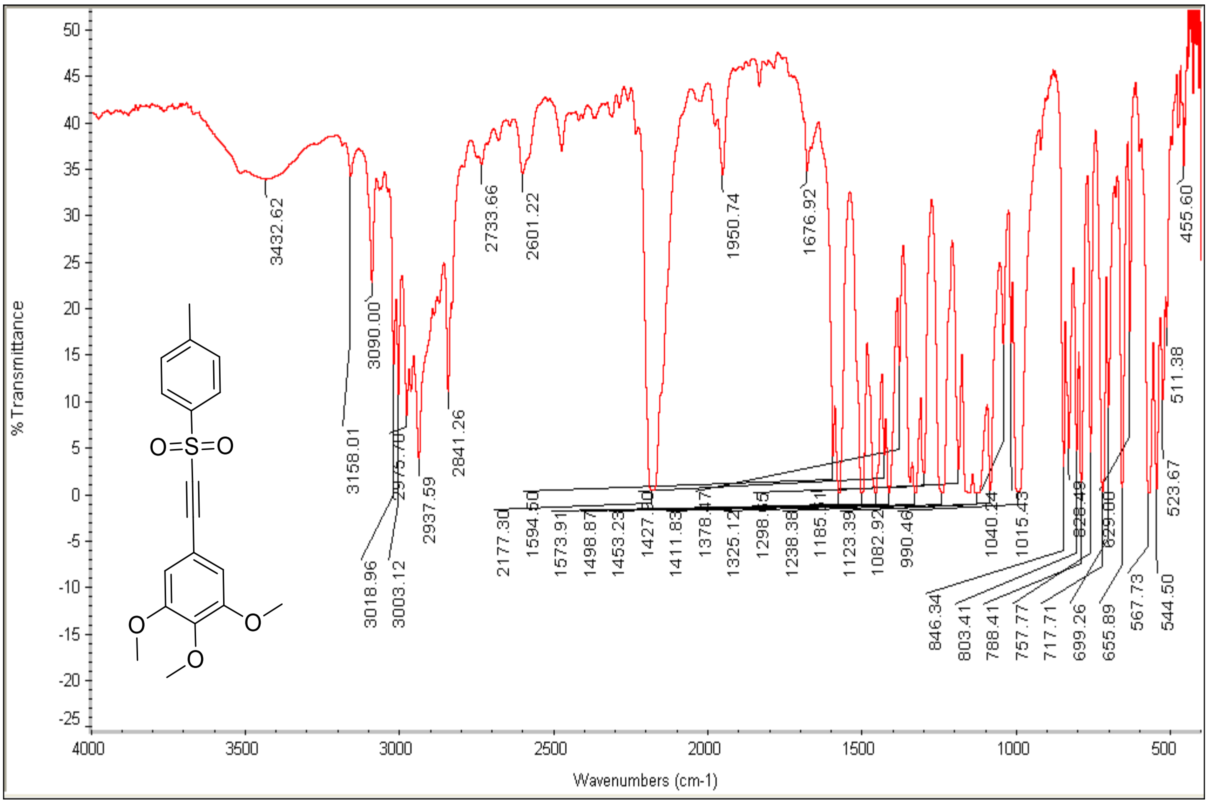

% Transmittance
Wavenumbers (cm-1)
4000
3500
3000
2500
2000
1500
1000
500
O=S=O

DOCKING POSES OF SOME REPRESENTATIVES OF THE LIGANDS

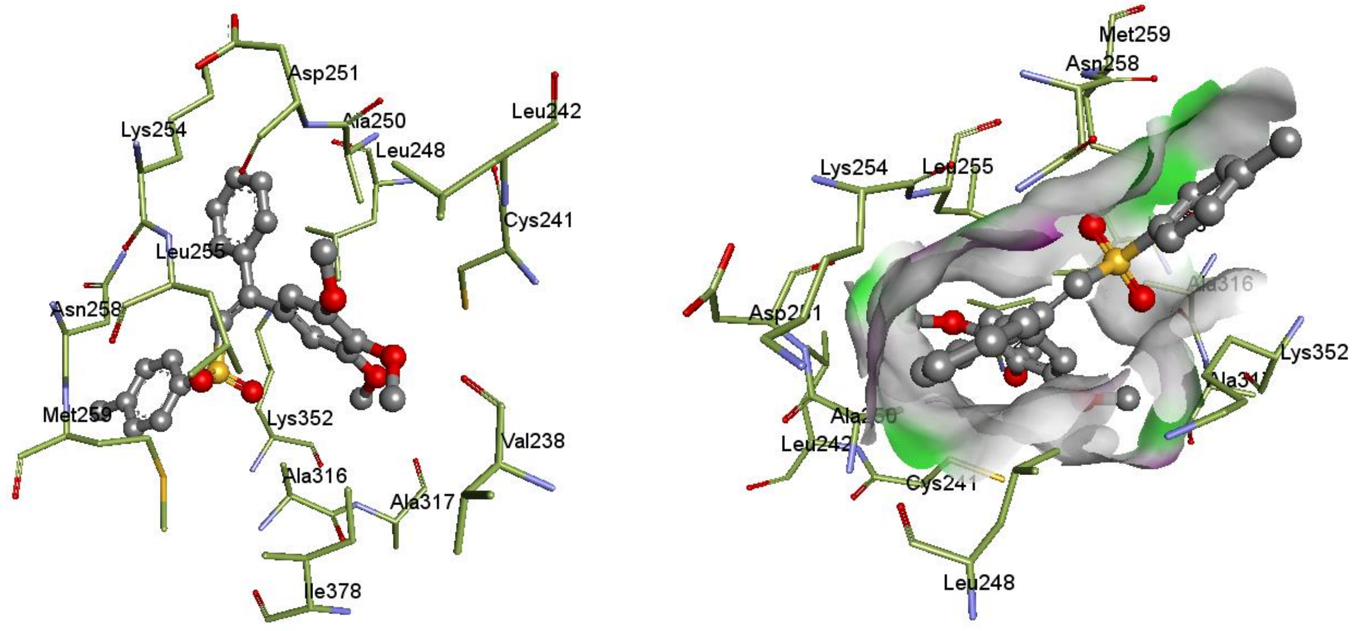

A **B**

Figure a: The binding pose of compound 4A showing residues within 4Å of the docked ligand (A) and a bit of surface rendering (B) to portray the docked ligand within the colchicine binding site of the beta tubulin.

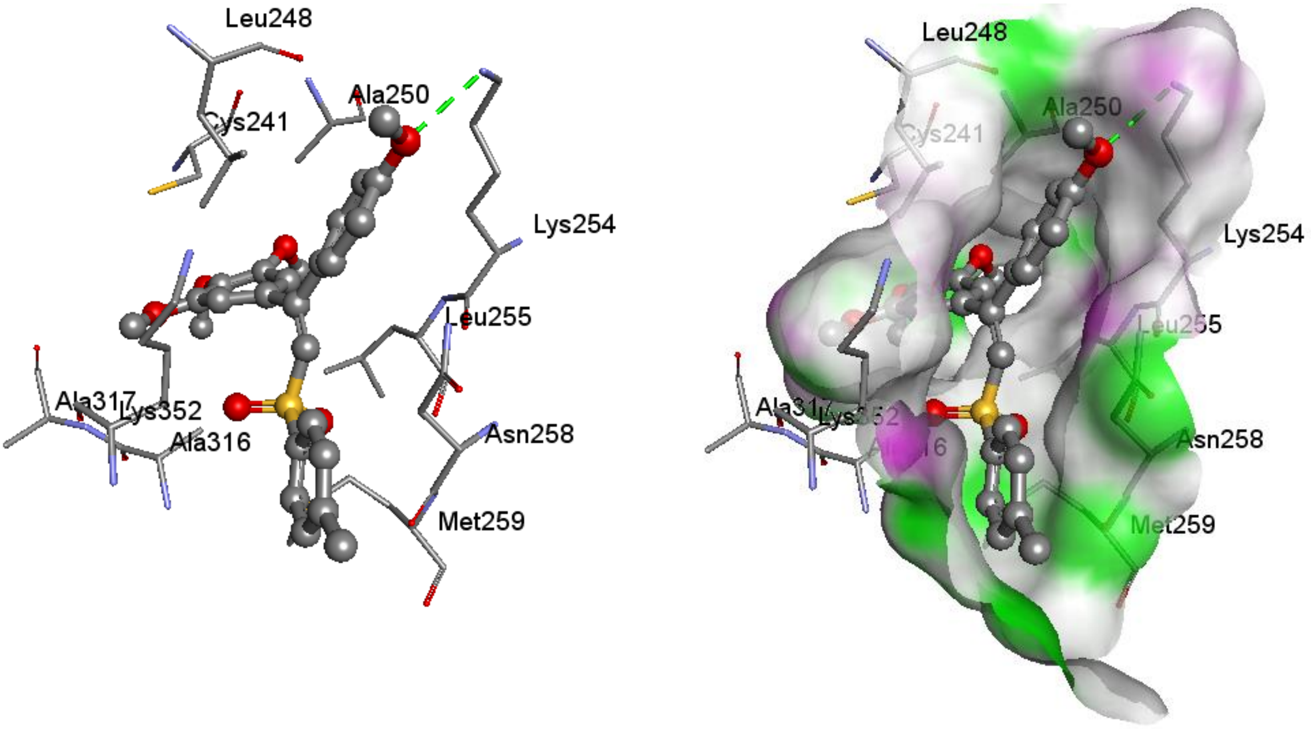

A **B**

Figure b: The binding pose of compound 4B showing residues within 4Å of the docked ligand (A) and a bit of surface rendering (B) to portray the docked ligand within the colchicine binding site of the beta tubulin showing the hydrogen bond with Ala250 as described in Table 3.

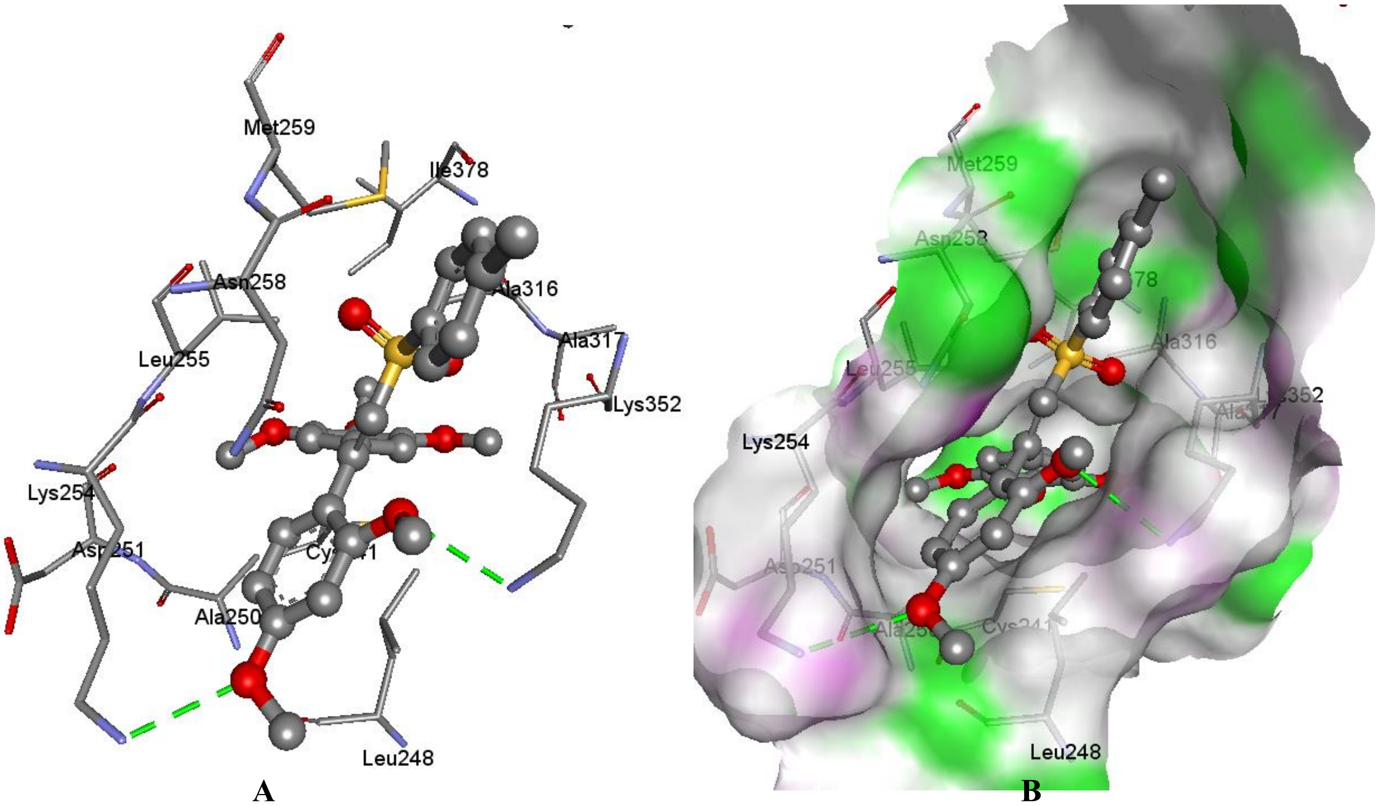

A **B**

Figure c: The binding pose of compound 4C showing residues within 4Å of the docked ligand (A) and a bit of surface rendering (B) to portray the docked ligand within the colchicine binding site of the beta tubulin showing the two hydrogen bonds with Lys352 and Lys254 as described in Table 3.

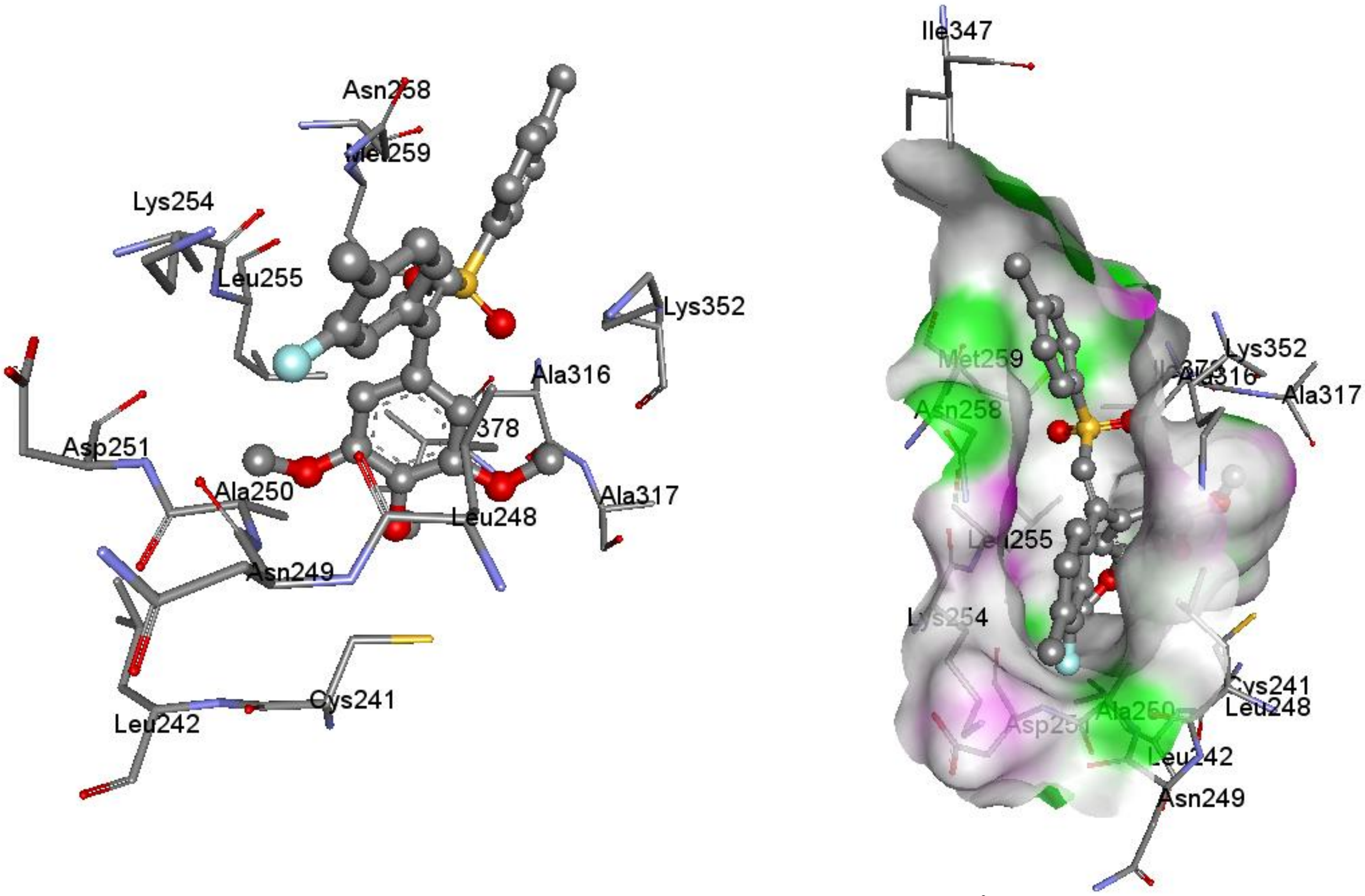

Figure d: The binding pose of compound 4T showing residues within 4Å of the docked ligand (A) and a bit of surface rendering (B) to portray the docked ligand within the colchicine binding site of the beta tubulin.
